# Molecular maps of synovial cells in inflammatory arthritis using an optimized synovial tissue dissociation protocol

**DOI:** 10.1016/j.isci.2024.109707

**Published:** 2024-04-10

**Authors:** Sam G. Edalat, Reto Gerber, Miranda Houtman, Janine Lückgen, Rui Lourenço Teixeira, Maria del Pilar Palacios Cisneros, Tamara Pfanner, Tadeja Kuret, Nadja Ižanc, Raphael Micheroli, Joaquim Polido-Pereira, Fernando Saraiva, Swathi Lingam, Kristina Burki, Blaž Burja, Chantal Pauli, Žiga Rotar, Matija Tomšič, Saša Čučnik, João Eurico Fonseca, Oliver Distler, Ângelo Calado, Vasco C. Romão, Caroline Ospelt, Snežna Sodin-Semrl, Mark D. Robinson, Mojca Frank Bertoncelj

**Affiliations:** 1 Center of Experimental Rheumatology, Department of Rheumatology, University Hospital Zurich and University of Zurich, 8952 Schlieren, Switzerland; 2 Department of Molecular Life Sciences and SIB, Swiss Institute of Bioinformatics, University of Zurich, 8057 Zurich, Switzerland; 3 Team PTA, BioMed X Institute, 69120 Heidelberg, Germany; 4 Instituto de Medicina Molecular (iMM) João Lobo Antunes, Faculdade de Medicina, University of Lisbon, 1649-028 Lisbon, Portugal; 5 Faculdade de Medicina, University of Lisbon, 1649-028 Lisbon, Portugal; 6 Rheumatology Department, Hospital de Santa Maria, Lisbon Academic Medical Center, 1649-028 Lisbon, Portugal; 7 Department of Rheumatology, University Medical Centre Ljubljana, 1000 Ljubljana, Slovenia; 8 Department of Pathology and Molecular Pathology, University Hospital Zurich, 8091 Zurich, Switzerland; 9 Faculty of Medicine, University of Ljubljana, 1000 Ljubljana, Slovenia; 10 Faculty of Pharmacy, University of Ljubljana, 1000 Ljubljana, Slovenia; 11 Faculty of Mathematics, Natural Sciences and Information Technologies, University of Primorska, 6000 Koper, Slovenia

**Keywords:** Health sciences, Technical aspects of cell biology, Omics, Transcriptomics

## Abstract

In this study, we optimized the dissociation of synovial tissue biopsies for single-cell omics studies and created a single-cell atlas of human synovium in inflammatory arthritis. The optimized protocol allowed consistent isolation of highly viable cells from tiny fresh synovial biopsies, minimizing the synovial biopsy drop-out rate. The synovium scRNA-seq atlas contained over 100,000 unsorted synovial cells from 25 synovial tissues affected by inflammatory arthritis, including 16 structural, 11 lymphoid, and 15 myeloid cell clusters. This synovial cell map expanded the diversity of synovial cell types/states, detected synovial neutrophils, and broadened synovial endothelial cell classification. We revealed tissue-resident macrophage subsets with proposed matrix-sensing (FOLR2+COLEC12^high^) and iron-recycling (LYVE1+SLC40A1+) activities and identified fibroblast subsets with proposed functions in cartilage breakdown (SOD2^high^SAA1+SAA2+SDC4+) and extracellular matrix remodeling (SERPINE1+COL5A3+LOXL2+). Our study offers an efficient synovium dissociation method and a reference scRNA-seq resource, that advances the current understanding of synovial cell heterogeneity in inflammatory arthritis.

## Highlights

• Optimized synovial biopsy dissociation for single-cell omics of human synovium
• A single-cell map of fresh-dissociated unsorted synovium in inflammatory arthritis
• An expanded atlas of synovial venous, arterial, and capillary endothelial cells
• Identification of IFITM+ synovial tissue neutrophils

## Graphical abstract

**Figure:**
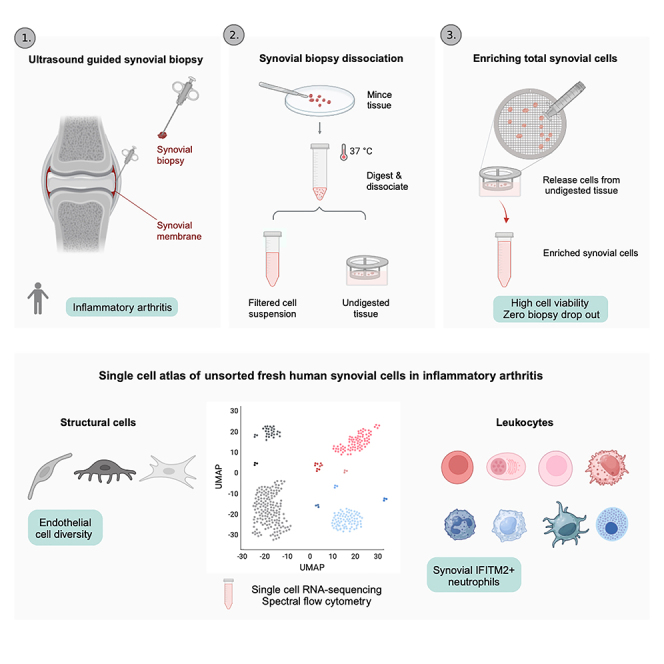

## Introduction

Chronic inflammatory arthritides, such as rheumatoid arthritis (RA) and the family of spondyloarthritides are clinically, radiologically, and molecularly diverse systemic autoimmune diseases that involve peripheral synovial joints and the axial skeleton.^1^^,^^2^ They affect a considerable proportion of the world population and are associated with significant morbidity, lifelong disability, extraarticular manifestations and shorter life expectancy.^3^^,^^4^^,^^5^ Advances in therapy over the last decades have improved patient outcomes considerably and have pointed toward unique but also shared pathogenic pathways in different inflammatory arthritides.^6^^,^^7^ Despite these advances, the management of inflammatory arthritides faces critical challenges, including the development of precision medicine, targeting the stromal cell compartment, and discovering novel pro-resolution drugs.^8^^,^^9^^,^^10^

The synovial tissue, a delicate inner layer of the joint, is the primary disease site in inflammatory arthritides, while spondylarthritis types also affect the entheses.^11^^,^^12^ Given the central role of the synovium in inflammatory arthritides, understanding the identity and heterogeneity of its vital cellular components is essential to improve arthritis management and therapy.^13^^,^^14^ Single-cell RNA-sequencing (scRNA-seq) combined with advancement in minimally invasive synovial biopsy^15^ has expanded our knowledge of synovial cell composition across arthritis conditions^16^^,^^17^ and activity states^18^ (e.g., remission vs. flare). Furthermore, combined with functional experiments, single-cell omics has been instrumental in establishing new concepts in inflammatory arthritis, including the break of the synovial lining macrophage barrier^19^ and divergent specialization of lining and sublining synovial fibroblasts into matrix-destructive and proinflammatory cell types.^20^^,^^21^

Biobanking of viably frozen tissues simplifies retrospective sample selection and allows for centralized tissue processing and scRNA-seq, thereby decreasing the cost, minimizing batch effects, and facilitating multicenter studies.^16^^,^^22^ However, dissociating cryopreserved synovia may lead to inconsistent cell recovery and possible loss of sensitive, short-lived cell populations such as neutrophils, thereby introducing an inherent analysis bias. This bias could be overcome by isolation and analysis of cells from fresh prospectively collected synovial tissue samples. To date, most synovial scRNA-seq studies were carried out on the RA synovium, mainly using viably cryopreserved synovial tissues or pre-sorted synovial cell types^16^^,^^23^ and the protocol for dissociating cryopreserved synovia has been optimized by Donlin et al.^22^ The largest scRNA-seq dataset from the fresh RA synovium, published by Stephenson and colleagues, comprises more than 20000 unsorted scRNA-seq cell profiles from synovectomies of five RA patients.^17^

Our team investigates the pathobiology of human tissues affected by autoimmune diseases, with the first research phase focusing on protocol optimization and establishment of reference tissue scRNA-seq maps.^24^^,^^25^ We have recently published an optimized dissociation protocol and scRNA-seq map of fresh, and cultured human skin.^24^ In the current paper, we presented an optimized protocol for efficient dissociation of prospectively collected fresh synovial biopsies from patients with inflammatory arthritis for single cell omics studies. Furthermore, we created a single-cell reference dataset with annotation of synovial cells from fresh human synovium in inflammatory arthritis, serving as a research resource for the scientific community. Our reference map of the human synovium contains cell subsets/states not previously described in published synovial scRNA-seq atlases, for example IFITIM2+ synovial neutrophils and SAA1+SDC4+ synovial fibroblasts, while significantly expanding the knowledge about synovial endothelial cell (EC) diversity.

## Results

### Optimized dissociation protocol for fresh synovium minimized the sample dropout rate

**Figure 1:**
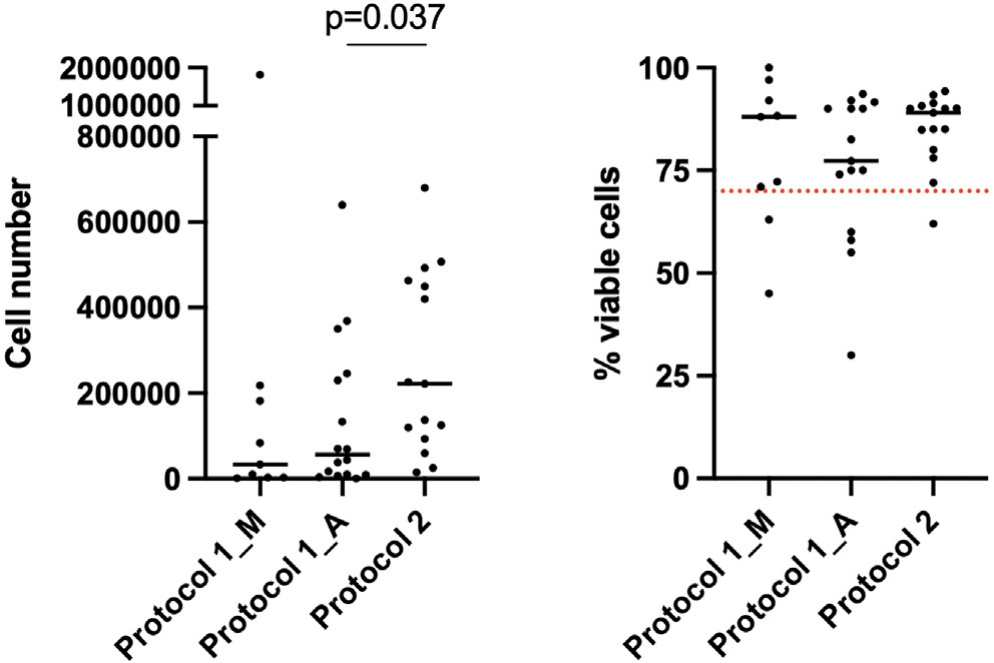
Number and viability of isolated synovial cells varied across synovial cell isolation protocols Protocol 1 – the original protocol of Donlin et al.^22^ Protocol 2 – the optimized protocol derived from Donlin et al. Cells in protocol 1 samples were counted either manually (Protocol 1_M: manual cell counting using the Neubauer chamber) or using Luna-FL dual fluorescence cell counter (Protocol 1_A: automated cell counting. Cells in protocol 2 samples were counted using Luna-FL dual fluorescence cell counter. Data are represented as on a dot plot with median. The red dotted line represents the 70% cell viability treshold, below which the 10x Genomics recommends viable cell sorting before proceeding with single cell encapsulation on 10xGenomics chips. Statistics: comparison of Luna counter outputs of cell yield and viability between the protocols, two-sided Mann-Whitney test, ∗*p* = 0.037.

We utilized the protocol by Donlin et al.^22^ (protocol 1) as a starting reference method for dissociating fresh synovial biopsies in our study. To our knowledge, Donlin’s protocol was the only published synovial tissue dissociation protocol validated across different single-cell omics studies, but primarily optimized for cryopreserved synovia.^22^ Due to low cell yield and/or viability, we lost eight (median 1896 viable cells, range 0–9625) of 26 prospectively collected tissue samples dissociated with protocol 1, resulting in a 31% drop-out rate of prospectively collected patient samples. This data inferred that further optimization of the original protocol 1 might be needed for efficient dissociation of small fresh prospectively collected synovial biopsies. We modified the original protocol 1 to enhance the release of cells from synovial tissue and minimize cell loss by refining the washing steps and volumes of reaction mixes (see STAR Methods for details). The presence of synovial tissue was confirmed in all biopsy samples; representative H&E and immunohistology staining of synovial tissue is shown in Figures S1A and S1B. Krenn synovitis scores,^26^ as a global measure of synovitis, did not significantly differ between the samples dissociated with protocol 1 and our modified protocol 2 (*p* = 0.32, Table 1). We isolated a higher number of cells (*p* = 0.037, Figure 1A) from samples dissociated with protocol 2. Both, the synovial tissue heterogeneity of consecutively recruited patients and the refinements in protocol 2 could contribute to this difference. Notably, synovial cell suspensions from protocol 2 samples demonstrated consistently high cell viability irrespective of isolated synovial cell yields (Figure 1B). In contrast to protocol 1, no samples were lost from protocol 2 dissociations (*p* = 0.039, Chi-square test with Yates’ correction). These results suggested that by slight modifications to the original protocol 1, we could decrease cell loss during sample processing and thus minimize the dropout rate of prospectively collected fresh synovial biopsies for scRNA-seq analyses. The age of patients did not appear to affect the number of isolated cells from synovial biopsies (Figure S1C).

**Table 1:** Histology characteristics and 10x Genomics chemistries for 18 synovial tissue samples from patients with inflammatory arthritis, included in the integrative analysis of scRNA-seq data between the original^22^ (protocol 1) and optimized (protocol 2) protocols

	Protocol 1 (*n* = 6)	Protocol 2 (*n* = 12)	*p* value
Sex∗			
Female	6	7	0.24∗∗∗
Male	0	4	
Age∗			
Median (range)	71.5 (39–81)	56 (20–72)	0.14∗∗∗∗
Krenn synovitis score∗∗			
Median (range)	5 (2–9)	4 (2–6)	0.32∗∗∗∗
Pathotype			
Diffuse myeloid	2	3	NA
Lympho-myeloid	2	6	
Fibroid	1	3	
Unclassified	1	0	
10x Genomics Chemistry			
3′ v3.0	5	0	0.0007∗∗∗∗
3′ v3.1	1	12	

See also Figure S1. Numbers denote the biopsies processed. F: female, M: male, Pathotypes: DM: diffuse myeloid, LM: lymphoid myeloid, F: fibroid, pauci-immune, U: unclassified; ∗gender data not reported for 1 patient in protocol 2 cohort, ∗∗Krenn scoring in the protocol 2 patient cohort based on 10 out of 12 synovial tissues. Statistics: Graph Pad Prism software, ∗∗∗two-sided Fisher’s exact test, ∗∗∗∗two-sided Mann-Whitney t-test, *p* < 0.05 accepted as a statistically significant difference.

Given the minute quantities of biopsied material, we could not process paired tissue fragments from the same donor by both protocols, however, protocol-to-protocol comparison was not the objective of our study. Rather, we aimed to map fresh human synovium in inflammatory arthritis at single-cell resolution and protocol 2 refinements facilitated our study goals.

### The optimized protocol 2 enabled detailed scRNA-seq mapping of the human synovium

We created scRNA-seq data from unsorted synovial cells from 25 highly viable single-cell suspensions. These cell suspensions had median cell viability 90% and 24/25 samples exhibited cell viability ≥70%, the threshold below which viable cell sorting is highly recommended for scRNA-seq studies.^27^

To understand whether our modifications to the original protocol 1 led to differences in the synovial scRNA-seq data quality or cell type enrichment, we integrated scRNA-seq data generated by protocols 1 and 2. The integrative protocol analysis included 18 of 25 synovial tissue samples, thereby avoiding the diagnosis bias between the two protocols (see STAR Methods for details). Patient demographic data, histology characteristics of the 18 synovial samples and utilized 10x Genomics chemistries are shown in Table 1. The generated scRNA-seq data passed standard scRNA-seq QC filters (Figure S2). Specifically, we filtered out cells with a low total number of counts, a low total number of detected genes and a high percentage of mitochondrial counts (Figure S2A). The median cell numbers retained per sample and the number of detected genes per sample were 4467.5 and 14921, respectively (Figure S2B). The distribution of the total number of counts per cell (Figure S2C) and the total number of detected genes per cell (Figure S2D) was similar between samples dissociated using protocols 1 and 2.

**Figure 2:**
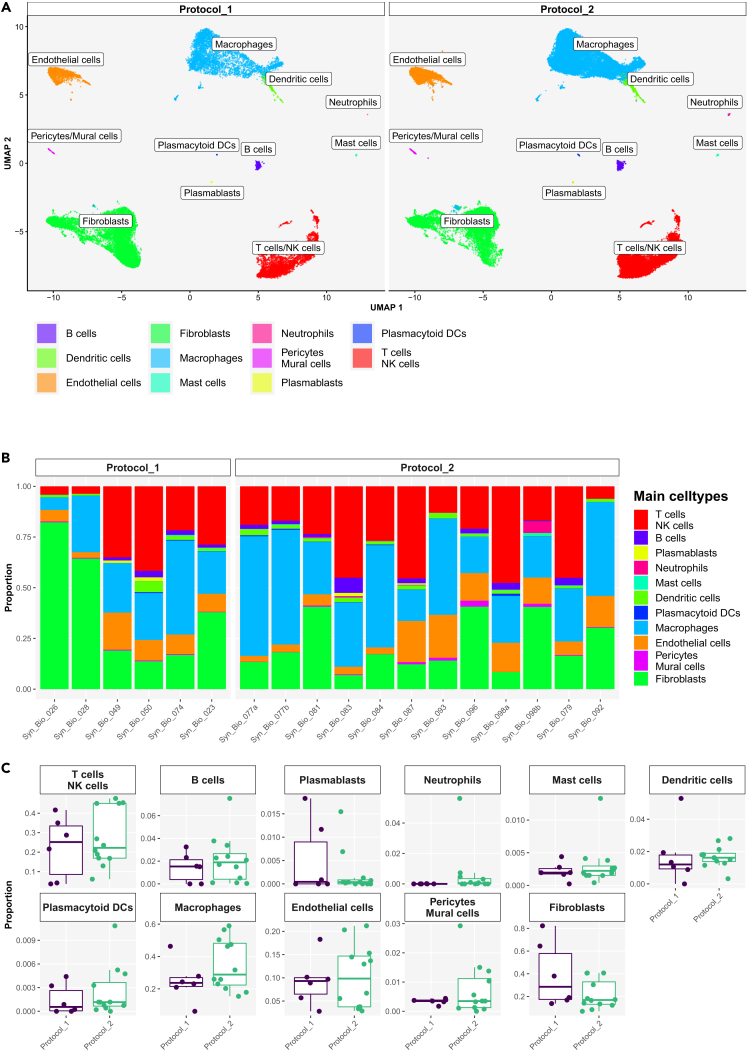
Integrative protocol analysis of scRNA-seq data identified synovial structural, myeloid and lymphoid cell populations (A) UMAPs of the integrated protocol 1 and protocol 2 scRNA-seq dataset with main synovial cell populations colored by main cell type. ScRNA-seq data generated from fresh human synovia dissociated either with the original protocol of Donlin et al.^22^ (protocol 1, *n* = 6) or the optimized protocol (protocol 2, *n* = 12). (B) Bar plots of relative abundances of main cell types per sample per protocol. Neutrophils visible primarily in protocol 2 samples, including SynBio_081, SynBio_083 and Syn_Bio_098b. (C) The proportion of cell types per protocol 1 versus protocol 2; neutrophils were detected primarily in protocol 2 scRNA-seq data, but the difference between the protocols was not statistically significant as calculated by differential abundance analysis in EdgeR. The box plot visualises 5 summary statistics: the median; two hinges, corresponding to the first and the third quartiles; two whiskers. The upper (lower) whisker extends from the hinge to the largest (smallest) value no further than 1.5 ∗ interquartile range from the hinge. Individual dots represent data from different samples. See also Figure S2 and Table S1.

**Figure 3:**
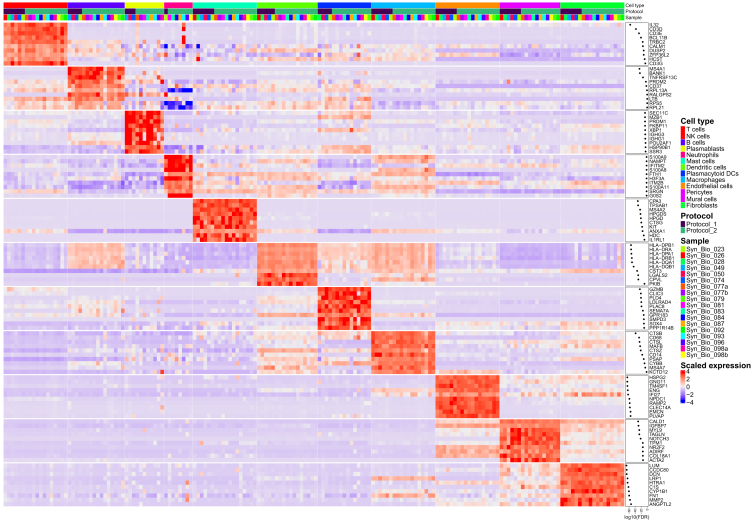
Top marker genes identified main synovial cell types in the integrative protocol scRNA-seq analysis ScRNA-seq data generated from fresh human synovia dissociated either with the original protocol of Donlin et al.^22^ (protocol 1, *n* = 6) or the optimized protocol (protocol 2, *n* = 12). Shown is the heatmap with the top 10 cell type marker genes. Expressions are aggregated by sample and cell type. See also Figures S3–S7.

We generated 76902 high-quality scRNA-seq profiles from 18 synovial tissue samples that formed 11 principal synovial cell populations comprising CD45^+^ lymphoid, CD45^+^ myeloid and CD45^−^structural synovial cell subsets (Figure 2A). The main synovial cell populations were heterogeneously distributed across patients’ synovia (Figure 2B); their abundances, however, did not differ significantly as calculated by EdgeR (see STAR Methods) between protocols 1 and 2 (Figure 2C). The heatmap with top gene markers, specific for the 11 principal synovial cell populations, is shown in Figure 3. Specifically, we identified synovial CD3E + T cells, CD79A + CD79B+ B cells, CD79A + XBP1+ B cells/plasmablasts (Figure S3A), CD14^+^ macrophages, LILRA4+ GZMB+ ITM2C+ plasmacytoid dendritic cells (DCs) (Figure S3B), CD1C+ FCER1A + DCs, CLEC9A + DCs, CPA3+ mast cells, IFITM2^high^ neutrophils (Figure S3C), VWF+ endothelial cells (ECs), ACTA2+ TAGLN+ pericytes/mural cells and COL1A1+ synovial fibroblasts (Figure S3D). CD1C+ FCER1A+ and CLEC9A + DCs co-clustered with synovial macrophages (Figures S3B and S3C). Synovial T/NK cell, macrophage, EC, and synovial fibroblast clusters represented 4–48%, 6–59%, 3–21% and 7–82% of all synovial cells across the patient samples, respectively, constituting the most abundant synovial cell populations (Figure 2). Looking closer at the T/NK cell cluster, we identified CD4^+^ T cells, CD3^+^ CD8A^+/−^ NKG7+ T cells, CD3^neg^ CD8A^neg^ NKG7+ NK cells and CD3^neg^ CD8A^neg^ NKG7^neg^ cells (Figure S4A). Macrophage population consisted of tissue-resident synovial macrophages (expressing *MERTK, TREM2, CD206/MRC1* genes, Figure S4B) and infiltrating synovial macrophages (expressing *IL1B, CD48* genes, Figure S4B), while structural cells included VWF^low^LYVE1^high^CCL21^high^ lymphatic ECs, VWF^high^ vascular ECs (Figure S4C), PRG4^high^THY1^low^ lining synovial fibroblasts, and THY1+ PRG4^low^ sublining synovial fibroblasts expressing *GGT5, CXCL12 or DKK3* genes (Figure S4D).

Neutrophils were detected in 17% (1/6) of protocol 1 scRNA-seq samples and 50% (6/12) of protocol 2 samples (Table S1; Figure S1B). Among sequenced samples, sample SB-081, SB-083 and especially sample SB-098B demonstrated a higher proportion of neutrophils within a total population of synovial cells (Figure 2B; Table S1). Top neutrophil genes, including canonical and maturation neutrophil markers (*NAMPT, SOD2, CSF3R, FCGR3B, G02,* S100A8, S100A9), are shown in Figure S5. Upon histological analysis of the tissue, we demonstrated the presence of neutrophils in 66% of protocol 1 samples and 91% of protocol 2 samples (Table S1; Figure S1). In general, neutrophils were rather scarce and commonly focally distributed within or between biopsy fragments (Table S1). Histology and scRNA-seq analyses matched in 50% when detecting synovial neutrophil presence/absence (Table S1).

In summary, we could map all major and minor synovial cell populations with both protocols using scRNA-seq and flow cytometry analyses. Our results inferred that our optimized protocol 2 kept the comprehensiveness of the original protocol 1 in detecting synovial cell diversity, while minimizing the dropout rate of valuable patient samples.

### Flow cytometry detected diverse synovial cell populations in protocol 2 cell suspensions

Next, we assessed whether synovial cells, dissociated with protocol 2, can also be analyzed with flow cytometry. These proof-of-principle experiments included analysis of total unsorted synovial cells using two different flow cytometry technologies. We collected synovial biopsies from two patients with arthritis (Table S2) at the Rheumatology Department, Hospital de Santa Maria, Lisbon Academic Medical Center, Lisbon, Portugal. Synovial cells from a patient with septic arthritis were analyzed immediately after dissociation using multicolor flow cytometry (Figure S6, *n* = 1). In contrast, cells from a patient with early RA were fixed and shipped to BioMed X Institute, Heidelberg, for spectral flow cytometry analysis (Figure S7, *n* = 1). Flow cytometry analyses confirmed successful isolation of synovial CD45^−^structural cells, as well as CD45^+^ myeloid and lymphoid cell populations, including PDPN+ synovial fibroblasts, CD31^+^ ECs (Figure S7), CD11B+ CD64^+^ or CD14^+^ macrophages, CD19^+^ B cells, CD3^+^ T lymphocytes and their CD4^+^ and CD8^+^ subsets (Figures S6 and S7). Multicolor flow cytometry demonstrated good synovial cell viability (80%) (Figure S6).

### Generation of a single-cell reference map of fresh human synovium in inflammatory arthritis

In the next step, we integrated the scRNA-seq data from 25 synovial tissue samples of patients with inflammatory arthritis (see STAR Methods for details), generated either with protocol 1 or 2, to build a single-cell reference map of freshly dissociated human synovium in inflammatory arthritis. Table 2 shows patient demographic and therapy data, histology characteristics and 10x Genomics chemistries for 25 synovial samples included in the human synovium reference map analysis. The QC analysis of the scRNA-seq data from the 25 samples is shown in Figure S8. After filtering out the cells with a low total number of counts, a low total number of detected genes and a high percentage of mitochondrial counts (Figure S8A), we kept 102758 single-cell profiles (Figure S8B). The distribution of the total number of counts per cell and the total number of detected genes per cell across the 25 synovial tissue samples is shown in Figures S8C and S8D.

**Table 2:** Histology characteristics and 10x Genomics chemistries for 25 synovial tissue samples from patients with inflammatory arthritis, included in the generation of the single-cell reference map of the fresh human synovium

	Number of biopsies
Protocol	
1: Original, Donlin L. et al.^22^	10
2: Optimized, Edalat SG. et al.	15
Sex∗	
Female	18
Male	5
Age	
Median (range)	55.5 (20–81)

**Therapy at biopsy∗∗**	

No therapy	3
Corticosteroid	1
Corticosteroid + cDMARD	1
cDMARDs	2
bDMARDs	3
cDMARD + bDMARD	2
cDMARD + JAKi	3
Corticosteroid + cDMARD + JAKi	1
Antibiotics	2
Refractory arthritis∗∗∗	6
Krenn synovitis score∗∗∗∗	
Median (range)	4 (2–9)
Pathotype	
Diffuse myeloid	10
Lympho-myeloid	9
Fibroid	5
Unclassified	1
10x Genomics Chemistry	
3′ v3.0	5
3′ v3.1	20

See also Figure S1. Numbers denote the biopsies processed. F: female, M: male, DM: diffuse myeloid, LM: lymphoid myeloid, F: fibroid, pauci-immune, U: unclassified. ∗Gender data not reported for 2 patients in protocol 2 cohort, ∗∗The values indicate the number of patients receiving different disease-modifying antirheumatic drugs (DMARDs) at the time of biopsy with data missing for 5 patients. Conventional DMARDs (cDMARDs) included methotrexate, leflunomide, salazopyrin, sulfasalazine and plaquenil. Biological DMARDs (bDMARDs) included anti-TNF, anti-IL-6, anti-IL-17A, anti -IL-12/IL-23 and anti-IL-23 therapies, and therapeutic fusion proteins (soluble TNFa receptor, CTLA4). Janus kinase inhibitors (JAKi) included baricitinib and tofacitinib. Antibiotics included vibramycin and doxycycline. ∗∗∗Indicated is the number of patients failing multiple cDMARDs and bDMARD/JAKi in the period before biopsy data about treatment history are missing for 6 patients. ∗∗∗∗Krenn scoring, based on 21 out of 25 synovial tissues.

A single-cell map of fresh synovium comprised eleven principal synovial cell clusters (Figure S9A), which replicated the lymphoid, myeloid, and stromal cell types identified in the integrative protocol analysis (Figure 2A). The variable distribution of these principal cell types across patient synovia is presented in Figures S9B and S9C. The top 10 marker genes defining these principal lymphoid, myeloid and stromal synovial cell populations are shown in Figure S10. T lymphocytes, macrophages, synovial fibroblasts and ECs represented the most abundant cell types (Figure S9). We further sub clustered these cell populations to understand their subset diversity and distribution in the synovium from patients with inflammatory arthritis.

### Subcluster analysis uncovered the diversity of T cells in freshly dissociated human synovium

**Figure 4:**
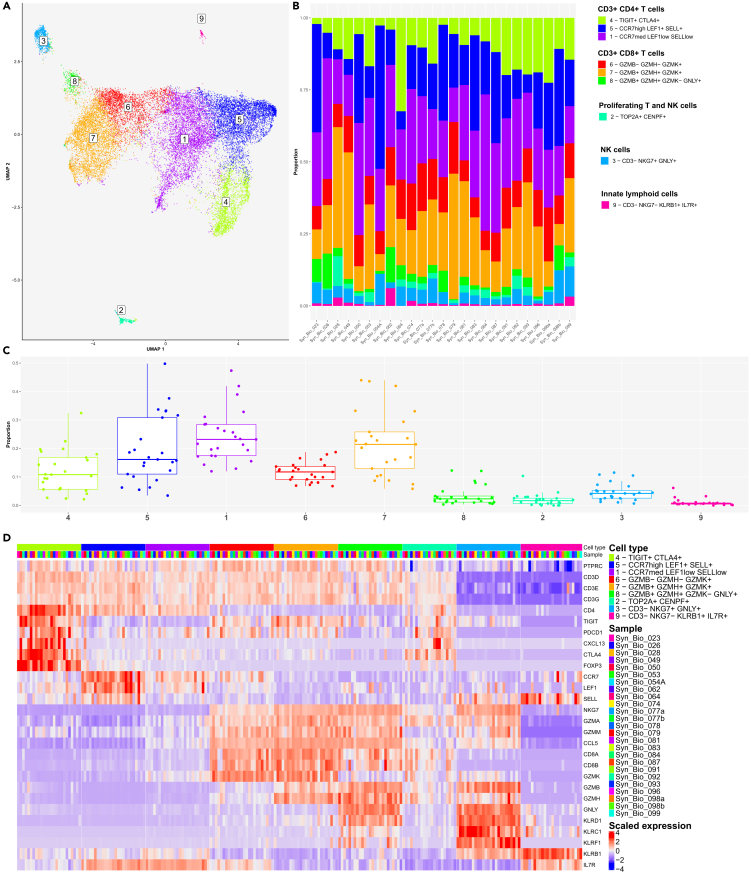
Subcluster analysis demonstrated heterogeneity of synovial T cell, natural killer (NK) cell and innate lymphoid cell (ILC) populations in the integrated synovial single-cell map datasets from 25 fresh-dissociated synovial biopsies of patients with inflammatory arthritis (A) UMAP with annotated T cell, NK cell and ILC clusters colored by cell cluster, (B) a bar plot of T, NK and ILC cell cluster abundances across synovial biopsies of patients with inflammatory arthritis colored by cell cluster (right). (C) The proportions of the nine cell clusters, (D) the heatmap of the top T cell, NK cell and ILC marker genes. (C) The box plot visualises 5 different summary statistics: the median, two hinges, corresponding to the first and the third quartiles, two whiskers. The upper (lower) whisker extends from the hinge to the largest (smallest) value no further than 1.5∗ interquartile range from the hinge. Individual dots represent data from different samples. See also Figures S8–S11.

We profiled 23169 cells comprising six CD3^+^ T cell clusters (1, 4–8), one cluster of CD3^neg^ NK cells (cluster 3), one cluster of proliferating TOP2A+ CENPF+ T cells and NK cells (cluster 2, Figure S11A) and a small cluster of CD3^neg^ NKG7^neg^ KLRB1+ IL7R + cells, suggestive of innate lymphoid cells (cluster 9) (Figure 4A). The percent distribution of T cell, NK cell and innate lymphoid cell clusters varied considerably across patient’s synovia (Figures 4B and 4C). The selected key marker and cluster-enriched genes of T cells, NK cells and innate lymphoid cells are presented in Figures 4D and S11B. Specifically, the CD4^+^ TIGIT^low/neg^ T cell clusters (1, 5) differed in the expression of *CCR7*, *LEF1*, and *SELL* transcripts (Figures 4D and S11C). Meanwhile, CD4^+^ TIGIT+ CTLA4+ cluster 4 contained FOXP3+ and CXCL13+ PDCD1+ cells (Figures 4D and S11C), which could represent regulatory and peripheral/follicular CD4^+^ helper T cell phenotypes,^16^ respectively. The CD8^+^ CCL5+ T lymphocytes comprised NKG7^med^ GZMK+ cells (cluster 6), cytotoxic NKG7^high^ GNLY^high^ GZMK^neg^ (cluster 8) and NKG7^high^ GNLY^neg^ GZMK^high^ (cluster 7) cells (Figures 4A–4C and S11D). CD8^+^ T cell subsets varied in the expression of *GZMB* and *GZMH* transcripts (Figures 4D and S11D). The NKG7^high^ GNLY^high^ NK cells (cluster 3) were GZMB^high^ and expressed abundantly Killer Cell Lectin like Receptor (*KLRC1*, *KLRD1*, and *KLRF1*) transcripts (Figure 4D).

### Subcluster analysis unveiled the synovial fibroblast heterogeneity in freshly dissociated human synovium

**Figure 5:**
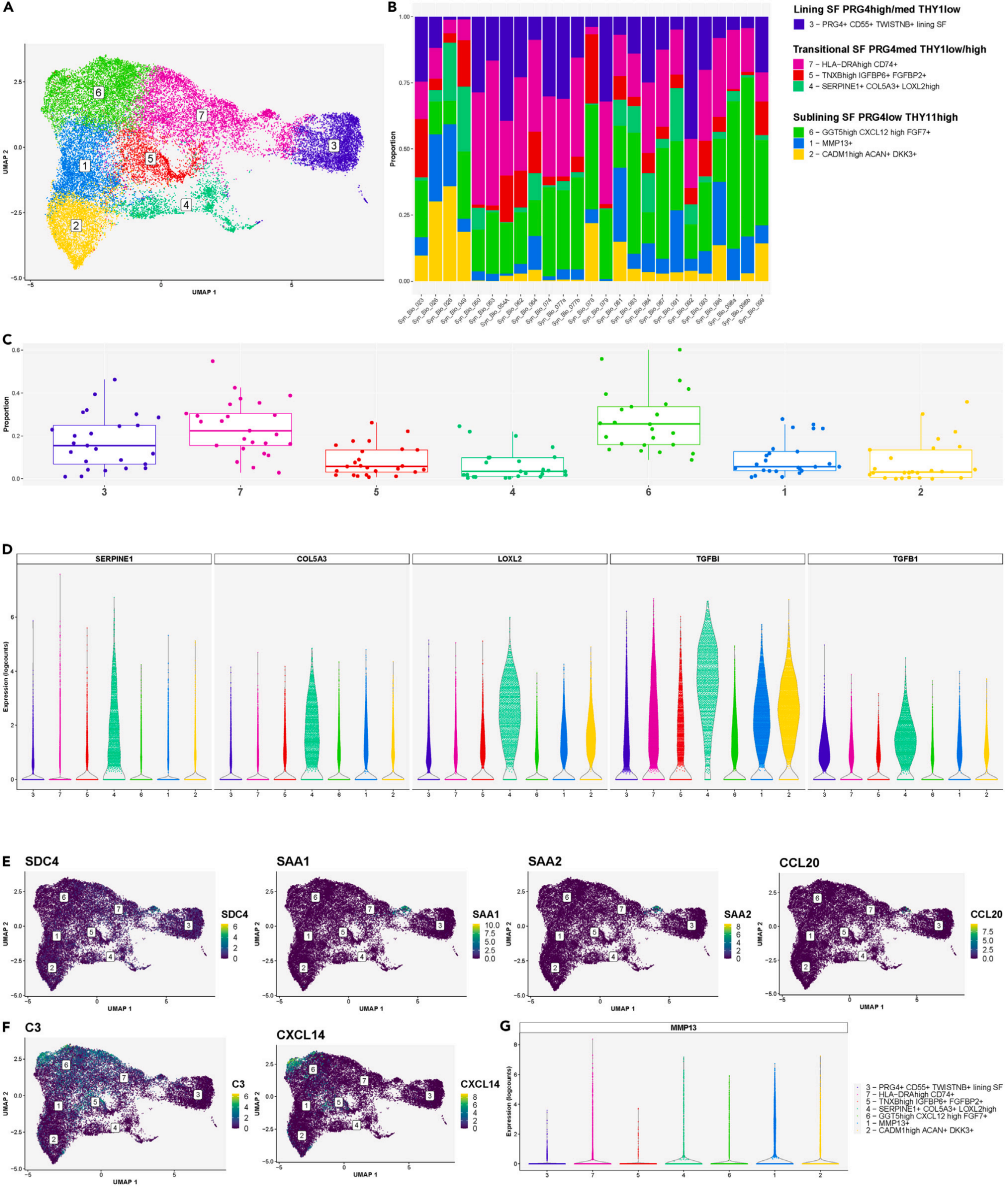
Subcluster analysis demonstrated heterogeneity of synovial fibroblast population within the integrated synovial single-cell map dataset of inflammatory arthritis from 25 fresh-dissociated synovial biopsies of patients with inflammatory arthritis (A) UMAP with annotated synovial fibroblast clusters colored by cell clusters. (B) A bar plot of synovial fibroblast cluster abundances across synovial biopsy samples of patients with inflammatory arthritis colored by cell cluster. (C) The proportions of the seven synovial fibroblast clusters across 25 synovial samples from patients with inflammatory arthritis. The box plot visualises 5 summary statistics: the median; two hinges, corresponding to the first and the third quartiles; two whiskers. The upper (lower) whisker extends from the hinge to the largest (smallest) value no further then 1.5∗ interquartile range from the hinge. Individual dots represent data from different samples. (D) Violin plots showing the top enriched matrix-remodeling genes (*LOXL2, TGFBI, TGFB1*), expressed by the SERPINE1+ COL5A3+ synovial fibroblasts (cluster 4). (E) Violin plots showing the top enriched genes, expressed by the SOD2^high^ synovial fibroblast subpopulation within cluster 7, including *SDC4* and proinflammatory genes (*SAA1, SAA2, CCL20*). (F) UMAPs demonstrating *C3* and *CXCL14* mRNA expression in synovial fibroblasts, identifying a small population of C3^high^ CXCL14^high^ cells within the sublining GGT5^high^ CXCL12^high^ synovial fibroblast subset (cluster 6). (G) A violin plot demonstrating *MMP13* mRNA expression in synovial fibroblasts in cluster 1. See also Figures S8–S10 and S12–S14.

Through our analysis, we identified seven COL1A1+ synovial fibroblast clusters (Figure 5A) and further classified these cells based on their expression of the lining (PRG4) and sublining (THY1) fibroblast markers^16^^,^^21^^,^^23^ (Figures 5A and S12A). Overall, the abundance of synovial fibroblast clusters varied significantly across patient synovia (Figures 5B and 5C). The seven synovial fibroblast clusters represented lining PRG4^high^ THY1^low^ (cluster 3) synovial fibroblasts, transitional PRG4^med^ THY1^med/high^ synovial fibroblasts (clusters 4, 5, 7) and PRG4^low^ THY1^high^ sublining synovial fibroblasts (clusters 1, 2, 6) (Figures 5A and S12A). The proliferating TOP2A+ CENPF+ synovial fibroblasts co-clustered with cluster 4 fibroblasts and contained both PRG4^high^ and THY1+ cells (Figure S12B). The PRG4^high^ lining synovial fibroblasts were reported as enriched in osteoarthritis synovia^16^ and associated with matrix-degrading activities,^20^^,^^21^ while HLA-DRA+ sublining cells were found abundant in leukocyte-rich RA.^16^

To further understand the synovial fibroblast transcriptional diversity, we analyzed the expression of known fibroblast subset marker genes alongside top enriched genes between the seven fibroblast clusters (Figure S13). The lining fibroblasts (cluster 3) expressed *CD55*, *ITGB8*, *MMP3*, *MMP1*, and *TWISTNB*^28^ transcripts (Figure S13). These cells were GAS6^neg/low^ (Figure S13) as previously reported.^16^^,^^18^ A subset of lining fibroblasts was enriched for *CLIC5* and *HBEGF* expression (Figure S14A). Cluster 4 represented SERPINE1+ COL5A3^high^ fibroblasts, characterized by an extracellular matrix remodeling gene signature (*LOXL2, TGFBI*, and *TGFB1*) (Figures 5D and S13). The HLA-DRA^high^ cells (cluster 7) expressed transcripts linked to MHC class II antigen presentation (*HLA-DRA, HLA-DRB1, HLA-DPB1*, and *CD74*, Figure S14B).^16^ *HLA-DR* and *CD74* transcripts were expressed also in lining synovial fibroblasts. Cluster 7 contained a small population of HLA-DRA^neg/low^ SOD2^high^ fibroblasts expressing syndecan 4 (*SDC4*) and proinflammatory genes *SAA1* and *SAA2* genes (Figure 5E). SDC4 was reported to be prominently expressed in synovial lining cells in RA synovia.^29^ Cluster 5 included TNXB^high^ IGFBP6+ FGFBP2+ cells, expressing *CD34* and *DPP4* transcripts (Figure S13). Among the sublining fibroblast clusters, GGT5^high^ CXCL12^high^ FGF7+ fibroblasts (cluster 6) contained NOTCH3-expressing cells, IL6-expressing cells (Figures S13 and S14C) and a small population of C3^high^ CXCL14^high^ fibroblasts (Figures 5F and S13). NOTCH3 and GGT5 expression was associated with periarterial localization of synovial fibroblasts.^23^ A subset of synovial fibroblasts in cluster 1 was enriched in *MMP13* (Figures 5A–5G and S13); their transcriptome, however overlapped with cluster 6 cells. Finally, cluster 2 cells represented CADM1^high^ ACAN+ DKK3+ sublining fibroblasts (Figures 5A and S13). Deconvolution analysis of synovial RNA-seq data demonstrated enrichment of DKK3+ synovial fibroblasts in patients with drug-refractory RA.^30^

### Subcluster analysis identified synovial macrophage subset heterogeneity

**Figure 6:**
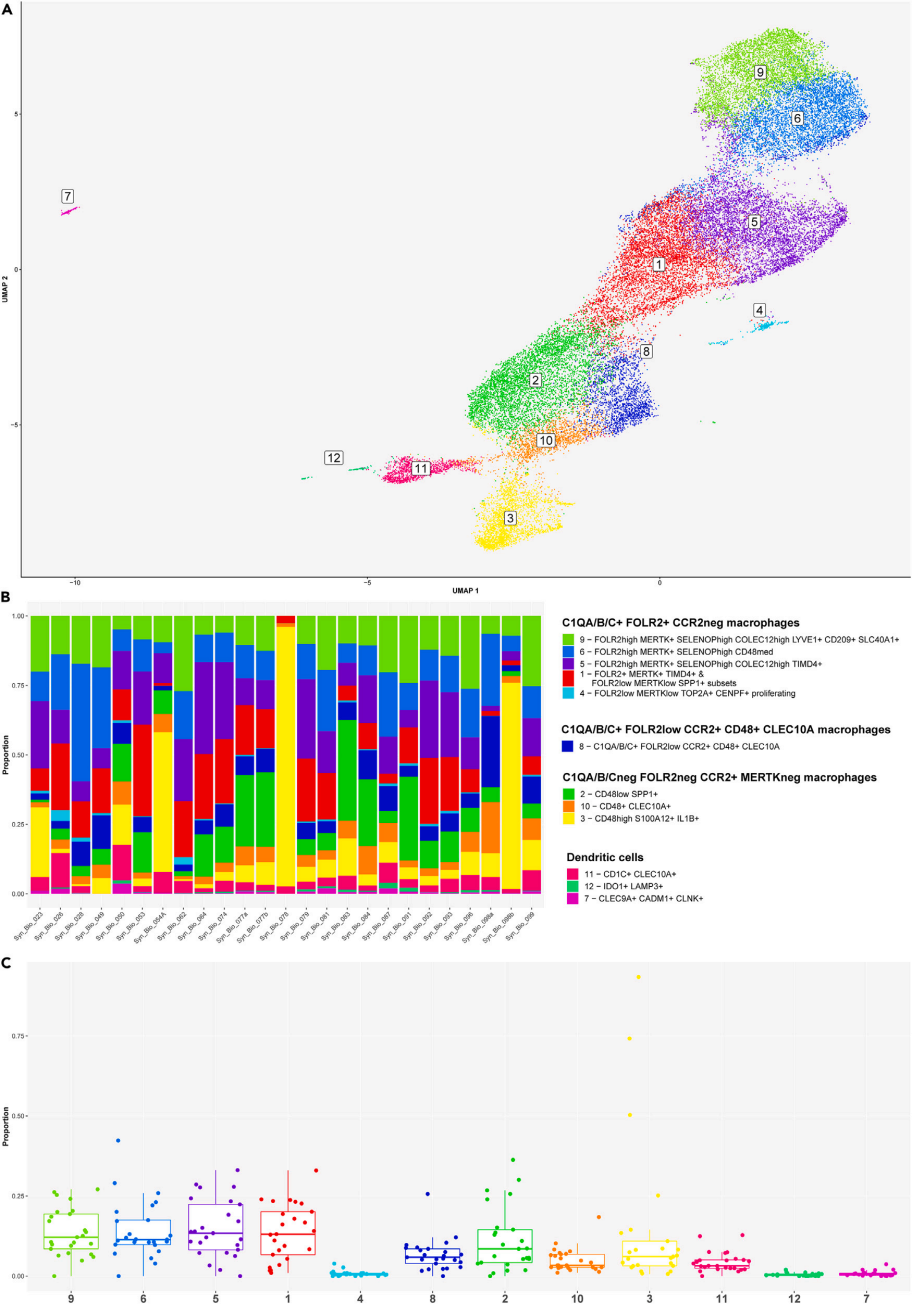
Subcluster analysis demonstrated heterogeneity of synovial macrophage and dendritic cell (DC) populations in the integrated synovial single-cell map dataset from 25 fresh-dissociated synovial biopsies of patients with inflammatory arthritis (A) UMAP of 3 dendritic cell (DC) clusters and nine synovial macrophage clusters colored by cell cluster. (B) Bar plots of relative abundances of three DC and nine synovial macrophage clusters across 25 patient samples colored by cell cluster. (C) The proportion of three DC and nine macrophage clusters across 25 synovial tissue samples from patients with inflammatory arthritis. The box plot visualises 5 summary statistics: the median; two hinges, corresponding to the first and the third quartiles, two whiskers. The upper (lower) whisker extends from the hinge to the largest (smallest) value no further then 1.5 ∗ interquartile range from the hinge. Individual dots represent data from different samples. See also Figures S8–S10 and S15.

The synovial macrophage and DC cluster contained 35659 CD45^+^ single cells distributed into CD14^low/neg^ CD64^low/neg^ CD11b^low/neg^ DC and CD14^+^ CD64^+^ CD11b+ macrophage clusters (Figures 6A and S15). The heatmap in Figure S15 shows key marker and cluster-enriched genes expressed in macrophage and DC clusters.

DC clusters included CD1C+ FCER1A + CLEC10A + DCs (cluster 11), CD83^+^ LAMP3+ IDO1+ DCs (cluster 12) and CLEC9A + CADM1+ CLNK+ DCs (cluster 7) (Figures 6A and S15). We annotated the nine CD14^+^ CD64^+^ CD11b+ macrophage clusters based on their expression of CCR2, FOLR2 and complement chain 1q (C1QA, C1QB, C1QC) genes (Figures 6A and S15). The expression of CCR2 and FOLR2 was linked to tissue-infiltrating and tissue-resident macrophage subsets, respectively.^31^ Synovial macrophages grouped into C1QA/B/C+ FOLR2+ CCR2^neg^ (clusters 1, 4–6, 9), C1QA/B/C^neg^ FOLR2^neg^ CCR2+ (clusters 2, 3, 10) and C1QA-C + FOLR2^low^ CCR2+ (cluster 8) clusters (Figure S15). Macrophages in cluster 8 were TREM2+ MRC1+ MERTK^low^ *CD48*^low^ and expressed *CLEC10A* gene (Figure S15).

The C1QA/B/C^neg^ CCR2+ macrophages comprised TREM2+ CD48^low^ SPP1+ (cluster 2), TREM2^neg^ CD48^high^ S100A12+ PLAC8+ SELL+ CD52^high^ (cluster 3) and TREM2^neg^ CD48^+^ CLEC10A+ (cluster 10) subsets (Figures 6A and S15). These cells were MERTK^neg^ MRC1^low/neg^ and CD163^low^. SPP1+ macrophages were reported as enriched in RA synovitis^18^ and bronchoalveolar lavage from patients with severe COVID.^32^ Furthermore, SPP1-expressing hepatic lipid-associated macrophages were shown to derive from bone marrow, replacing Kupffer cells (KCs) in metabolic-associated fatty liver disease.^33^ Genes linked to proinflammatory (*CD114*, *IL1B*, S100A8, S100A9, and *FCAR*) and host defense (*FCN1*, *PPI*F, and *CD93*) functions were highly expressed in cluster 3 macrophages (Figure S15).

The C1QA/B/C+ FOLR2+ CCR2-synovial macrophages segregated into FOLR2^high^ MERTK+ CD163^high^ SELENOP^high^ (clusters 5, 6, 9), proliferating FOLR2^low^ MERTK^low^ TOP2A+ CENPF+ (cluster 4) and cluster 1 subsets, containing FOLR2^low^ MERTK^low^ SPP1+ and FOLR2+ MERTK+ TIMD4+ macrophage populations (Figures 6A and S15). Macrophages in clusters 1, 4–6 and 9 were TREM2+; TREM2 has been linked to regenerative wound healing^34^ and pro-tumorigenic macrophage functions.^35^ They expressed the genes encoding *CMKLR1*, the receptor for resolvin E,^36^ and *SLCO2B1* transporter, associated with macrophage identity.^37^ In addition to being enriched for complement component-encoding transcripts (*C1QA, C1QB, C1QC*), these cells expressed genes encoding scavenger receptors (*CD206, STAB1*,^38^ and *CD163*), molecules involved in efferocytosis (*MERTK*, *LGMN*^39^) and cholesterol/lipid trafficking (*APOE*) (Figure S15), pointing to their tissue clearing functions. Looking closer at FOLR2^high^ MERTK+ CD163^high^ SELENOP^high^ macrophages (clusters 5, 6, 9), we identified COLEC12^high^ TIMD4+ macrophages (cluster 5), CD48^med^ macrophages (cluster 6) and COLEC12^high^ LYVE1+ macrophages (cluster 9) (Figures 6A and S15). The expression of *TIMD4* and *LYVE1* genes was associated with macrophage tissue residency^40^ and the perivascular location,^41^ respectively. A scavenger receptor collecting family member 12 (COLEC12)^42^ was shown to be involved in the clearance of microbes, oxLDL^43^ and myelin^44^ and acted as a high-affinity ligand for the collagen domain binding receptor LAIR1.^45^ Studies in mice showed that LAIR1 controls homeostatic and anti-tumor functions of monocytes and interstitial macrophages in the lungs via stromal sensing.^45^ COLEC12+ tissue-resident macrophages in clusters 5 and 9 were specifically enriched for *CCL13*, *CCL18*, and *NUPR1* (Figure S15), the regulators of lymphoid/myeloid cell trafficking^46^^,^^47^ and ferroptosis,^48^ respectively. Finally, LYVE1+ macrophages (cluster 9) expressed *CD209* while being enriched in transcripts associated with coagulation (*F13A1*^49^), tissue adaptation to stress (*IGF1*^50^), suppression of inflammatory genes (EGR1^51^) and iron recycling (*SLC40A1*^52^^,^^53^) (Figure S15).

Like other synovial cell types, the abundances of DC and macrophage subsets varied significantly across patient synovia (Figures 6B and 6C). Synovial tissues from our cohort contained on average more resident than inflammatory macrophages, while CD1C+ CLEC10A + DCs represented the most abundant subset of the synovial DC population (Figure 6C).

### Subcluster analysis uncovered endothelial cell diversity in freshly dissociated human synovium in inflammatory arthritis

**Figure 7:**
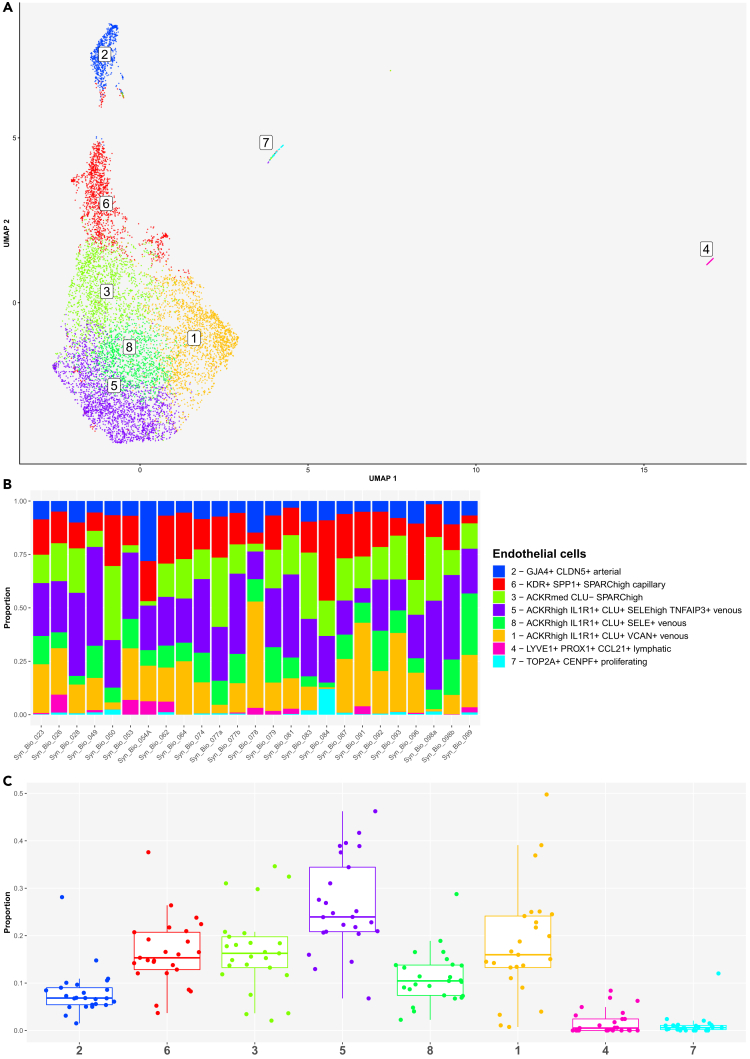
Subcluster analysis demonstrated heterogeneity of synovial endothelial cell (EC) population in the integrated synovial single-cell map dataset from 25 fresh-dissociated synovial biopsies of patients with inflammatory arthritis (A) UMAP of eight synovial EC clusters colored by EC cluster. (B) Bar plots of relative abundances of eight EC clusters across 25 synovial samples colored by EC cluster. (C) The proportion of the eight EC clusters across 25 synovial tissue samples from patients with inflammatory arthritis. The boxplot visualises five summary statistics: the median; two hinges, corresponding to the first and third quartiles; two whiskers. The upper (lower) whisker extends from the hinge to the largest (smallest) value no further than 1.5 ∗ inter-quartile range from the hinge. Individual dost represent data from different samples. See also Figures S8–S10, S16, and S17.

Our synovial scRNA-seq dataset contained 9395 PECAM+ synovial EC profiles forming eight EC clusters (Figure 7A). We identified pan-endothelial and subtype-specific EC markers^54^ and genes for the core EC functions (Figure S16). The eight EC clusters included LAPTM5+ PROX1+ LYVE1+ CCL21+ lymphatic ECs (cluster 4), proliferating TOP2A+ CENPF+ MKI67+ ECs (cluster 7), ACKR1+ venous ECs (clusters 1, 3, 5, 8), SPP1+ KDR+ capillary ECs (cluster 6) and GJA4+ CLDN5+ arterial ECs (cluster 2) (Figures 7A and S16). The EC clustering UMAP demonstrated the principal separation of synovial ECs into vascular and lymphatic clusters (dimension 1), while dimension 2 showed the core structure of tissue vasculature, starting with arterial, transiting into capillary and ending in venous vessel networks, as also confirmed with trajectory analysis (Figures 7A and S16A–S16C). Venous ACKR1+ ECs represented the largest synovial EC population (Figure 7A). Among them, we identified ACKR1^med^ VWF^med^ KDR^low^ SPARC^high^ CLU^neg^ ECs (cluster 3) sharing capillary and venous gene expression signatures (Figure S17), suggesting a transitional phenotype. The ACKR1^high^ VWF^high^ KDR^neg^ CLU+ ECs (clusters 1, 5, 8) expressed *IL1R1* transcripts (IL-1B response) and were enriched for *HLA-DR* and *HLA-DP* transcripts (antigen presentation). Clusters 5 and 8 were additionally IL6+ SOD2+ SOCS3^high^ and expressed *IRF1* (interferon response). *ICAM1* (leukocyte *trans*-endothelial migration) and *SELE* (neutrophil adhesion) were enriched in clusters 5 and 3 cells, while *CCL2* and *TNFAIP3* genes were highest expressed in cluster 5 venous ECs (Figure S17). SPP1+ KDR+ capillary ECs (cluster 6) were strongly enriched for transcripts encoding basement membrane-associated collagens (*COL4A1, COL4A2, COL15A1*)^55^ and plasmalemma vesicle associated protein (*PLVAP*) (Figure S17), a regulator of microvascular permeability and diaphragm formation within endothelial fenestrae.^56^ Finally, arterial ECs (cluster 2) were characterized by the expression of tight (*CLDN5*) and gap (*GJA4*) junction^57^^,^^58^ genes as well as genes involved in endothelial barrier regulation (*RHOB*),^59^ lymphatic vascular patterning (*SEMA3G*),^60^ regulation of TGFB activity (*LTBP4*), lymphocyte trafficking (*ICAM2*) and chemotaxis (*CXCL12*) (Figure S17). The trajectory analysis independently confirmed top arterial (*PODXL*, Figure S16A), capillary (*SPARC, COL4A1, COL4A2, COL15A1*, and *PLVAP*, Figure S16B) and venous (*VWF*, *ACKR1*, and *CLU*, Figure S16C) EC gene markers and affirmed that synovial venous ECs are main expressors of cell adhesion- (*SELE, SELP, ICAM1*, Figure S16D) and antigen presentation-linked transcripts (*HLA-DR, HLA-DP, CD74,* Figure S16E) among synovial ECs. Additionally, several signaling pathway related-genes were identified. *JUN*, *JUNB*, *FOS*, and *FOSB* (Figure S16F) - the genes encoding the AP-1 transcription factor components - were primarily expressed in arterial and venous ECs, *FLT1* mRNA was highest in capillary ECs, while *NFKIB*, *IRF1*, and *SOCS3* transcripts were enriched in venous ECs. The eight EC clusters were detectable in all patient samples, however their abundances varied significantly across patient synovia (Figures 7B and 7C). The ACKR1^high^ IL1R1+ CLU+ SELE^high^ TNFAIP3+ venous ECs represented on average the most abundant endothelial cell cluster, whereas LYVE1+ PROX1+ CCL21+ lymphatic and TOP2A+ CENPF+ proliferating ECs were scarcely represented in synovial biopsies from our patient cohort (Figure 7C).

### Merging main cluster and subcluster annotations derived a reference single-cell map of human synovium in inflammatory arthritis

**Figure 8:**
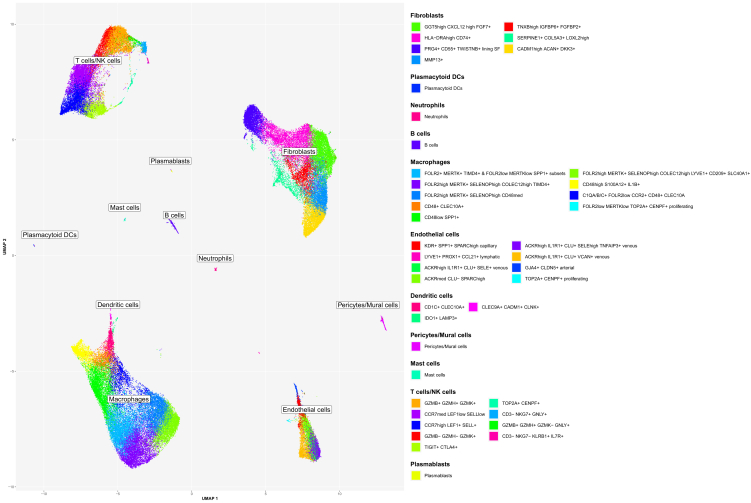
Single-cell reference map unveiled synovial cell composition in the fresh-dissociated human synovium in inflammatory arthritis A UMAP of annotated 12 lymphoid, 14 myeloid and 16 stromal cell synovial cell clusters and subclusters showing extensive cellular heterogeneity of the human synovium in inflammatory arthritis. Cluster annotations are based on the analysis of the main cell types and the subcluster analysis of synovial T-cells/natural killer (NK) cells/innate lymphoid cells (ILC), fibroblast, macrophage, dendritic cell (DC) and endothelial cell populations. The integrated scRNA-seq data are derived from 25 synovial tissue samples from patients with different types of inflammatory arthritis (see STAR Methods for details). See also Figures S8–S10.

In the next step, we merged the cell annotations from the main cell types (Figure S9A) and their subclusters (Figures 4, 5, 6, and 7) identified in the integrated scRNA-seq dataset from 25 freshly dissociated synovia of patients with inflammatory arthritis. These annotations represented a detailed scRNA-seq reference map of the fresh-dissociated human synovial cells in inflammatory arthritis (Figure 8).

### In house synovial scRNA-seq cell dataset served as a reference annotation resource to map synovial cell types in published synovial scRNA-seq datasets

**Figure 9:**
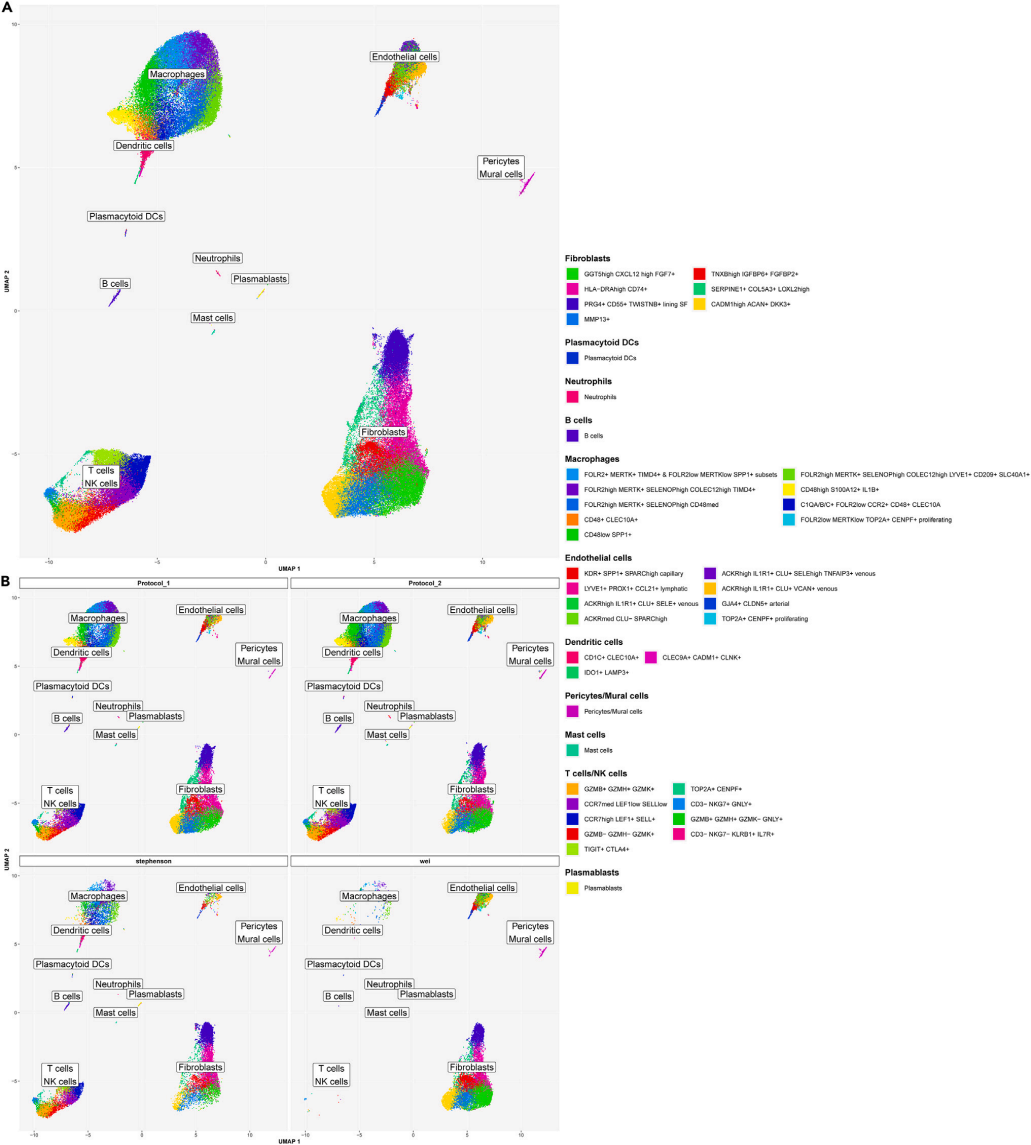
In-house synovial scRNA-seq reference dataset enabled the annotation of publicly available synovial scRNA-seq datasets Lower dimensional representation (UMAP) of our scRNA-seq dataset (“Protocol 1” and “Protocol 2”) integrated with two publicly available datasets (Stephenson W et al.^17^ and Wei K et al.^23^). The color represents the cell type as determined by either manual annotation (“Protocol 1” and “Protocol 2”) or label transfer (“Stephenson” and “Wei”) (see STAR Methods). Annotated are 12 lymphoid, 14 myeloid and 16 stromal cell synovial cell clusters and subclusters (see Figure 8 for details). (A) Integrated data from Wei K et al.,^23^ Stephenson W et al.^17^ and our studies combined in a single graph, (B) UMAPs with scRNA-seq data split by protocol and study. See also Figure S18.

As the final step in our analysis, we integrated our synovial scRNA-seq dataset (Figure 8) with publicly available synovial scRNA-seq data. We utilized our synovial cell map as a reference (Figure 8) to annotate cell types across the integrated dataset. We selected a dataset from Stephenson et al.^17^ generated from unsorted fresh dissociated synovial cells, alongside a dataset from Wei K et al.^23^ containing a large set of scRNA-seq synovial EC profiles, which were excluded from most other synovial scRNA-seq datasets. Using our synovial reference cell map, we successfully annotated the major synovial cell types and their subsets in the integrated (our, Stephenson,^17^ and Wei^23^) datasets (Figures 9A, 9B, and S18A). Stephenson’s dataset included all synovial cell types, including a minor synovial neutrophil population (Figures 9B, S18B, and S18C). Wei’s dataset comprised primarily synovial fibroblast, mural cells/pericytes and EC subsets (Figures 9B, S18B, and S18C), which were selectively sorted out from synovial tissue before scRNA-seq analysis. The abundance of main cell clusters varied significantly across patients in all datasets (Figures S18B and S18C).

## Discussion

Integrative single-cell omics is quickly expanding our understanding of unique and shared cell types across human tissues in health and disease.^61^ Fresh tissue dissociation facilitates less biased cell interrogation, while providing valuable insights into the biology and heterogeneity of cryopreservation-sensitive cell types like neutrophils. Single-cell transcriptomic analyses require the isolation of highly viable cells either from fresh or cryopreserved human tissues, the latter often necessitating viable cell sorting. Fluorescence-activated sorting of live cells before scRNA-seq is highly recommended for cells with compromised viability. Additionally, viability dyes are commonly included in cell sorting protocols that enrich specific cell populations, e.g., macrophages,^18^ or when cells are isolated from viably cryopreserved tissues.^62^

In this study, we aimed to build a single cell atlas of fresh unsorted human synovium in inflammatory arthritis. scRNA-seq analysis of unsorted cells relies strongly on the stable isolation of highly viable cells. We utilized the method of Donlin et al.^22^, optimized for the dissociation of cryopreserved synovia, as a starting reference protocol 1, and refined it for the dissociation of small fresh human synovial biopsies. We enhanced the release of synovial cells by gentle tissue massaging and minimized cell loss by optimizing the washing steps and volumes of reaction mixes. These protocol 2 refinements were associated with consistent isolation of good yield of viable synovial cells, thereby overcoming significant sample loss, associated with the original protocol 1. In our study, we did not sort cell suspensions for live cells using viability dyes. Sorting live cells can rescue cell suspensions with good cell quantity but impaired cell viability, however none of the excluded protocol 1 suspensions fulfilled these criteria. In contrast, the protocol 2 cell suspensions were highly viable, while varying substantially in cell yield. Considering that fluorescence-activated cell sorting leads to a certain degree of cell loss, sorting would have a limited or no advantage for scRNA studies of highly viable single cell suspensions from protocol 2.

Overall, we established a reliable dissociation method for fresh, prospectively collected synovial biopsies that can facilitate scRNA-seq and multiparameter spectral flow cytometry studies on highly viable unsorted synovial cells. This method complements the original protocol 1 by Donlin et al.,^22^ which is optimized for multicentric omics studies on cryopreserved synovia.

Neutrophils have been broadly implicated in the pathogenesis of inflammatory arthritis, particularly RA.^63^ Neutrophils primarily localize to inflamed synovial fluid, but are present also in synovial tissue, albeit at low frequency when compared to other cell types. In RA, neutrophils colocalized with fibrin deposits along the synovial lining; and fibrin and neutrophils were significantly associated with prolonged RA morning stiffness.^64^ Furthermore, neutrophil extracellular traps (NETs) contributed to cartilage damage and promoted the formation of autoantibodies to carbamylated^65^ and citrullinated^63^^,^^66^ proteins in RA. Nanoparticles coated with neutrophil membranes neutralized synovial inflammation and attenuated cartilage damage in experimental inflammatory arthritis.^67^

The short lifespan and intrinsically small library sizes have challenged detection of neutrophils using scRNA-seq methods, such as the droplet-based 10x Genomics platform.^68^^,^^69^ In our study, we detected a minor population of IFITM2^high^ synovial neutrophils in scRNA-seq data from freshly dissociated synovia, characterized by the expression of different maturation and canonical neutrophil marker genes (*NAMPT, SOD2, CSF3R, FCGR3B, GO2, S100A9,* and *S100A9*). A paucity of detected neutrophils in the scRNA-seq dataset was in line with histology analyses, where neutrophils were scarcely present. We detected neutrophils mainly in samples from protocol 2 donors, as evidenced by histology and scRNA-seq analyses. The overlap of both methods in neutrophil detection was 50%, which could be attributed to neutrophil scarcity and their focal distribution within and across biopsy fragments. Overall, neutrophil detection was primarily donor-dependent and not protocol-specific (Figure S9B). Furthermore, sequencing of a high number of total non-sorted synovial cells and dissociating synovium fresh could facilitate neutrophil detection in our study irrespective of the protocol used. Our integrative analysis of in-house scRNA-seq and publicly available human synovial scRNA-seq datasets^17^^,^^23^ included data from 20, 000 fresh dissociated unsorted synovial cells from Stephenson et al.^17^ We identified a minor neutrophil population also in Stephenson’s data. No neutrophils were detected in Wei’s data,^23^ created on sorted CD45^−^ CD235-synovial stromal cells from cryopreserved synovial tissues. Likewise, many other published synovial scRNA-seq datasets were created on pre-sorted synovial cells, excluding neutrophils.^16^^,^^18^^,^^23^

Our study reproduced different previously described synovial leukocyte and structural cell subsets,^16^^,^^17^^,^^18^^,^^23^ while expanding transcriptional heterogeneity of synovial cell states and detecting synovial neutrophils. We identified a small population of SDC4+ synovial fibroblasts and SERPINE1+ COL5A3+ synovial fibroblasts, and broadened the transcriptional characterization of tissue-resident COLEC12^high^ synovial macrophages. SDC4 has been closely linked to synovial fibroblast attachment/invasion into articular cartilage and cartilage breakdown,^29^^,^^70^ while COLEC12 and SLC40A1 were associated with macrophage extracellular matrix sensing^45^ and regulation of iron homeostasis.^52^^,^^53^ We observed a minor subset of C3^high^ CXCL14^high^ cells within the sublining GGT5^high^ CXCL12^high^ synovial fibroblast cluster. Notably, synovial fibroblasts were recently shown to drive the local inflammatory tissue priming in preclinical models of arthritis in a C3-dependent manner; the primed fibroblast appeared to upregulate both *C3* and *CXCL14* mRNA expression.^71^ Future phenotypic and functional experiments should confirm whether gene signatures and annotated cell clusters also translate into specific synovial cell functions.

We characterized in depth the heterogeneity of synovial vascular and lymphatic EC populations. Venous cells, which were the most abundant EC subset in inflamed synovium, demonstrated an enhanced expression of genes encoding cell adhesion molecules and components of antigen presentation apparatus. This data suggested that not only synovial fibroblasts,^16^ but also venous ECs might serve as antigen-presenting cells in the inflamed human synovium. Additionally, different signaling pathways appeared distinctly enriched across synovial EC populations. JUN and FOS proteins form a dimeric transcription factor AP-1, which is known to be enriched in the enhancers of EC-specific genes.^72^ In our data, JUN and FOS transcripts were increased in arterial and venous ECs compared to capillary ECs. In turn, capillary ECs appeared principal expressor of FLT1, and venous ECs of IRF1 and NFKIB genes. These results deduced a possible role of capillary ECs in synovial angiogenesis, while reinforcing the probable pro-inflammatory function of synovial venous ECs.

Differential enrichment of distinct cell types across patient synovia might drive different pathogenic pathways and contribute to the characteristic interpatient variability in therapeutic responses to currently prescribed disease-modifying antirheumatic drugs.^30^ Our in-depth analysis of synovial EC diversity in inflammatory arthritis demonstrated the shared contribution of GGT5+ synovial fibroblasts and ACKR1^high^ SELE ^high^ TNFAIP3+ venous EC subsets to the synovial production of IL-6. The variable abundances of IL6-producing cell subsets across patient synovia may shape the differential therapeutic responses of arthritis patients to anti-IL6 therapies or JAK inhibitors. Future studies involving larger stratified patient cohorts will be essential to link synovial cell composition to variation in therapeutic response. Furthermore, such studies might contribute to the cell subtype-based and molecular pathway-based patient stratification across and within different clinical types of inflammatory arthritis.^62^

In conclusion, we refined the synovial dissociation protocol for prospectively collected fresh synovial biopsies and generated an extensive reference single-cell resource of freshly dissociated human synovium in inflammatory arthritis. The refined synovium dissociation protocol could facilitate future prospective single-cell omics studies on human synovium. Meanwhile, our reference scRNA-seq synovial atlas diversifies the knowledge about synovial cell composition in inflammatory arthritis, by identifying diverse synovial cell states, characterizing synovial neutrophil transcriptomes and offering detailed insights into synovial endothelial cell heterogeneity.

### Limitations of the study

Data on arthritis progression at biopsy were not available for this study. Consecutively recruited patients formed clinically and therapeutically heterogeneous patient groups, precluding a deeper association of clinical features with scRNA-seq synovial cell profiles. Additionally, comparing synovial cell subset enrichment across different types of arthritis could not be performed due to a limited number of samples from patients with ankylosing spondylitis, psoriatic arthritis and undifferentiated arthritis. Inflammatory arthritis associates with sex, and sex might influence arthritis pathobiology. Protocol 1 scRNA-seq data were generated from synovia of female patients only, while protocol 2 scRNA-seq cohort included female and male subjects. To address potential sex effects on synovial cell composition, we conducted a differential abundance analysis on protocol 2 scRNA-seq samples. This analysis showed no effect of sex on synovial cell type abundance. However, a larger patient cohort is needed to confidently address the influence of sex on synovial cell type abundances. Using scRNA-seq, we identified 42 transcriptionally distinct synovial cell clusters, which formed 11 major synovial cell populations. These cell populations and their core subpopulations were detected also with flow cytometry. To explore functional/activity states of all 46 transcriptomically distinct cell subsets, detailed immunophenotyping analysis (e.g., using spectral flow cytometry, CyTOF or CITE-seq) and functional studies are required. Given synovial neutrophil scarcity, spatial omics on synovial biopsies might valuably complement neutrophil scRNA-seq studies, while big tissue fragments/large patient cohorts would be required to sort sufficient synovial neutrophil quantities for in depth studies of synovial neutrophil diversity and biology. Additionally, spatial omics could support the cell-cell communication inference analyses on scRNA-seq data, the output of which varies considerably depending on the method and resources used. We did not conduct cell-cell communication inference analyses of synovial scRNA-seq data in this study; these analyses should accompany future spatial omics studies on human synovial tissue in inflammatory arthritis.

## STAR★Methods

### Key resources table

**Table:** 

REAGENT or RESOURCE	SOURCE	IDENTIFIER
**Antibodies**		

Monoclonal mouse anti-human CD31/PECAM-1, Clone JC/70a	DAKO, Agilent	Cat# GA61051-2
Mouse anti-CD20, Clone L26	Ventana Roche	Cat# 760-2531
Monoclonal mouse anti-human CD3, Clone LN10	Leica Biosystems	Cat# CD3-565-L-CE; RRID: AB_563541
Monoclonal mouse anti-human CD68, Clone PG-M1	DAKO, Agilent	Cat# GA61361-2
Monoclonal mouse anti-human CD138, Clone MI15	DAKO, Agilent	Cat# GA64261
Monoclonal mouse anti-Human CD15, Clone Carb-3	DAKO, Agilent	Cat# GA06261-2
APC anti-human CD45	BioLegend	Cat# 982304; RRID: AB_2650648
PE anti-human CD64	BioLegend	Cat# 399504; RRID: AB_2861005
BV421™ anti-human CD11b	BioLegend	Cat# 393114; RRID: AB_2750257
Alexa Fluor® 700 anti-human CD3	BioLegend	Cat# 317340; RRID: AB_2563408
BV785™ anti-human CD19	BioLegend	Cat# 363028; RRID: AB_2564257
anti-Podoplanin-BB700	BD Biosciences	BD OptiBuild Cat# 749715; RRID: AB_2873969
anti-CD31-BUV395	BD Biosciences	BD Horizon Cat# 565290; RRID: AB_2739159
anti-CD45-PE/Fire810	Sony Biotechnology	Cat# 1116435
anti-CD14-cFluorB548	Cytek Biosciences	Cat# R7-20115
anti-CD3-BV605	BD Biosciences	BD OptiBuild Cat# 742623; RRID: AB_2740919
anti-CD8b-PE	BioLegend	Cat# 387307; RRID: AB_3068193
anti-CD4-PE-Cy7	BioLegend	Cat# 317413; RRID: AB_571958
anti-CD19-BB515	BD Biosciences	BD Horizon Cat# 564457; RRID: AB_2744309

**Biological samples**		

Human synovial tissue biopsy	University Hospital Zürich, Zurich, Switzerland	N/A
Human synovial tissue biopsy	Hospital Santa Maria, CAML, Lisbon, Portugal	N/A

**Chemicals, peptides, and recombinant proteins**		

Liberase™ TL Research Grade	Roche	Cat# 05401020001
DNase I – from bovine pancreas	Roche	Cat# 11284932001
Red Blood Cell Lysis Solution (10x)	Miltenyi Biotec	Cat# 130-094-183
UltraPure™ BSA (50 mg/ml)	Thermo Fisher Scientific	Cat# AM2616
Acridine Orange PI	BioCat	Cat# F23002-LG
Cell Staining Buffer	BioLegend	Cat# 420201
Human TruStain FcX™	BioLegend	Cat# 422301
Zombie Green™ Fixable Viability Dye	BioLegend	Cat# 423111
BD™ CompBeads Anti-Mouse Igκ/Negative Control Compensation Particle Set	BD Biosciences	Cat# 552843
autoMACS® Rinsing Solution	Miltenyi Biotec	Cat# 130-091-222
MACS BSA Stock Solution	Miltenyi Biotec	Cat# 130-091-376
BD Horizon™ Brilliant Stain Buffer	BD Horizon	Cat# 563794
FcR Blocking Reagent, human	Miltenyi Biotec	Cat# 130-059-901
UltraComp eBeads™ Compensation Beads	Invitrogen	Cat# 01-2222-42

**Critical commercial assays**		

Chromium Single Cell 3’ GEM, Library & Gel Bead Kit v3	10x Genomics	PN-1000075
Chromium Single Cell B Chip Kit	10x Genomics	PN-1000073
Chromium i7 Multiplex Kit	10x Genomics	PN-120262
Chromium Next GEM Single Cell 3’ GEM, Library & Gel bead kit v3.1	10x Genomics	PN-1000121
Chromium Next GEM Chip G Single Cell Kit	10x Genomics	PN-1000120
Single Index Kit T Set A	10x Genomics	PN-1000213

**Deposited data**		

scRNA-seq data Edalat G. S. et al.	This paper ArrayExpress	ArrayExpress: E-MTAB-11791 Samples 28, 50 data also Accession #: GSE181082
Published scRNA-Seq datasets	Stephenson et al.^17^, dbGaP	dbGap: phs001529.v1.p1
Published scRNA-Seq datasets	Wei et al.^23^	https://portals.broadinstitute.org/single_cell/study/SCP469
Original code and instructions	This paper	https://retogerber.pages.uzh.ch/protocol_synovial https://retogerber.pages.uzh.ch/synovialscrnaseq

**Software and algorithms**		

FlowJo v10.10.0	BD Biosciences	https://www.bdbiosciences.com/en-us/products/software/flowjo-v10-software
FACSDiva v9.0.1	BD Biosciences	https://www.bdbiosciences.com/en-us/products/software/instrument-software/bd-facsdiva-software
SonyID7000™ V2.0.2	Sony Biotechnology	https://www.sonybiotechnology.com/us/instruments/id7000-spectral-cell-analyzer/software/
GraphPad Prism v10.1.2	GraphPad, Dotmatics	https://www.graphpad.com/updates/prism-10-1-2-release-notes
R v4.0.3	R Core Team	https://cran.r-project.org/bin/windows/base/old/4.0.3/
Bioconductor v3.12	Bioconductor	https://bioconductor.org
Cell Ranger v6.0.0	10X Genomics	https://www.10xgenomics.com/support/software/cell-ranger/latest/release-notes/cr-release-notes

**Other**		

ART™ wide bore filtered pipette tips	Thermo Fisher Scientific	Cat# 2069G
Blunt cannula 80 mm, 16 G, Ø 1.6 mm	APPLIMED SA	Cat# 8001741
BD Plastipak™ Syringe	BD	Cat# 303172
Corning® 40μm Cell Strainer	Corning	Cat# 431750
Corning® 70μm Cell Strainer	Corning	Cat# 431751

### Resource availability

#### Lead contact

Further information and requests for resources and reagents should be directed to and will be fulfilled by the lead contact: Mojca Frank Bertoncelj (frankbertoncelj@bio.mx).

#### Materials availability

This study did not generate new unique reagents.

#### Data and code availability

• The complete scRNA-seq dataset from 25 synovial tissue samples of patients with inflammatory arthritis have been deposited at the ArrayExpress: E-MTAB-11791, https://www.ebi.ac.uk/arrayexpress/ and are publicly available as of date of publication. The Accession number is listed in the key resources table. The data from patients 28 and 50 have been deposited in the NCBI Gene Expression Omnibus as part of another publication^73^ and are accessible also through GEO SuperSeries: GSE181082. The accession numbers for these datasets are listed in the key resources table.
• The original code and instructions for the integrative analysis of scRNA-seq data generated by protocols 1 and 2 are available at https://retogerber.pages.uzh.ch/protocol_synovial. The original code and instructions for the synovial single-cell reference map analysis are available at https://retogerber.pages.uzh.ch/synovialscrnaseq. Links are listed in the key resources table.
• Any additional information required to reanalyse the data reported in this paper is available from the lead contact upon request.

### Experimental model and study participant details

#### Study participants

We enrolled 40 consecutive patients with different types of inflammatory arthritis, who were admitted to the Department of Rheumatology, University Hospital Zurich, Switzerland, for synovial biopsy and introduction/change of arthritis therapy. The arthritis diagnosis was set at the time of admission and was based on classification criteria for different arthritis conditions^2^^,^^74^^,^^75^ and clinical examination by a rheumatologist. Diagnosis of undifferentiated arthritis (UA) diagnosis was set when arthritis did not meet the criteria for any defined arthritis type. In total, we collected 42 synovial tissues from 19 patients with RA, 6 patients with spondylarthritis, 6 patients with psoriatic arthritis and 9 patients with UA. Two RA patients donated biopsies from two different joints. These paired biopsies were processed as independent samples for downstream analyses. Synovial tissue samples originated from different joints, including knees (n=17), wrists (n=13), metacarpophalangeal joints (n=10) and sternoclavicular joints (n=2). Twenty five biopsies from 23 patients were utilized for single cell RNA-sequencing studies; the median age of patients was 55.5 years (range 20 – 81 range), 78% of patients were of female sex (Tables 1 and 2). For proof-of-principle flow cytometry experiments, we additionally collected synovial biopsy samples from two arthritis patients at Hospital de Santa Maria, Lisbon Academic Medical Center (CAML), Lisbon, Portugal. Biopsy samples were obtained from the wrist of a 46 year-old male patient with early RA, and the knee of a 66 year-old female patient undergoing diagnostic synovial biopsy and synovial fluid analysis (Table S2). Synovial fluid analysis retrospectively confirmed the diagnosis of septic arthritis caused by *Neisseria gonorrhoeae.* Data on ancestry, race, ethnicity, and socioeconomic information of study subjects were not available for our study. The ethics committee of Canton Zurich, Switzerland (Swissethics Number: 2019-00674, PB-2016-02014 and 2019-00115), and CAML Ethics Committee, Lisbon, Portugal (Reference N. 110/22) approved the collection and analysis of patient data and synovial tissues. All patients signed the informed consent for participation in the study before inclusion.

#### Study workflow

All 44 synovial biopsies used in this study were processed fresh, and the presence of synovium was confirmed histologically for all included samples. Among the 42 dissociated synovial tissue samples in the University Hospital Zurich cohort, six samples were utilized for other downstream applications while eight samples were discarded because of the compromised cell viability/yield. Among 28 sequenced samples, we excluded three samples because of the limited quality of the scRNA-seq library/data. The final synovial scRNA-seq dataset included single cell profiles from 25 high-quality synovial cell suspensions obtained from patients with RA (n=15 biopsies), psoriatic arthritis (n=3 biopsies), spondylarthritis (n=4 biopsies) and UA (n=3 biopsies), dissociated either with protocol 1^22^ or protocol 2. In integrative protocol analysis, we integrated scRNA-seq data from RA and psoriatic arthritis synovial tissue samples, which were evenly distributed between the two protocols. All spondylarthritis synovia were dissociated with protocol 1 and all UA synovia with protocol 2. To create a reference single-cell map of the fresh human synovium in inflammatory arthritis, we integrated the scRNA-seq data from all 25 synovial tissue samples. Two synovial biopsies in the CAML cohort were dissociated fresh using protocol 2 at the Instituto de Medicina Molecular João Lobo Antunes, Lisbon, Portugal, and total unsorted synovial cells were analyzed using multicolor (n=1, biopsy from a patient with septic arthritis) and spectral flow cytometry (n=1, biopsy from early RA patient).

### Method details

#### Ultrasound-guided synovial tissue biopsy

We obtained synovial tissue by ultrasound-guided synovial biopsy using a Quick core 18G x 6cm or a Quick Core 16G 10cm semi-automatic, guillotine-type biopsy needles and a General Electric® Logiq S7 ultrasound machine (GE Healthcare, Boston, Massachusetts, USA).^76^^,^^77^ Joints were evaluated for synovitis, before biopsy, using a GE Logiq S7 ultrasound machine equipped with a broad-spectrum linear array hockey-stick L8-18i transducer and a broad-spectrum linear matrix array ML6-15 transducer or a Canon® Aplio i800 ultrasound machine (Canon Medical Systems Corporation, Tochigi, Japan), with a multi-frequency ultra-wideband linear i18LX5 transducer and a 22 MHz ultra-high frequency hockey stick i22LH8 transducer. All joints were scanned according to the EULAR Ultrasound Scanning Guide.^78^ Ultrasound synovitis scoring (grayscale synovial hypertrophy, PD ultrasound parameters, semiquantitative grading 0-3) was performed under the EULAR-OMERACT guidelines.^79^ Synovial tissue biopsy fragments were utilized for immunohistology, optimization of tissue dissociation protocol, scRNA-seq analysis, multicolour flow cytometry, spectral flow cytometry analysis or other downstream applications. Multiple synovial tissue fragments were collected from each donor. Specifically, for histological analysis, we scored a median of 7 (4-15) tissue fragments per joint, collected from different synovial tissue sites within each joint. For scRNA-seq analyses, we pooled a median of 11 (6-20) biopsy tissue fragments from the same joint to mitigate the known fragment-to-fragment variability and facilitate representative capture of the synovitis process in individual joints.

#### Histological analysis of synovial tissue

To identify the presence of synovial tissue in biopsied tissue fragments and to grade synovitis, biopsy fragments were formalin-fixed, paraffin-embedded, and sectioned (2μm) followed by haematoxylin-eosin, Giemsa and Elastica van Gieson staining. The identity of synovial tissue in biopsied fragments was confirmed based on the presence of a synovial lining layer and histologic features of the synovial sublining layer (fibrovascular or fatty tissue). For each tissue sample, grading of synovitis was performed based on the Krenn synovitis scoring (0-9) and its sub-scores evaluating synovial lining hyperplasia (0–3), stromal cell activation (0-3) and immune cell infiltration (0–3).^26^ For pathotype analysis,^14^ slides were deparaffinized and automatically stained with specific antibodies against synovial cell markers (PECAM-1, DAKO JC/70a, 1:10; CD20, Ventana Roche L26, prediluted; CD3, Leica LN10, 1:500; CD68, DAKO A/S PG-M1, 1:50; CD138, DAKO A/S, MI15, 1:30 and CD15, DAKO A/S Carb-3, 1:100). We used the HRP-based DAB staining for antigen visualization and performed pre-treatments according to the manufacturer's instructions (Ventana BenchMark). Examination of histological specimens was conducted at the Department of Pathology, University Hospital Zurich, Switzerland by a pathologist and a rheumatologist and at the Instituto de Medicina Molecular, Lisbon, Portugal by a rheumatologist.

#### Cell isolation from fresh synovial biopsies

Synovial biopsies used for protocol optimization, scRNA-seq, multicolour flow cytometry and spectral flow cytometry were submerged in sterile RPMI culture medium, transferred to the laboratory, and processed within 1-2 hours of collection. Forty-four fresh synovial biopsy samples were dissociated either with the protocol of Donlin et al.^22^ (protocol 1, n=26), or our optimized protocol (protocol 2, n=18) derived from Donlin et al.^22^ Briefly, synovial biopsies were minced and digested in a mixed enzymatic-mechanical dissociation including 100 μg/ml Liberase TL (Roche) and 100 μg/ml DNAse I (Roche); red blood cells were lysed (Red Blood Lysis buffer, Roche), and synovial cell pellets washed. For a detailed description of our protocol (protocol 2) and reagents used see step-by-step synovial tissue dissociation protocol 2 section and key resources table. Cell counts and viability were determined using either manual (Neubauer, Trypan Blue) or automated (Luna-FL, Dual Fluorescence Cell Counter, Logos BioSystems, Acridine Orange/PI, ThermoFisher) cell counting. Neubauer chamber and Luna Dual Fluorescence Cell Counter enable visualization of single cells and cell clusters, guiding the required number of filtering steps to facilitate loading of single cell suspensions onto 10x Genomics chips.

#### Step-by-step synovial tissue dissociation protocol 2

##### Step 1 Digest synovial tissue

In the first step of protocol 2, fresh synovial tissue is washed to remove the potential sources of non-synovial cell contamination, minced into small fragments, and dissociated into synovial cell suspension using the combined enzymatic-mechanical tissue dissociation protocol. **CRITICAL**: Volumes of digestion mixes are refined to minimize cell loss.

###### Timing: 47 min

1. Wash synovial biopsy fragments in D-PBS.
  a. Transfer the biopsied tissue fragments with sterile forceps onto a wet membrane of a 70 μm cell strainer sitting in a well of a 6-well plate, filled with 4 ml PBS.
  b. Transfer the strainer with tissue to neighboring wells with fresh 4 ml D-PBS. Repeat this washing step 2x. Keep the tissue wet. Washing removes potential cell contaminants that do not originate from tissue.
2. Mince synovial tissue.
  a. Transfer tissue fragments into a 250 μl pre-warmed RPMI (glutamine, HEPES, no Abs, no FBS) medium droplet on a round bottom culture plate.
  b. Mince synovial tissue with a sterile scalpel into small tissue fragments (∼1 mm). Keep the tissue wet. Prepare digestion mix.
  c. Transfer 500 μl of the pre-warmed RPMI medium (glutamine, HEPES, no Abs, no FBS) into a conical 15 ml polystyrene tube. Add 40 μl of 2.5 mg/ml Liberase TL, Roche to reach 100 μg/ml working Liberase TL concentration and 10 μl of 10 mg/ml DNAse I, Roche to get 100 μg/ml working DNase I concentration.
  d. Transfer the minced tissue fragments with a wide-bore pipette tip, forceps or scalpel into the digestion mix and top the volume with RPMI (glutamine, HEPES, no Abs, no FBS) to a total of 1 ml. Collect all tissue fragments, which might stick to the tip, scalpel, or forceps surfaces. Avoid using forceps with serrated surfaces to prevent excessive tissue sticking and tissue loss.
  e. Add magnetic stirrer(s) in the tube and close the tube safely.

3. Digest synovial tissue at 37°C in a mixed enzymatic-mechanical protocol.
  a. Transfer the tube with tissue fragments into a 37°C water bath on a magnetic holder, placed within the prewarmed oven (37°C).
    **NOTE**: The water bath facilitates keeping the suspension temperature at 37°C during tissue digestion.
  b. Digest tissue for 30 min at 37°C with a continuous magnetic stirring.
    **NOTE:** A combination of a ball-shaped and a cylinder-shaped stirrer facilitates tissue mixing during digestion.
  c. Fifteen minutes after starting the digestion, pass tissues gently through a 16G needle (10x) using a 1 ml syringe (mechanical dissociation).
    **CRITICAL**: The 16G needle may clog with tissue fragments; the built pressure may detach the needle, leading to tissue fragment/cell loss. The 1 ml syringe fits in the falcon tube, preventing the loss of cell suspension in case of needle detachment.

4. Stop enzymatic digestion.
  a. Add 200 μl of FBS and 800 μl of RPMI (glutamine, HEPES, no Abs, no FBS) to the digestion mix to reach the final 10% FCS concentration.

##### Step 2 Enrich dissociated synovial cells

In this step, tissue debris and cell aggregates are removed to obtain a single-cell suspension of synovial cells.

**CRITICAL**: Synovial cell suspension is enriched by enhancing the release of synovial cells from the digested tissue. Additional washes are introduced to minimize cell loss.

###### Timing: 16 min

1. Pre-wet the 40 μm strainer with 2 ml of RPMI medium (glutamine, HEPES, no Abs, 10% FBS) in the six-well plate. Filter the cell suspension through the 40 μm strainer submerged into the medium using 1 ml wide-bore pipette tips.
2. Wash the falcon tube with an additional 1 ml of RPMI containing 10% FCS to collect the remaining cells and filter through the pre-wet 40 μm strainer into the well with filtered cell suspension.
3. Tissue debris and poorly disaggregated cell clumps will remain on the strainer. Using the syringe plunger head, gently press the synovial tissue fragments against the strainer to facilitate further cell release.

**NOTE:** Collect any remaining drops of cell suspension from the bottom side of the strainer with a fresh pipette tip and add to the filtered cell suspension.

**CRITICAL:** This step releases additional cells from the tissue.

4. Transfer the 5 ml of the filtered cell suspension into a 15 ml Falcon tube.
5. Wash the well with an additional 5 ml of RPMI containing 10% FCS to collect the remaining cells.
6. Centrifuge the enriched filtered cell suspension at 300 x g, RT, 10 min.

##### Step 3 Lyse red blood cells

In this step, red blood cells are lysed, and synovial cells are washed.

###### Timing: 27 min

1. Lyse the red blood cells.
  a. During centrifugation, prepare 1x red cell lysis buffer (Roche) by mixing 0.5 ml of 10x RBC Lysis Buffer in 4.5 ml of nuclease-free H2O.
  b. Remove the supernatant and resuspend the pellet gently in 0.5 ml RPMI containing 10% FCS.
  c. Add 5 ml of 1x RBC lysis buffer to the cells in 10% RPMI, vortex gently 5 sec, incubate 2 min RT.
  d. Centrifuge cell suspension at 300 x g, RT, 10min. Remove the supernatant.
2. Wash the cells in D-PBS.
  a. Resuspend cells gently by flicking the pellet and then wash the cells with 10 ml PBS. Centrifuge at 300 x g RT, 10 min.
  b. Remove the supernatant.
  c. Centrifuge 2 min at 300 x g, RT to remove any residual solution.

##### Step 4 Prepare single cell suspension for scRNA-seq

In this step, the enriched cells are counted and checked for viability, potential remaining cell debris and aggregates are removed by additional filtering steps, and the suspension of single synovial cells is prepared for downstream applications (e.g., scRNA-seq).

###### Timing: 10 min

1. Prepare fresh 0.2% BSA D-PBS (4 μl of 50 mg/ml BSA per 100 μl D-PBS).
2. Gently flick the pellet and resuspend the cells with wide-bore tips in 100-150 μl in 0.2% BSA D-PBS. Keep the cells on ice.
3. Count the cells, determine cell viability and check for debris or cell clumps (e.g., with Luna-FL dual fluorescence cell counter, Logos BioSystems). If remaining debris or cell clumps, filter the cell suspension through the 35 μm strainer into a 1.5 ml Eppendorf tube.

**CRITICAL:** This step helps to prevent the clogging of the 10xGenomics chips.

4. Adjust the cell number to 700 single synovial cells/μl. Dilute or concentrate the cell suspension in 0.2% BSA D-PBS accordingly.

**NOTE:** 700 cells/μl in our experience minimizes the probability of chip clogging.

5. Keep the cells on ice until starting with the 10x Genomics protocol.

#### Single-cell RNA-seq library preparation and sequencing

Synovial single-cell libraries were prepared using the 3’ v3.0 and 3’ v3.1 protocols and reagents from 10X Genomics according to the manufacturer’s instructions targeting the encapsulation of up to 6000 cells. We created two independent paired libraries from samples 23, 26 and 28 as part of the quality control pipeline. The quality and quantity of cDNA and libraries were assessed with Agilent Bioanalyzer (Agilent Technologies). Diluted 10nM libraries were pooled in equimolar ratios, and the library pools were sequenced on the Illumina NovaSeq6000 sequencer (paired-end reads, R1=28, i7=8, R2=91, min. 40,000 - 50,000 reads/cell) at Functional Genomics Center Zurich, the University of Zurich and ETH Zurich, Switzerland. A total of 6 separate sequencing runs on pooled scRNA-seq libraries, representing sequencing projects, were performed, as shown in table below. Two library pools (Below table) combined synovial and skin scRNA-seq samples, the latter being part of the skin protocol optimization and reference skin mapping project.^24^

**Table:** Per sample percentage of reads discarded because of swapped barcodes together with the sequencing project identity number (ID)

Sample	Proportion of reads discarded	Sequencing project ID
SB_023	NA	o20549, o20550
SB_026	NA	o20549, o20550, o23841
SB_028	NA	o21475
SB_049	NA	o21475
SB_050	NA	o21475
SB_053	NA	o23841
SB_054A	NA	o23841
SB_062	NA	o23841
SB_064	NA	o23841
SB_074	NA	o23841
SB_077a	NA	o23841
SB_077b	NA	o23841
SB_078	8.32%	o24300
SB_079	3.00%	o24300
SB_081	3.36%	o24300
SB_083	3.09%	o24300
SB_084	3.07%	o24300
SB_087	5.54%	o24300
SB_091	1.11%	o24793
SB_092	0.99%	o24793
SB_093	1.20%	o24793
SB_096	1.14%	o24793
SB_098a	1.20%	o24793
SB_098b	1.40%	o24793
SB_099	1.03%	o24793

Removal of swapped barcodes was performed for library pools o24300 and o24793, which contained scRNA-seq libraries from skin and synovium. Skin samples are part of another project^24^ and are not listed.

#### Multicolour flow cytometry

Fresh dissociated synovial cells were washed in PBS and pelted at 300 x g for 10 minutes. Cell pellets were resuspended in 100 μl Cell Staining Buffer, BioLegend, containing 5 μl Human TruStain FcX™ (BioLegend) and blocked for 10 min RT. After blocking, cells were labeled with APC anti-human CD45 (BioLegend), PE anti-human CD64 (BioLegend, Cat. No. 399504), BV421™ anti-human CD11b (BioLegend, Cat. No. 393114), Alexa Fluor® 700 anti-human CD3 (BioLegend, Cat. No. 317340), BV785™ anti-human CD19 (BioLegend, Cat. No. 363028) and 1 μl (1:100) of Zombie Green™ Fixable Viability Dye (BioLegend, Cat. No. 423111) at 4°C for 30 min. Cells were then washed, resuspended in D-PBS and analyzed with the BD FACSAria™ Fusion Flow Cytometer at the Flow Cytometry Facility of the Instituto de Medicina Molecular João Lobo Antunes. Compensation was performed using BD™ CompBeads Anti-Mouse Ig labeled with single antibodies and κ/Negative Control Compensation Particle Set (BD Biosciences, Cat. No. 552843) as recommended by the manufacturer. Data were analyzed using FACSDiva 9.0 and FlowJo V10.10.0.

#### Spectral flow cytometry

Fresh dissociated synovial cells (using protocol 2) were fixed in 2.5% PFA and washed in PBS. Fixed cells were shipped at 4 degrees from the Instituto de Medicina Molecular João Lobo Antunes, Lisbon, Portugal to BioMed X Institute, Heidelberg, Germany for spectral flow cytometry analysis. Cells were washed with FACS staining buffer (autoMACS® Rinsing Solution (Miltenyi Biotec #130-091-222) + MACS BSA Stock Solution (Miltenyi Biotec #130-091-376). The washed cells were blocked for 15 min at 4 degrees in FACS buffer containing BD Horizon™ Brilliant Stain Buffer (BDHorizon #563794) and FcR Blocking Reagent, human (Miltenyi Biotec #130-059-901). After blocking, cells were labeled with an antibody cocktail for 1h at 4 degrees, followed by cell washing and analysis on SonyID7000™ Spectral Cell Analyzer at BioMed X Institute, Heidelberg, Germany. The antibody cocktail included anti-Podoplanin-BB700 (BD OptiBuild #749715), anti-CD31-BUV395 (BDHorizon #565290), anti-CD45-PE/Fire810 (SONY #1116435), anti-CD14-cFluorB548 (Cytek #R7-20115), anti-CD3-BV605 (BD OptiBuild #742623), anti-CD8b-PE (BioLegend #387307), anti-CD4-PE-Cy7 (BioLegend # 317413) and anti-CD19-BB515 (BDHorizon #564457) antibodies. We used Sony ID7000™ software (Version 2.0.2) for sample unmixing, autofluorescence compensation and data analysis. The unmixing matrix was calculated using single antibody-labeled Ultracomp eBeads™ Kompensations-Beads (Invitrogen 01-2222-42).

### Quantification and statistical analysis

#### Statistical analysis

Statistical analysis was performed in GraphPad Prism Software (v10.1.2, GraphPad, Dotmatics). Data on viability and quantity of isolated cells, age, sex, Krenn synovitis score and 10xGenomics Chemistries for tissues dissociated with protocol 1 and protocol 2 were non-normally distributed. The statistical significance of a difference between the protocol 1 versus protocol 2 data was analyzed using the two-sided Fisher’s exact test (contingency table analysis of sex differences) or two-sided Mann-Whitney t-test (continuous variables); p< 0.05 accepted as a statistically significant difference. The statistical significance of the difference in sample drop-out between protocols 1 and 2 was calculated using Chi-square test with Yates’ correction. The statistical parameters (number of biological or technical replicates, median, ratio) are provided in the text of the Results section, the Figure legend text or the Table legend text.

#### Bioinformatics analysis of scRNA-seq data

Transcript count tables were generated from fastq files using cellranger count {cellranger} (version 6.0.0) and the reference Genome GRCh38.p13 (transcripts from GENCODE Release 32^80^ with genes coding for mitochondrial rRNA and tRNA, protein-coding RNA, rRNA and tRNA. Samples 23, 26 and 28 were each prepared as two independent libraries, sequenced in separate batches, and one of the sample 26 libraries was subsequently re-sequenced to reach the comparable sequencing depth across all libraries. Independent samples 23, 26 and 28 libraries were combined with cellranger aggr. Additionally, the re-sequenced library from sample 26 was combined with the corresponding sample data. Pooling a subset of synovial libraries with skin scRNA-seq^24^ libraries led to a limited barcode swapping^81^ with subsequent detection of a minor keratinocyte cluster in the synovial cell dataset. The swapped molecules and empty droplets were removed at the beginning of the analysis using the R package DropletUtils.^82^^,^^83^ The resulting filtered count matrix was then used for downstream analysis. No special enrichment for neutrophils was done.^68^ The analysis was performed in R (version 4.0.3)^84^ using Bioconductor (version 3.12)^85^ packages. The preprocessing consisted of the following steps: (heterotypic) doublet detection and removal using scDblFinder,^86^ cell filtering using scuttle^87^ and SampleQC^88^ and normalization (log-transformed normalized expression values) using scuttle. Highly variable genes were selected using scran,^89^ and dimensionality reduction was performed using PCA and intrinsicDimension^90^ (selection of PCA components to keep). The data from the two protocols were integrated using a mutual nearest neighbors approach as implemented in the R Bioconductor package batchelor. The batchelor package has been specifically created for the integration of datasets from different batches, such as different experimental factors. Batches were removed by matching mutual nearest neighbors.^91^ Evaluation of batch integration was done using CellMixS.^92^ The cell-type assignment was done in sequential steps. We identified the main synovial cell types first and then assigned cell subtypes within the main cell types individually. The first step of the main cell annotation was graph-based clustering (shared nearest neighbor graph and louvain clustering) with bluster^93^ followed by manual annotation (second step) with assistance from automatic cell type assignment using CellID,^94^ cluster-specific/enriched genes and known marker genes. Cell subtype annotation followed the same steps as the main cell type annotation, starting from highly variable gene selection and using only the respective cell subset. Proportions of neutrophils were calculated by dividing the total number of neutrophils in a sample by the total number of cells in the respective sample. Marker genes were determined with pairwise Wilcoxon tests (using scran). We combined these marker genes with known cell markers and manually selected genes identified in the cluster comparison analysis based on the log-fold change expression (IlogFCI>1) to create the heatmaps. Differential abundance analysis of cell types was conducted using edgeR.^95^ Figures were generated with ggplot2^96^ and ComplexHeatmap.^97^ Trajectory analysis was performed on PECAM1+ endothelial cells. First, proliferating and lymphatic endothelial cells were removed, while all venous endothelial cell subsets were aggregated into a single venous endothelial cell cluster. A trajectory was then inferred on the batch-corrected counts using slingshot.^98^ Testing the association of genes with pseudotime was done using tradeSeq.^99^ The top identified genes were then manually interpreted for biological relevance. To plot gene co-expression in single cells, we calculated the geometric mean of the expression of the two genes of interest; the calculated geometric mean values were visualized in the UMAP embedding, with color indicating the geometric mean of the expression of the two genes of interest.

#### Integration of scRNA-seq dataset with publicly available human synovial scRNA-seq data

Our in-house dataset was integrated with datasets from Wei K et al.^23^ (scRNA-seq profiles of sorted stromal cells from cryopreserved synovial tissues of six RA patients) and Stephenson W et al.^17^ (scRNA-seq data from non-sorted, fresh-dissociated synovial cells from 5 RA patients). The data from Wei K et al.^23^ was preprocessed as described above since fastq files were available. The data from Stephenson W. et al.^17^ was available as a filtered count matrix. The three data sets (in-house, Wei K. et al.^23^, Stephenson W et al.^17^) were integrated by keeping only the union of observed genes. The integrated data was processed in the same way as described above. A batch correction was performed using batchelor, which allowed us to calculate the corrected log-expression values that were then used for label transfer using SingleR.^100^ As a reference, we used our analyzed and annotated reference synovium map with single-cell profiles from 25 fresh synovial tissue samples of patients with inflammatory arthritis (Figure 8).

## References

[1] Feld J., Chandran V., Haroon N., Inman R., Gladman D. (2018). Axial disease in psoriatic arthritis and ankylosing spondylitis: a critical comparison. Nat. Rev. Rheumatol..

[2] Aletaha D., Neogi T., Silman A.J., Funovits J., Felson D.T., Bingham C.O., Birnbaum N.S., Burmester G.R., Bykerk V.P., Cohen M.D. (2010). 2010 Rheumatoid arthritis classification criteria: An American College of Rheumatology/European League Against Rheumatism collaborative initiative. Arthritis Rheum..

[3] Karmacharya P., Chakradhar R., Ogdie A. (2021). The epidemiology of psoriatic arthritis: A literature review. Best Pract. Res. Clin. Rheumatol..

[4] Gibofsky A. (2012). Overview of epidemiology, pathophysiology, and diagnosis of rheumatoid arthritis. Am. J. Manag. Care.

[5] Stolwijk C., van Onna M., Boonen A., van Tubergen A. (2016). Global prevalence of spondyloarthritis: a systematic review and meta-regression analysis. Arthritis Care Res..

[6] Veale D.J., Fearon U. (2018). The pathogenesis of psoriatic arthritis. Lancet.

[7] Cantini F., Niccoli L., Nannini C., Cassara E., Kaloudi O., Favalli E.G., Becciolini A., Biggioggero M., Benucci M., Gobbi F.L. (2016). Seminars in Arthritis and Rheumatism.

[8] Pitzalis C., Choy E.H.S., Buch M.H. (2020). Transforming clinical trials in rheumatology: towards patient-centric precision medicine. Nat. Rev. Rheumatol..

[9] Schett G. (2019). Resolution of inflammation in arthritis. Semin. Immunopathol..

[10] Pratt A.G., Siebert S., Cole M., Stocken D.D., Yap C., Kelly S., Shaikh M., Cranston A., Morton M., Walker J. (2021). Targeting synovial fibroblast proliferation in rheumatoid arthritis (TRAFIC): an open-label, dose-finding, phase 1b trial. Lancet. Rheumatol..

[11] Schett G., Lories R.J., D’Agostino M.-A., Elewaut D., Kirkham B., Soriano E.R., McGonagle D. (2017). Enthesitis: from pathophysiology to treatment. Nat. Rev. Rheumatol..

[12] Orr C., Vieira-Sousa E., Boyle D.L., Buch M.H., Buckley C.D., Cañete J.D., Catrina A.I., Choy E.H.S., Emery P., Fearon U. (2017). Synovial tissue research: a state-of-the-art review. Nat. Rev. Rheumatol..

[13] Boutet M.-A., Nerviani A., Lliso-Ribera G., Lucchesi D., Prediletto E., Ghirardi G.M., Goldmann K., Lewis M., Pitzalis C. (2020). Interleukin-36 family dysregulation drives joint inflammation and therapy response in psoriatic arthritis. Rheumatology.

[14] Humby F., Lewis M., Ramamoorthi N., Hackney J.A., Barnes M.R., Bombardieri M., Setiadi A.F., Kelly S., Bene F., DiCicco M. (2019). Synovial cellular and molecular signatures stratify clinical response to csDMARD therapy and predict radiographic progression in early rheumatoid arthritis patients. Ann. Rheum. Dis..

[15] Kelly S., Humby F., Filer A., Ng N., Di Cicco M., Hands R.E., Rocher V., Bombardieri M., D’Agostino M.A., McInnes I.B. (2015). Ultrasound-guided synovial biopsy: a safe, well-tolerated and reliable technique for obtaining high-quality synovial tissue from both large and small joints in early arthritis patients. Ann. Rheum. Dis..

[16] Zhang F., Wei K., Slowikowski K., Fonseka C.Y., Rao D.A., Kelly S., Goodman S.M., Tabechian D., Hughes L.B., Salomon-Escoto K. (2019). Defining inflammatory cell states in rheumatoid arthritis joint synovial tissues by integrating single-cell transcriptomics and mass cytometry. Nat. Immunol..

[17] Stephenson W., Donlin L.T., Butler A., Rozo C., Bracken B., Rashidfarrokhi A., Goodman S.M., Ivashkiv L.B., Bykerk V.P., Orange D.E. (2018). Single-cell RNA-seq of rheumatoid arthritis synovial tissue using low-cost microfluidic instrumentation. Nat. Commun..

[18] Alivernini S., MacDonald L., Elmesmari A., Finlay S., Tolusso B., Gigante M.R., Petricca L., Di Mario C., Bui L., Perniola S. (2020). Distinct synovial tissue macrophage subsets regulate inflammation and remission in rheumatoid arthritis. Nat. Med..

[19] Culemann S., Grüneboom A., Nicolás-Ávila J.Á., Weidner D., Lämmle K.F., Rothe T., Quintana J.A., Kirchner P., Krljanac B., Eberhardt M. (2019). Locally renewing resident synovial macrophages provide a protective barrier for the joint. Nature.

[20] Croft A.P., Campos J., Jansen K., Turner J.D., Marshall J., Attar M., Savary L., Wehmeyer C., Naylor A.J., Kemble S. (2019). Distinct fibroblast subsets drive inflammation and damage in arthritis. Nature.

[21] Mizoguchi F., Slowikowski K., Wei K., Marshall J.L., Rao D.A., Chang S.K., Nguyen H.N., Noss E.H., Turner J.D., Earp B.E. (2018). Functionally distinct disease-associated fibroblast subsets in rheumatoid arthritis. Nat. Commun..

[22] Donlin L.T., Rao D.A., Wei K., Slowikowski K., McGeachy M.J., Turner J.D., Meednu N., Mizoguchi F., Gutierrez-Arcelus M., Lieb D.J. (2018). Methods for high-dimensional analysis of cells dissociated from cryopreserved synovial tissue. Arthritis Res. Ther..

[23] Wei K., Korsunsky I., Marshall J.L., Gao A., Watts G.F.M., Major T., Croft A.P., Watts J., Blazar P.E., Lange J.K. (2020). Notch signalling drives synovial fibroblast identity and arthritis pathology. Nature.

[24] Burja B., Paul D., Tastanova A., Edalat S.G., Gerber R., Houtman M., Elhai M., Bürki K., Staeger R., Restivo G. (2022). An optimized tissue dissociation protocol for single-cell RNA sequencing analysis of fresh and cultured human skin biopsies. Front. Cell Dev. Biol..

[25] Micheroli R., Elhai M., Edalat S., Frank-Bertoncelj M., Bürki K., Ciurea A., MacDonald L., Kurowska-Stolarska M., Lewis M.J., Goldmann K. (2022). Role of synovial fibroblast subsets across synovial pathotypes in rheumatoid arthritis: a deconvolution analysis. RMD Open.

[26] Krenn V., Morawietz L., Häupl T., Neidel J., Petersen I., König A. (2002). Grading of chronic synovitis—a histopathological grading system for molecular and diagnostic pathology. Pathol. Res. Pract..

[27] (2021). Chromium Single Cell Applications - Guidelines for Optimal Sample Preparation. https://support.10xgenomics.com/single-cell-vdj/index/doc/technical-note-chromium-single-cell-applications-guidelines-for-optimal-sample-preparation.

[28] Zhu X., Guo W. (2021). Meta-Analyses of Multiple Gene Expression Profiles to Screen Hub Genes Related to Osteoarthritis. Public Health Genomics.

[29] Godmann L., Bollmann M., Korb-Pap A., König U., Sherwood J., Beckmann D., Mühlenberg K., Echtermeyer F., Whiteford J., De Rossi G. (2020). Antibody-mediated inhibition of syndecan-4 dimerisation reduces interleukin (IL)-1 receptor trafficking and signalling. Ann. Rheum. Dis..

[30] Rivellese F., Surace A.E.A., Goldmann K., Sciacca E., Çubuk C., Giorli G., John C.R., Nerviani A., Fossati-Jimack L., Thorborn G. (2022). Rituximab versus tocilizumab in rheumatoid arthritis: synovial biopsy-based biomarker analysis of the phase 4 R4RA randomized trial. Nat. Med..

[31] Dick S.A., Wong A., Hamidzada H., Nejat S., Nechanitzky R., Vohra S., Mueller B., Zaman R., Kantores C., Aronoff L. (2022). Three tissue resident macrophage subsets coexist across organs with conserved origins and life cycles. Sci. Immunol..

[32] Zhang F., Mears J.R., Shakib L., Beynor J.I., Shanaj S., Korsunsky I., Nathan A., Donlin L.T., Raychaudhuri S., Accelerating Medicines Partnership Rheumatoid Arthritis and Systemic Lupus Erythematosus (AMP RA/SLE) Consortium (2021). IFN-γ and TNF-α drive a CXCL10+ CCL2+ macrophage phenotype expanded in severe COVID-19 lungs and inflammatory diseases with tissue inflammation. Genome Med..

[33] Remmerie A., Martens L., Thoné T., Castoldi A., Seurinck R., Pavie B., Roels J., Vanneste B., De Prijck S., Vanhockerhout M. (2020). Osteopontin expression identifies a subset of recruited macrophages distinct from Kupffer cells in the fatty liver. Immunity.

[34] Henn D., Chen K., Fehlmann T., Trotsyuk A.A., Sivaraj D., Maan Z.N., Bonham C.A., Barrera J.A., Mays C.J., Greco A.H. (2021). Xenogeneic skin transplantation promotes angiogenesis and tissue regeneration through activated Trem2+ macrophages. Sci. Adv..

[35] Molgora M., Esaulova E., Vermi W., Hou J., Chen Y., Luo J., Brioschi S., Bugatti M., Omodei A.S., Ricci B. (2020). TREM2 modulation remodels the tumor myeloid landscape enhancing anti-PD-1 immunotherapy. Cell.

[36] Trilleaud C., Gauttier V., Biteau K., Girault I., Belarif L., Mary C., Pengam S., Teppaz G., Thepenier V., Danger R. (2021). Agonist anti-ChemR23 mAb reduces tissue neutrophil accumulation and triggers chronic inflammation resolution. Sci. Adv..

[37] Park I., Goddard M.E., Cole J.E., Zanin N., Lyytikäinen L.P., Lehtimäki T., Andreakos E., Feldmann M., Udalova I., Drozdov I., Monaco C. (2022). C-type lectin receptor CLEC4A2 promotes tissue adaptation of macrophages and protects against atherosclerosis. Nat. Commun..

[38] Rantakari P., Patten D.A., Valtonen J., Karikoski M., Gerke H., Dawes H., Laurila J., Ohlmeier S., Elima K., Hübscher S.G. (2016). Stabilin-1 expression defines a subset of macrophages that mediate tissue homeostasis and prevent fibrosis in chronic liver injury. Proc. Natl. Acad. Sci. USA.

[39] Jia D., Chen S., Bai P., Luo C., Liu J., Sun A., Ge J. (2022). Cardiac resident macrophage-derived legumain improves cardiac repair by promoting clearance and degradation of apoptotic cardiomyocytes after myocardial infarction. Circulation.

[40] Dick S.A., Macklin J.A., Nejat S., Momen A., Clemente-Casares X., Althagafi M.G., Chen J., Kantores C., Hosseinzadeh S., Aronoff L. (2019). Self-renewing resident cardiac macrophages limit adverse remodeling following myocardial infarction. Nat. Immunol..

[41] Opzoomer J.W., Anstee J.E., Dean I., Hill E.J., Bouybayoune I., Caron J., Muliaditan T., Gordon P., Sosnowska D., Nuamah R. (2021). Macrophages orchestrate the expansion of a proangiogenic perivascular niche during cancer progression. Sci. Adv..

[42] Ma Y.J., Hein E., Munthe-Fog L., Skjoedt M.-O., Bayarri-Olmos R., Romani L., Garred P. (2015). Soluble collectin-12 (CL-12) is a pattern recognition molecule initiating complement activation via the alternative pathway. J. Immunol..

[43] Ohtani K., Suzuki Y., Eda S., Kawai T., Kase T., Keshi H., Sakai Y., Fukuoh A., Sakamoto T., Itabe H. (2001). The membrane-type collectin CL-P1 is a scavenger receptor on vascular endothelial cells. J. Biol. Chem..

[44] Bogie J.F.J., Mailleux J., Wouters E., Jorissen W., Grajchen E., Vanmol J., Wouters K., Hellings N., van Horssen J., Vanmierlo T., Hendriks J.J.A. (2017). Scavenger receptor collectin placenta 1 is a novel receptor involved in the uptake of myelin by phagocytes. Sci. Rep..

[45] Keerthivasan S., Şenbabaoğlu Y., Martinez-Martin N., Husain B., Verschueren E., Wong A., Yang Y.A., Sun Y., Pham V., Hinkle T. (2021). Homeostatic functions of monocytes and interstitial lung macrophages are regulated via collagen domain-binding receptor LAIR1. Immunity.

[46] Mendez-Enriquez E., García-Zepeda E.A. (2013). The multiple faces of CCL13 in immunity and inflammation. Inflammopharmacology.

[47] Chenivesse C., Tsicopoulos A. (2018). CCL18–Beyond chemotaxis. Cytokine.

[48] Liu J., Song X., Kuang F., Zhang Q., Xie Y., Kang R., Kroemer G., Tang D. (2021). NUPR1 is a critical repressor of ferroptosis. Nat. Commun..

[49] Alshehri F.S.M., Whyte C.S., Mutch N.J. (2021). Factor XIII-A: an indispensable “factor” in haemostasis and wound healing. Int. J. Mol. Sci..

[50] Zaman R., Hamidzada H., Kantores C., Wong A., Dick S.A., Wang Y., Momen A., Aronoff L., Lin J., Razani B. (2021). Selective loss of resident macrophage-derived insulin-like growth factor-1 abolishes adaptive cardiac growth to stress. Immunity.

[51] Trizzino M., Zucco A., Deliard S., Wang F., Barbieri E., Veglia F., Gabrilovich D., Gardini A. (2021). EGR1 is a gatekeeper of inflammatory enhancers in human macrophages. Sci. Adv..

[52] Donovan A., Lima C.A., Pinkus J.L., Pinkus G.S., Zon L.I., Robine S., Andrews N.C. (2005). The iron exporter ferroportin/Slc40a1 is essential for iron homeostasis. Cell Metab..

[53] Theurl I., Hilgendorf I., Nairz M., Tymoszuk P., Haschka D., Asshoff M., He S., Gerhardt L.M.S., Holderried T.A.W., Seifert M. (2016). On-demand erythrocyte disposal and iron recycling requires transient macrophages in the liver. Nat. Med..

[54] Schupp J.C., Adams T.S., Cosme C., Raredon M.S.B., Yuan Y., Omote N., Poli S., Chioccioli M., Rose K.-A., Manning E.P. (2021). Integrated single-cell atlas of endothelial cells of the human lung. Circulation.

[55] Rowe R.G., Weiss S.J. (2008). Breaching the basement membrane: who, when and how?. Trends Cell Biol..

[56] Rantakari P., Jäppinen N., Lokka E., Mokkala E., Gerke H., Peuhu E., Ivaska J., Elima K., Auvinen K., Salmi M. (2016). Fetal liver endothelium regulates the seeding of tissue-resident macrophages. Nature.

[57] Otani T., Furuse M. (2020). Tight junction structure and function revisited. Trends Cell Biol..

[58] Claesson-Welsh L., Dejana E., McDonald D.M. (2021). Permeability of the endothelial barrier: identifying and reconciling controversies. Trends Mol. Med..

[59] Marcos-Ramiro B., García-Weber D., Barroso S., Feito J., Ortega M.C., Cernuda-Morollón E., Reglero-Real N., Fernández-Martín L., Durán M.C., Alonso M.A. (2016). RhoB controls endothelial barrier recovery by inhibiting Rac1 trafficking to the cell border. J. Cell Biol..

[60] Liu X., Uemura A., Fukushima Y., Yoshida Y., Hirashima M. (2016). Semaphorin 3G provides a repulsive guidance cue to lymphatic endothelial cells via Neuropilin-2/PlexinD1. Cell Rep..

[61] Jones R.C., Karkanias J., Krasnow M.A., Pisco A.O., Quake S.R., Salzman J., Yosef N., Bulthaup B., Brown P., Tabula Sapiens Consortium∗ (2022). The Tabula Sapiens: A multiple-organ, single-cell transcriptomic atlas of humans. Science.

[62] Zhang F., Jonsson A.H., Nathan A., Millard N., Curtis M., Xiao Q., Gutierrez-Arcelus M., Apruzzese W., Watts G.F.M., Weisenfeld D. (2023). Deconstruction of rheumatoid arthritis synovium defines inflammatory subtypes. Nature.

[63] O'Neil L.,J., Kaplan M.,J. (2019). Neutrophils in Rheumatoid Arthritis: Breaking Immune Tolerance and Fueling Disease. Trends Mol. Med..

[64] Orange D.E., Blachere N.E., DiCarlo E.F., Mirza S., Pannellini T., Jiang C.S., Frank M.O., Parveen S., Figgie M.P., Gravallese E.M. (2020). Rheumatoid Arthritis Morning Stiffness Is Associated With Synovial Fibrin and Neutrophils. Arthritis Rheumatol..

[65] O'Neil L.J., Barrera-Vargas A., Sandoval-Heglund D., Merayo-Chalico J., Aguirre-Aguilar E., Aponte A.M., Ruiz-Perdomo Y., Gucek M., El-Gabalawy H., Fox D.A. (2020). Neutrophil-mediated carbamylation promotes articular damage in rheumatoid arthritis. Sci. Adv..

[66] Carmona-Rivera C., Carlucci P.M., Goel R.R., James E., Brooks S.R., Rims C., Hoffmann V., Fox D.A., Buckner J.H., Kaplan M.J. (2020). Neutrophil extracellular traps mediate articular cartilage damage and enhance cartilage component immunogenicity in rheumatoid arthritis. JCI Insight.

[67] Zhang Q., Dehaini D., Zhang Y., Zhou J., Chen X., Zhang L., Fang R.H., Gao W., Zhang L. (2018). Neutrophil membrane-coated nanoparticles inhibit synovial inflammation and alleviate joint damage. Nat. Nanotechnol..

[68] Capturing Neutrophils in 10x Single Cell Gene Expression Data. https://support.10xgenomics.com/single-cell-gene-expression/software/pipelines/latest/tutorials/neutrophils.

[69] Salcher S., Sturm G., Horvath L., Untergasser G., Kuempers C., Fotakis G., Panizzolo E., Martowicz A., Trebo M., Pall G., Sykora M., Augustin F., Schmitz K., Finotello F., Rieder D., Perner S., Sopper S., Wolf D., Pircher A., Trajanoski Z., Gamerith G. (2022). High-resolution single-cell atlas reveals diversity and plasticity of tissue-resident neutrophils in non-small cell lung cancer. Cancer Cell.

[70] Korb-Pap A., Stratis A., Mühlenberg K., Niederreiter B., Hayer S., Echtermeyer F., Stange R., Zwerina J., Pap T., Pavenstädt H. (2012). Early structural changes in cartilage and bone are required for the attachment and invasion of inflamed synovial tissue during destructive inflammatory arthritis. Ann. Rheum. Dis..

[71] Friščić J., Böttcher M., Reinwald C., Bruns H., Wirth B., Popp S.-J., Walker K.I., Ackermann J.A., Chen X., Turner J. (2021). The complement system drives local inflammatory tissue priming by metabolic reprogramming of synovial fibroblasts. Immunity.

[72] Hogan N.T., Whalen M.B., Stolze L.K., Hadeli N.K., Lam M.T., Springstead J.R., Glass C.K., Romanoski C.E. (2017). Transcriptional networks specifying homeostatic and inflammatory programs of gene expression in human aortic endothelial cells. Elife.

[73] Morante-Palacios O., Ciudad L., Micheroli R., de la Calle-Fabregat C., Li T., Barbisan G., Houtman M., Edalat S.G., Frank-Bertoncelj M., Ospelt C., Ballestar E. (2022). Coordinated glucocorticoid receptor and MAFB action induces tolerogenesis and epigenome remodeling in dendritic cells. Nucleic Acids Res..

[74] Rudwaleit M., van der Heijde D., Landewé R., Akkoc N., Brandt J., Chou C.T., Dougados M., Huang F., Gu J., Kirazli Y. (2011). The Assessment of SpondyloArthritis International Society classification criteria for peripheral spondyloarthritis and for spondyloarthritis in general. Ann. Rheum. Dis..

[75] Taylor W., Gladman D., Helliwell P., Marchesoni A., Mease P., Mielants H., CASPAR Study Group (2006). Classification criteria for psoriatic arthritis: development of new criteria from a large international study. Arthritis Rheum..

[76] Najm A., Costantino F., Alivernini S., Alunno A., Bianchi E., Bignall J., Boyce B., Cañete J.D., Carubbi F., Durez P. (2022). EULAR points to consider for minimal reporting requirements in synovial tissue research in rheumatology. Ann. Rheum. Dis..

[77] Saraiva F. (2021). Ultrasound-guided synovial biopsy: a review. Front. Med..

[78] EULAR Ultrasound Scanning Guide. esor.eular.org/course/view.php?id=135.

[79] D’Agostino M.-A., Terslev L., Aegerter P., Backhaus M., Balint P., Bruyn G.A., Filippucci E., Grassi W., Iagnocco A., Jousse-Joulin S. (2017). Scoring ultrasound synovitis in rheumatoid arthritis: a EULAR-OMERACT ultrasound taskforce—Part 1: definition and development of a standardised, consensus-based scoring system. RMD Open.

[80] Frankish A., Diekhans M., Ferreira A.-M., Johnson R., Jungreis I., Loveland J., Mudge J.M., Sisu C., Wright J., Armstrong J. (2019). GENCODE reference annotation for the human and mouse genomes. Nucleic Acids Res..

[81] Sinha R., Stanley G., Gulati G.S., Ezran C., Travaglini K.J., Wei E., Chan C.K., Nabhan A.N., Su T., Morganti R.M. (2017). Index switching causes “spreading-of-signal” among multiplexed samples in Illumina HiSeq 4000 DNA sequencing. BioRxiv.

[82] Griffiths J.A., Richard A.C., Bach K., Lun A.T.L., Marioni J.C. (2018). Detection and removal of barcode swapping in single-cell RNA-seq data. Nat. Commun..

[83] Lun A.T.L., Riesenfeld S., Andrews T., Dao T.P., Gomes T., Marioni J.C., Participants in the 1st Human Cell Atlas Jamboree (2019). EmptyDrops: distinguishing cells from empty droplets in droplet-based single-cell RNA sequencing data. Genome Biol..

[84] Core Team, R. R. R: A Language and Environment for Statistical Computing. (R Foundation for Statistical Computing). https://www.gbif.org/tool/81287/r-a-language-and-environment-for-statistical-computing.

[85] Huber W., Carey V.J., Gentleman R., Anders S., Carlson M., Carvalho B.S., Bravo H.C., Davis S., Gatto L., Girke T. (2015). Orchestrating high-throughput genomic analysis with Bioconductor. Nat. Methods.

[86] Germain P.-L., Lun A., Garcia Meixide C., Macnair W., Robinson M.D. (2021). Doublet identification in single-cell sequencing data using scDblFinder. F1000Res..

[87] McCarthy D.J., Campbell K.R., Lun A.T.L., Wills Q.F. (2017). Scater: pre-processing, quality control, normalization and visualization of single-cell RNA-seq data in R. Bioinformatics.

[88] Macnair W., Robinson M. (2023). SampleQC: robust multivariate, multi-cell type, multi-sample quality control for single-cell data. Genome Biol..

[89] Lun A.T.L., McCarthy D.J., Marioni J.C. (2016). A step-by-step workflow for low-level analysis of single-cell RNA-seq data with Bioconductor. F1000Res..

[90] Johnsson K. (2016).

[91] Haghverdi L., Lun A.T.L., Morgan M.D., Marioni J.C. (2018). Batch effects in single-cell RNA-sequencing data are corrected by matching mutual nearest neighbors. Nat. Biotechnol..

[92] Lütge A., Zyprych-Walczak J., Kunzmann U.B., Crowell H.L., Calini D., Malhotra D., Soneson C., Robinson M.D. (2021). CellMixS: quantifying and visualizing batch effects in single-cell RNA-seq data. Life Sci. Alliance.

[93] Lun A. (2023).

[94] Cortal A., Rausell A. (2020).

[95] Robinson M.D., McCarthy D.J., Smyth G.K. (2010). edgeR: a Bioconductor package for differential expression analysis of digital gene expression data. Bioinformatics.

[96] Wickham H. (2009).

[97] Gu Z., Eils R., Schlesner M. (2016). Complex heatmaps reveal patterns and correlations in multidimensional genomic data. Bioinformatics.

[98] Street K., Risso D., Fletcher R.B., Das D., Ngai J., Yosef N., Purdom E., Dudoit S. (2018). Slingshot: cell lineage and pseudotime inference for single-cell transcriptomics. BMC Genom..

[99] Van den Berge K., Roux de Bézieux H., Street K., Saelens W., Cannoodt R., Saeys Y., Dudoit S., Clement L. (2020). Trajectory-based differential expression analysis for single-cell sequencing data. Nat. Commun..

[100] Aran D., Looney A.P., Liu L., Wu E., Fong V., Hsu A., Chak S., Naikawadi R.P., Wolters P.J., Abate A.R. (2019). Reference-based analysis of lung single-cell sequencing reveals a transitional profibrotic macrophage. Nat. Immunol..

