# Supplementary material for: Molecular maps of synovial cells in inflammatory arthritis using an optimized synovial tissue dissociation protocol
Source: iScience. 2024 Apr 10;27(6):109707. doi: 10.1016/j.isci.2024.109707 (PMC11144743; doi:10.1016/j.isci.2024.109707)
Supplement: Document S1. Figures S1–S18 and Tables S1 and S2 [file mmc1.pdf]

iScience, Volume *27*

Supplemental information

Molecular maps of synovial cells in inflammatory arthritis using an optimized synovial tissue dissociation protocol

Sam G. Edalat, Reto Gerber, Miranda Houtman, Janine Lückgen, Rui Lourenço Teixeira, Maria del Pilar Palacios Cisneros, Tamara Pfanner, Tadeja Kuret, Nadja Ižanc, Raphael Micheroli, Joaquim Polido-Pereira, Fernando Saraiva, Swathi Lingam, Kristina Burki, Blaž Burja, Chantal Pauli, Žiga Rotar, Matija Tomšič, Saša Čučnik, João Eurico Fonseca, Oliver Distler, Ângelo Calado, Vasco C. Romão, Caroline Ospelt, Snežna Sodin-Semrl, Mark D. Robinson, and Mojca Frank Bertoncelj

Figure S1

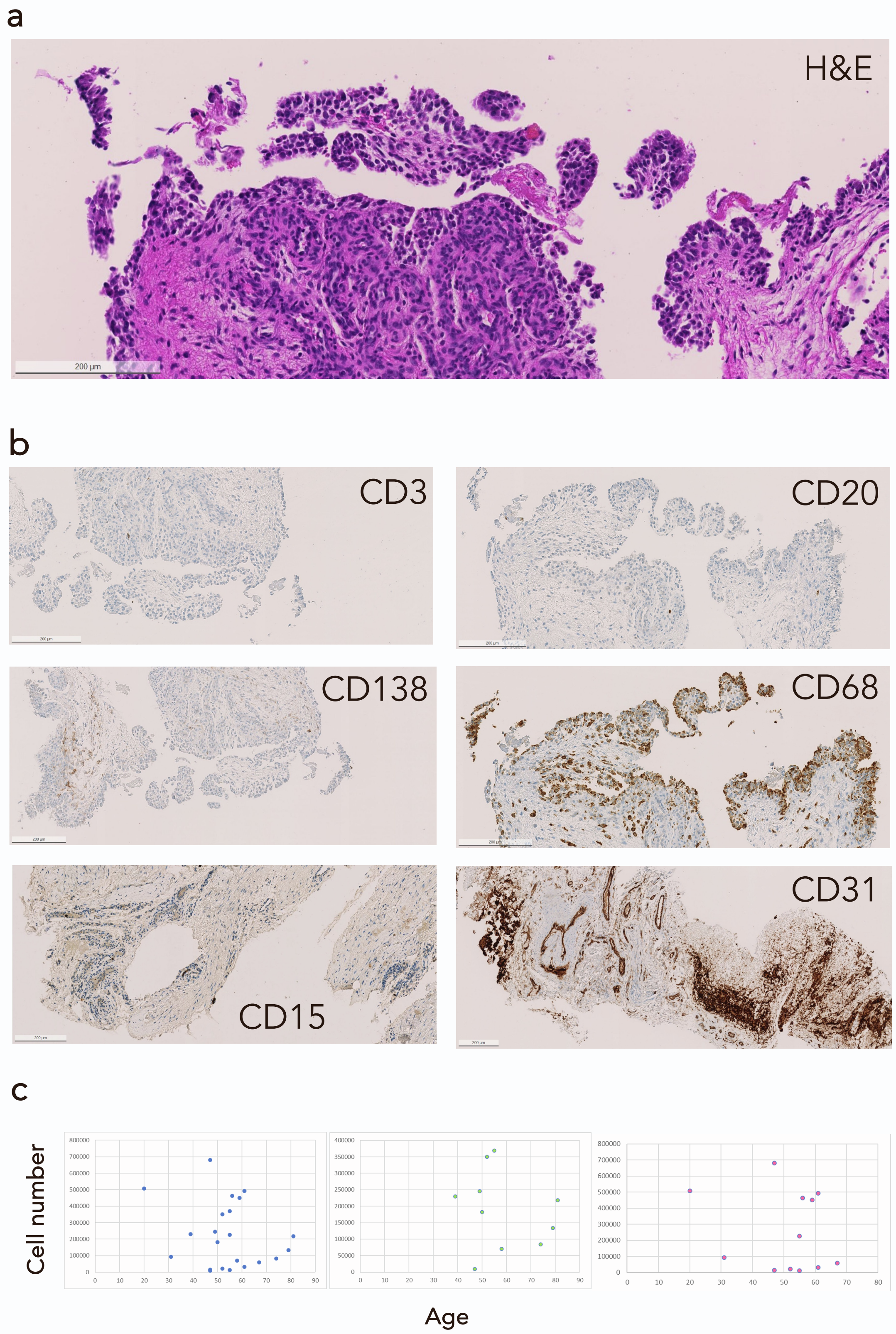

Figure S2

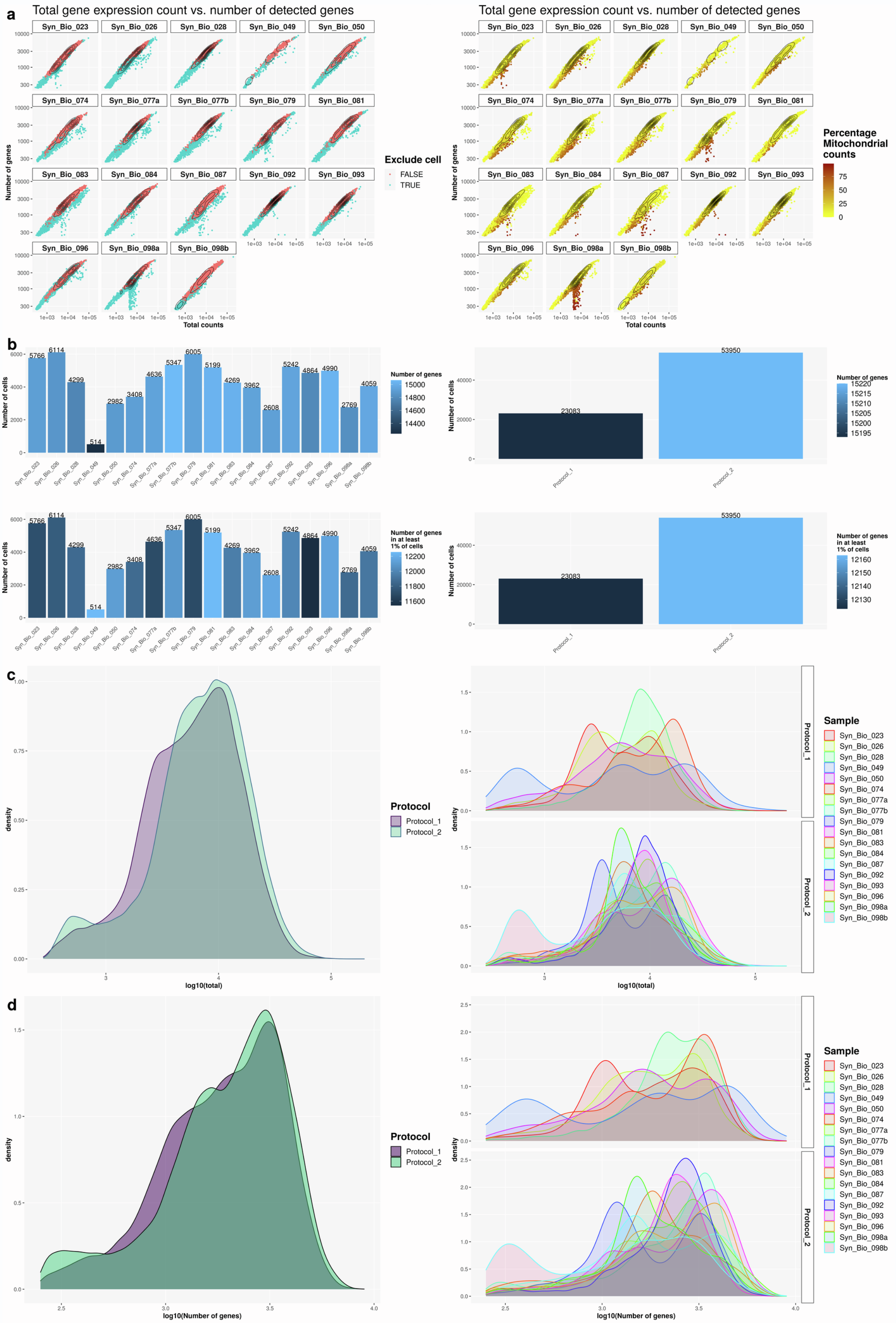
a
Total gene expression count vs. number of detected genes
Syn_Bio_023
Syn_Bio_026
Syn_Bio_028
Syn_Bio_049
Syn_Bio_050
Syn_Bio_074
Syn_Bio_077a
Syn_Bio_077b
Syn_Bio_079
Syn_Bio_081
Syn_Bio_083
Syn_Bio_084
Syn_Bio_087
Syn_Bio_092
Syn_Bio_093
Syn_Bio_096
Syn_Bio_098a
Syn_Bio_098b
Number of genes
Total counts
Exclude cell
FALSE
TRUE
Percentage Mitochondrial counts
75
50
25
0
b
Number of cells
5766
6114
4299
514
2982
3408
4636
5347
6005
5199
4269
3962
2608
5242
4864
4990
2769
4059
Number of genes
15000
14800
14600
14400
23083
53950
Protocol_1
Protocol_2
15220
15215
15210
15205
15200
15195
Number of genes in at least 1% of cells
12200
12000
11800
11600
12160
12150
12140
12130
c
density
log10(total)
Protocol
Protocol_1
Protocol_2
Sample
d
log10(Number of genes)

Figure S3

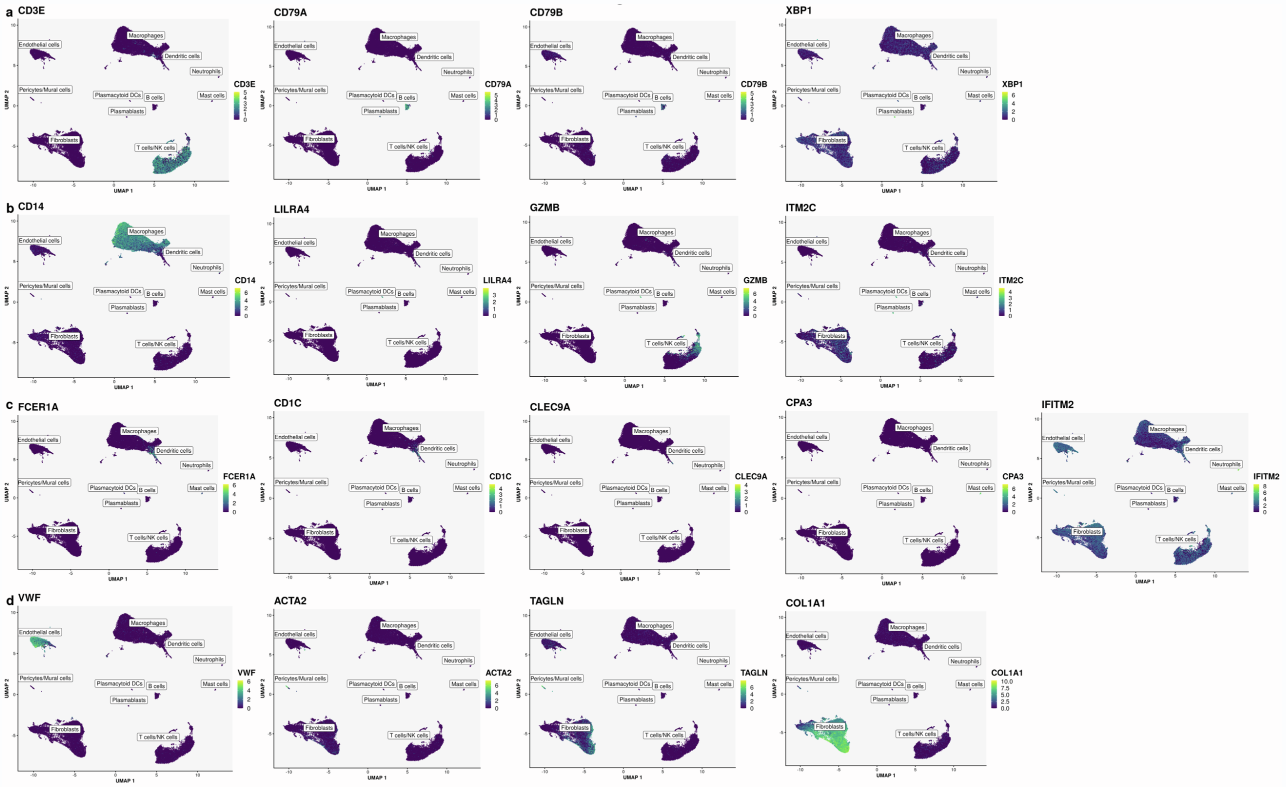

a CD3E
CD79A
CD79B
XBP1
b CD14
LILRA4
GZMB
ITM2C
c FCER1A
CD1C
CLEC9A
CPA3
IFITM2
d VWF
ACTA2
TAGLN
COL1A1
Macrophages
Endothelial cells
Dendritic cells
Neutrophils
Pericytes/Mural cells
Plasmacytoid DCs
B cells
Mast cells
Plasmablasts
Fibroblasts
T cells/NK cells
UMAP 1
UMAP 2

Figure S4

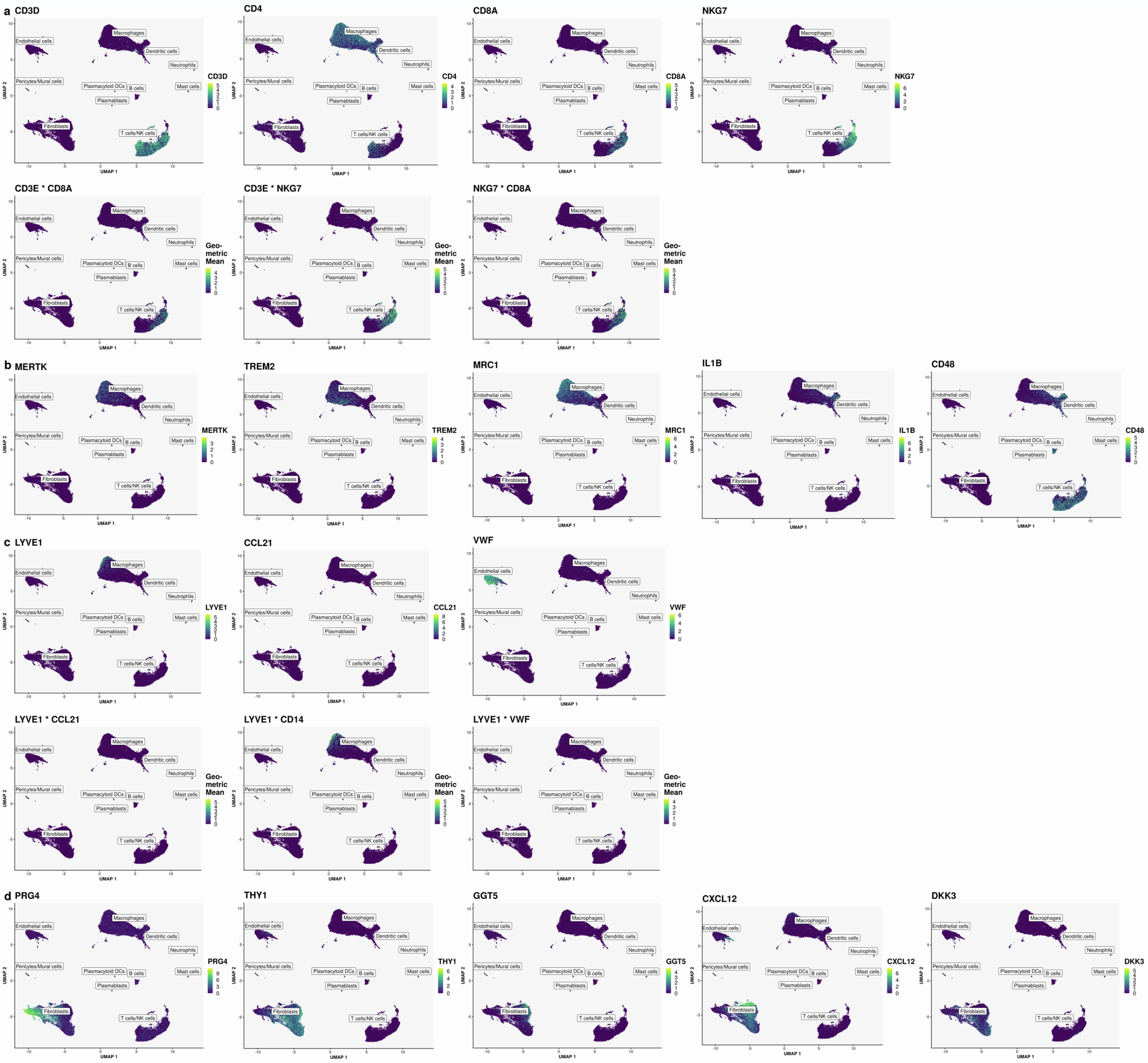

a CD3D
CD4
CD8A
NKG7
CD3E * CD8A
CD3E * NKG7
NKG7 * CD8A
b MERTK
TREM2
MRC1
IL1B
CD48
c LYVE1
CCL21
VWF
LYVE1 * CCL21
LYVE1 * CD14
LYVE1 * VWF
d PRG4
THY1
GGT5
CXCL12
DKK3
Geo-
metric
Mean
Endothelial cells
Macrophages
Dendritic cells
Neutrophils
Pericytes/Mural cells
Plasmacytoid DCs
B cells
Mast cells
Plasmablasts
Fibroblasts
T cells/NK cells
UMAP 1
UMAP 2

Figure S5

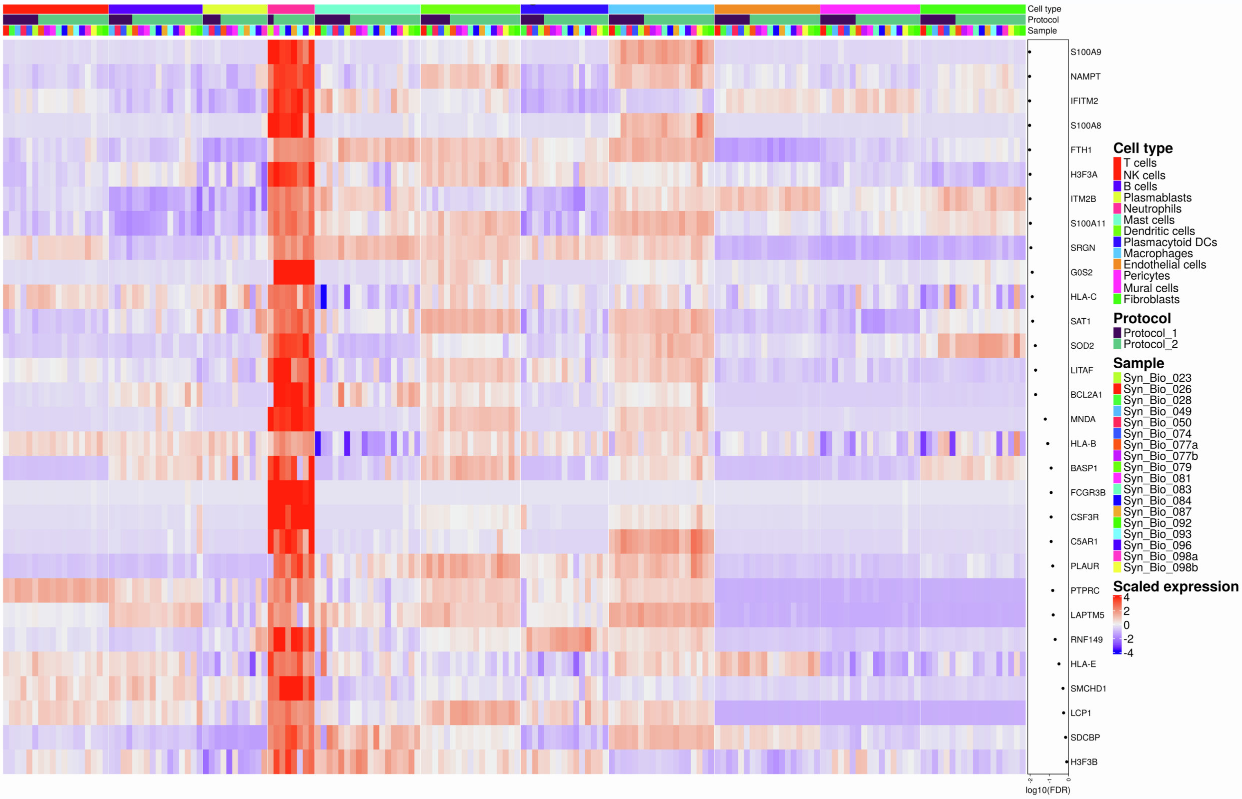
Cell type
Protocol
Sample
S100A9
NAMPT
IFITM2
S100A8
FTH1
H3F3A
ITM2B
S100A11
SRGN
G0S2
HLA-C
SAT1
SOD2
LITAF
BCL2A1
MNDA
HLA-B
BASP1
FCGR3B
CSF3R
C5AR1
PLAUR
PTPRC
LAPTM5
RNF149
HLA-E
SMCHD1
LCP1
SDCBP
H3F3B
log10(FDR)
Cell type
T cells
NK cells
B cells
Plasmablasts
Neutrophils
Mast cells
Dendritic cells
Plasmacytoid DCs
Macrophages
Endothelial cells
Pericytes
Mural cells
Fibroblasts
Protocol
Protocol_1
Protocol_2
Sample
Syn_Bio_023
Syn_Bio_026
Syn_Bio_028
Syn_Bio_049
Syn_Bio_050
Syn_Bio_074
Syn_Bio_077a
Syn_Bio_077b
Syn_Bio_079
Syn_Bio_081
Syn_Bio_083
Syn_Bio_084
Syn_Bio_087
Syn_Bio_092
Syn_Bio_093
Syn_Bio_096
Syn_Bio_098a
Syn_Bio_098b
Scaled expression
4
2
0
-2
-4

Figure S6

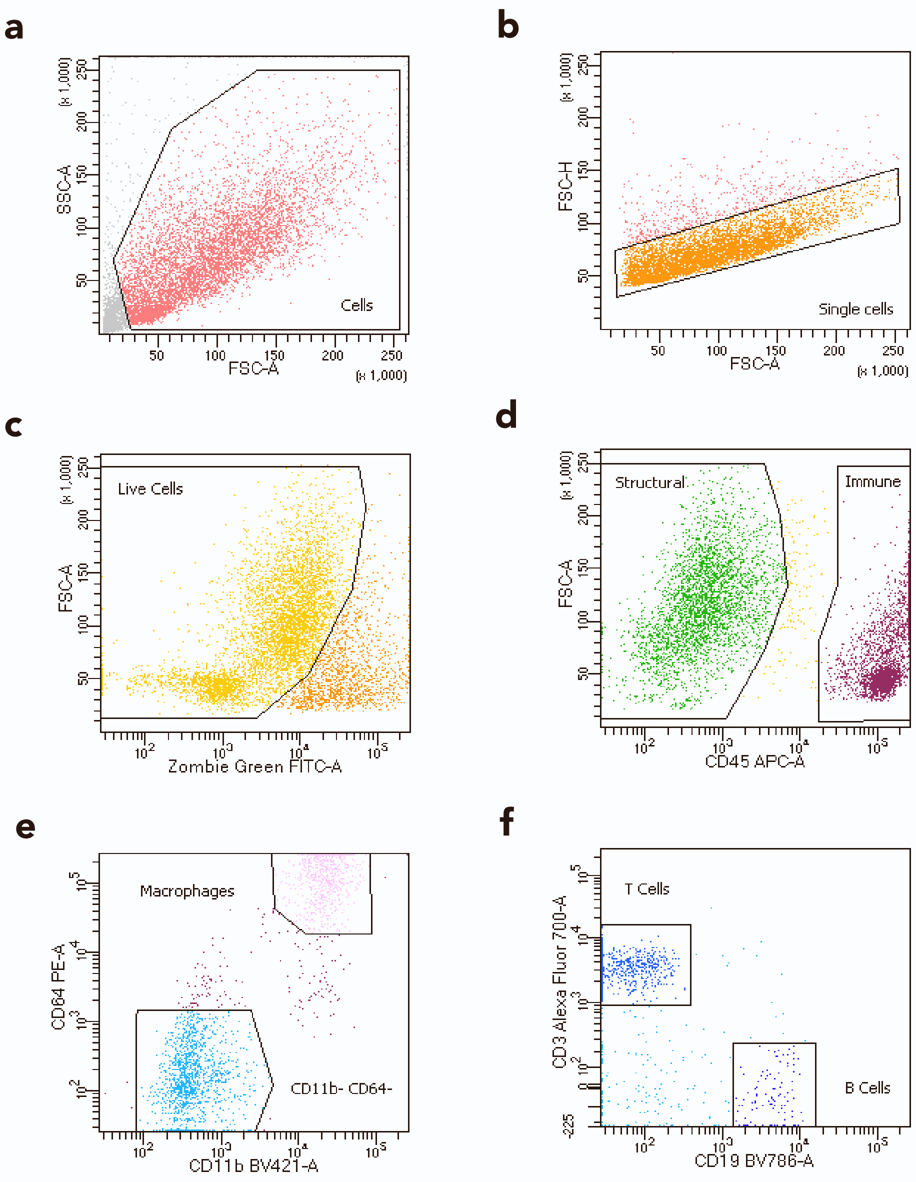

g

Tube: Multicolor Staining + Viability Dye

Population	#Events	%Parent	%Total
All Events	10,121	####	100.0
Cells	7,514	74.2	74.2
Single cells	7,004	93.2	69.2
Live Cells	5,619	80.2	55.5
Structural	3,218	57.3	31.8
Immune	2,270	40.4	22.4
Macrophages	736	32.4	7.3
CD11b- CD64-	1,367	60.2	13.5
T Cells	1,081	79.1	10.7
B Cells	100	7.3	1.0

Figure S7

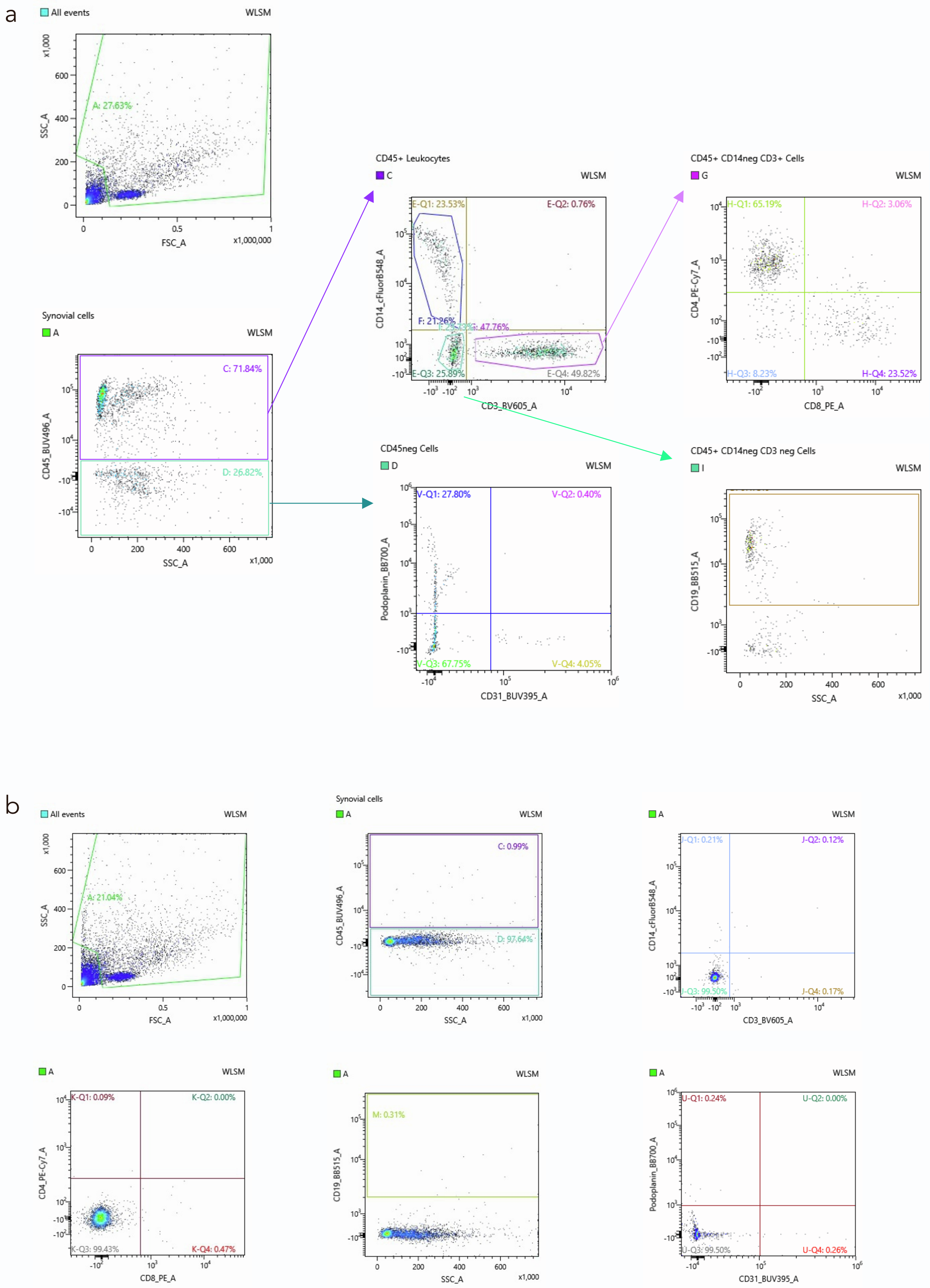
a
All events
WLSM
A: 27.63%
SSC_A
FSC_A
Synovial cells
C: 71.84%
D: 26.82%
CD45_BUV496_A
CD45+ Leukocytes
E-Q1: 23.53%
E-Q2: 0.76%
E-Q3: 25.89%
E-Q4: 49.82%
F: 21.26%
CD14_cFluorB548_A
CD3_BV605_A
CD45+ CD14neg CD3+ Cells
H-Q1: 65.19%
H-Q2: 3.06%
H-Q3: 8.23%
H-Q4: 23.52%
CD4_PE-Cy7_A
CD8_PE_A
CD45neg Cells
V-Q1: 27.80%
V-Q2: 0.40%
V-Q3: 67.75%
V-Q4: 4.05%
Podoplanin_BB700_A
CD31_BUV395_A
CD45+ CD14neg CD3 neg Cells
CD19_BB515_A
b
A: 21.04%
C: 0.99%
D: 97.64%
J-Q1: 0.21%
J-Q2: 0.12%
J-Q3: 99.50%
J-Q4: 0.17%
K-Q1: 0.09%
K-Q2: 0.00%
K-Q3: 99.43%
K-Q4: 0.47%
M: 0.31%
U-Q1: 0.24%
U-Q2: 0.00%
U-Q3: 99.50%
U-Q4: 0.26%

Figure S8

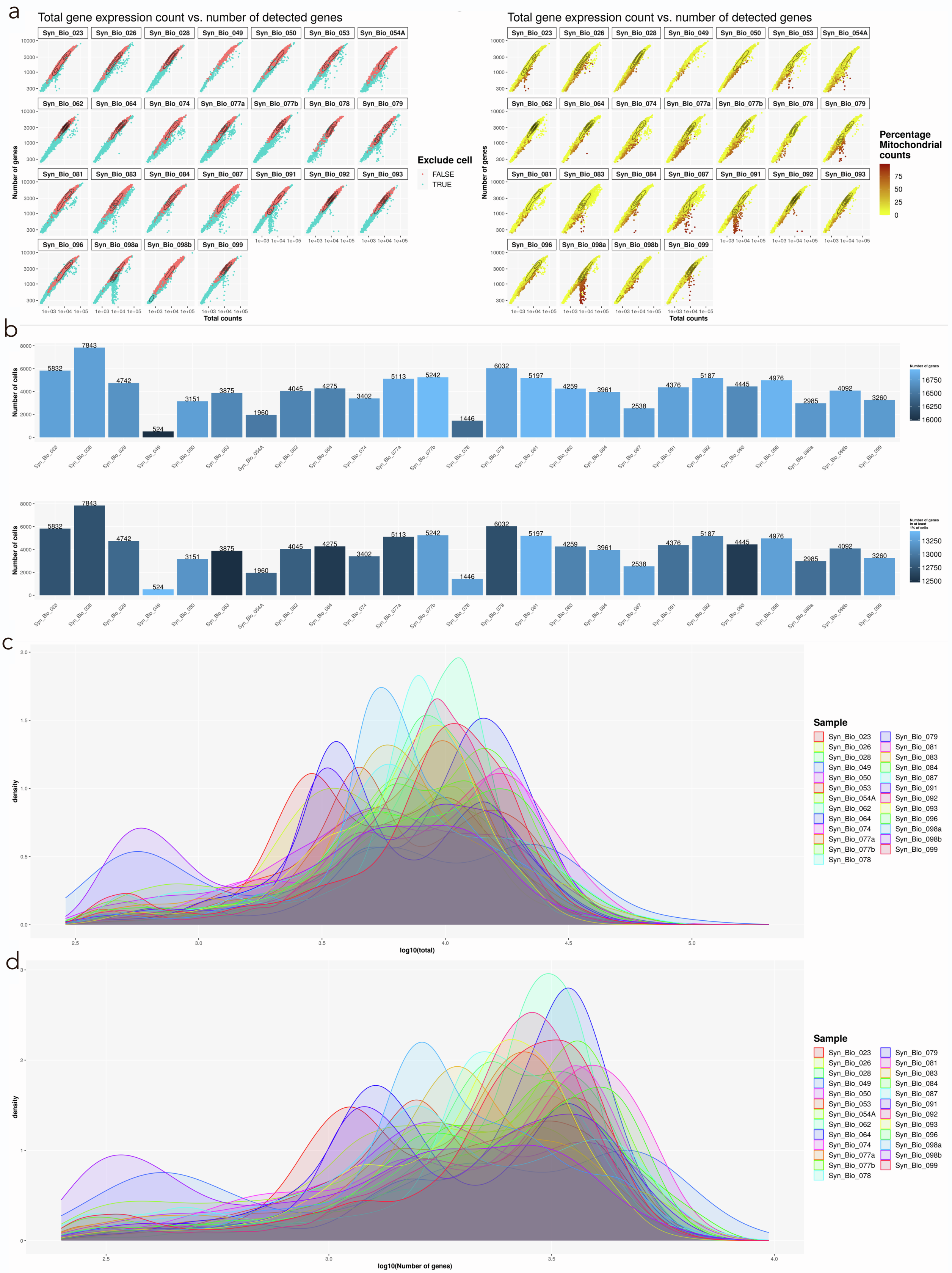
a
Total gene expression count vs. number of detected genes
Total gene expression count vs. number of detected genes
Syn_Bio_023
Syn_Bio_026
Syn_Bio_028
Syn_Bio_049
Syn_Bio_050
Syn_Bio_053
Syn_Bio_054A
Syn_Bio_062
Syn_Bio_064
Syn_Bio_074
Syn_Bio_077a
Syn_Bio_077b
Syn_Bio_078
Syn_Bio_079
Syn_Bio_081
Syn_Bio_083
Syn_Bio_084
Syn_Bio_087
Syn_Bio_091
Syn_Bio_092
Syn_Bio_093
Syn_Bio_096
Syn_Bio_098a
Syn_Bio_098b
Syn_Bio_099
Number of genes
Total counts
Exclude cell
FALSE
TRUE
Percentage Mitochondrial counts
75
50
25
0
b
Number of cells
Number of genes
Number of genes in at least 1% of cells
5832
7843
4742
524
3151
3875
1960
4045
4275
3402
5113
5242
1446
6032
5197
4259
3961
2538
4376
5187
4445
4976
2985
4092
3260
c
density
log10(total)
Sample
d
density
log10(Number of genes)
Sample

Figure S9

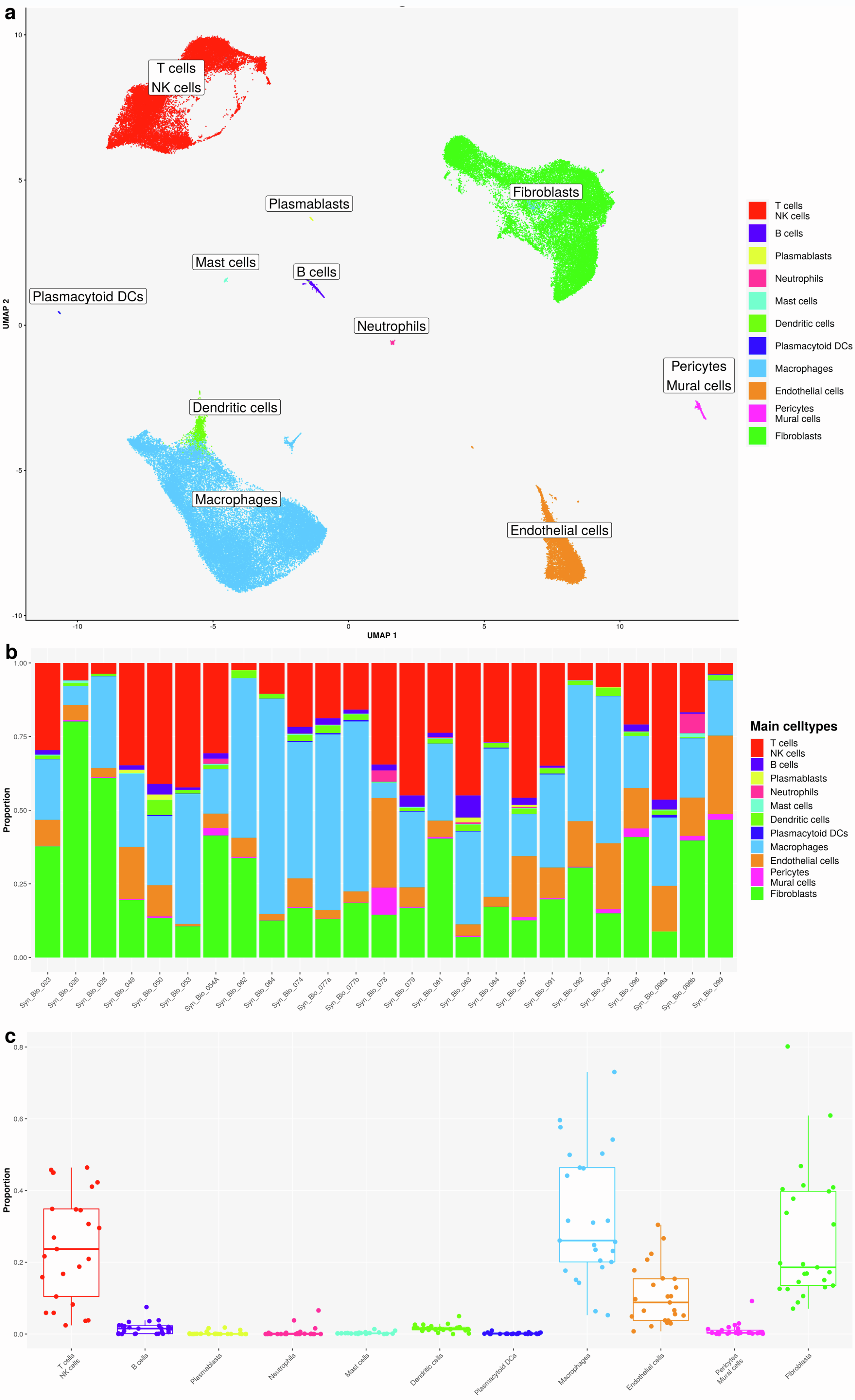
a
T cells
NK cells
Fibroblasts
Plasmablasts
Mast cells
B cells
Plasmacytoid DCs
Neutrophils
Pericytes
Mural cells
Dendritic cells
Macrophages
Endothelial cells
UMAP 1
UMAP 2
b
Proportion
Main celltypes
c
Proportion

Figure S10

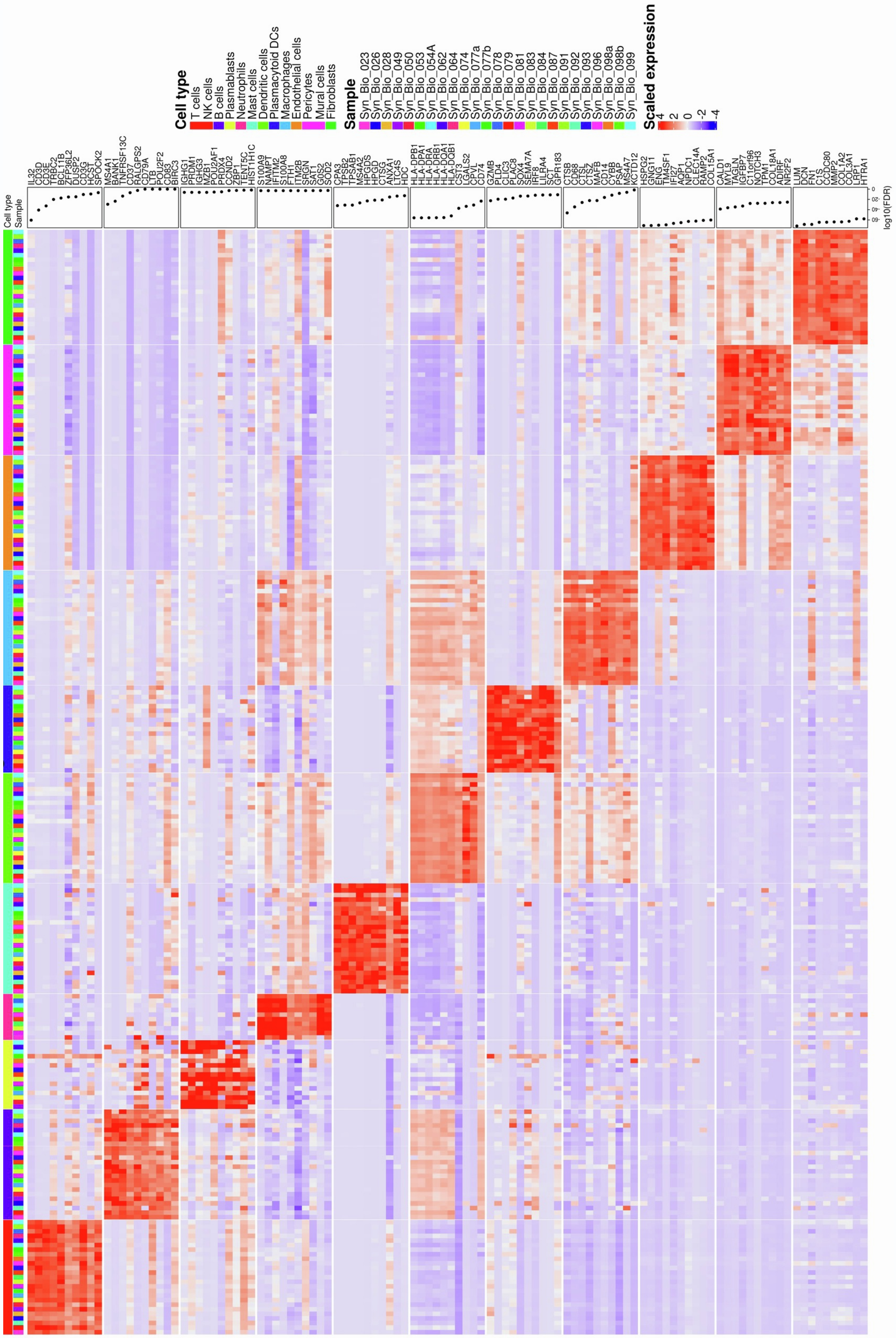

Cell type
T cells
NK cells
B cells
Plasmablasts
Neutrophils
Mast cells
Dendritic cells
Plasmacytoid DCs
Macrophages
Endothelial cells
Pericytes
Mural cells
Fibroblasts
Sample
Scaled expression
log10(FDR)

Figure S11

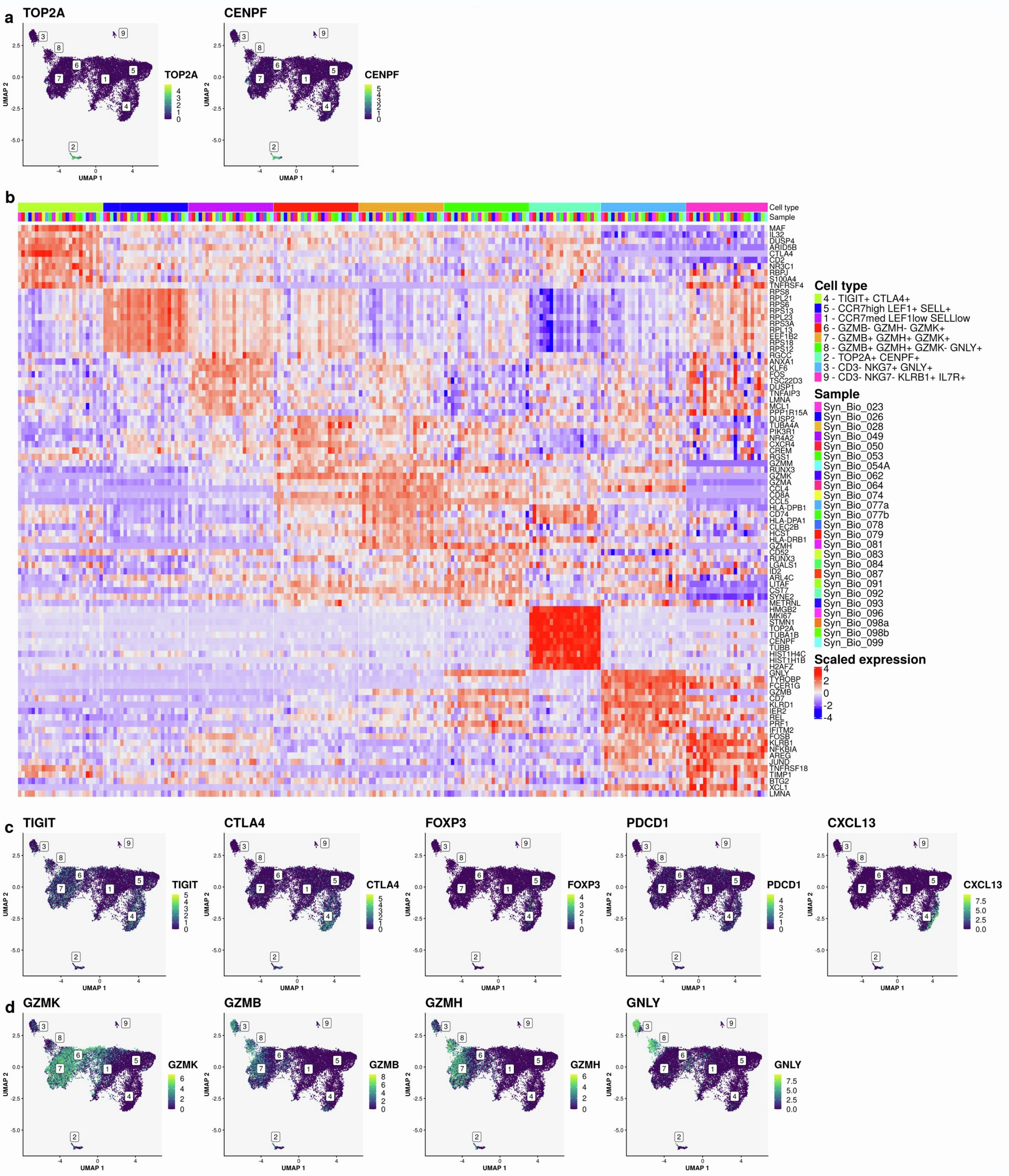

a
TOP2A
CENPF
b
Cell type
Sample
Cell type
4 - TIGIT+ CTLA4+
5 - CCR7high LEF1+ SELL+
1 - CCR7med LEF1low SELLlow
6 - GZMB- GZMH- GZMK+
7 - GZMB+ GZMH+ GZMK+
8 - GZMB+ GZMH+ GZMK- GNLY+
2 - TOP2A+ CENPF+
3 - CD3- NKG7+ GNLY+
9 - CD3- NKG7- KLRB1+ IL7R+
Sample
Syn_Bio_023
Syn_Bio_026
Syn_Bio_028
Syn_Bio_049
Syn_Bio_050
Syn_Bio_053
Syn_Bio_054A
Syn_Bio_062
Syn_Bio_064
Syn_Bio_074
Syn_Bio_077a
Syn_Bio_077b
Syn_Bio_078
Syn_Bio_079
Syn_Bio_081
Syn_Bio_083
Syn_Bio_084
Syn_Bio_087
Syn_Bio_091
Syn_Bio_092
Syn_Bio_093
Syn_Bio_096
Syn_Bio_098a
Syn_Bio_098b
Syn_Bio_099
Scaled expression
c
TIGIT
CTLA4
FOXP3
PDCD1
CXCL13
d
GZMK
GZMB
GZMH
GNLY
UMAP 1
UMAP 2

Figure S12

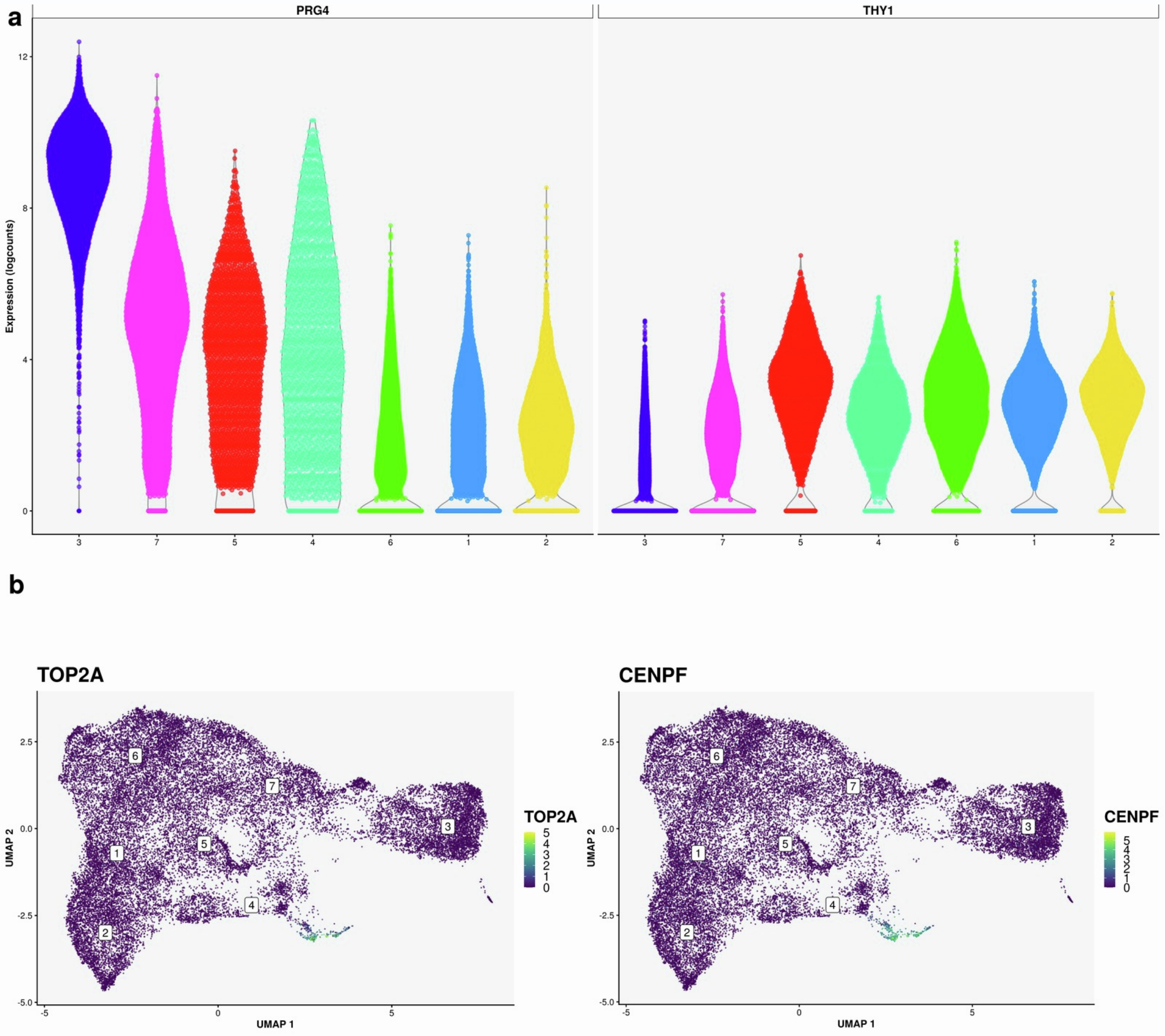
a
PRG4
THY1
Expression (logcounts)
12
8
4
0
3
7
5
4
6
1
2
b
TOP2A
CENPF
UMAP 2
UMAP 1
2.5
0.0
-2.5
-5.0
-5
0
5
5
4
3
2
1
0

Figure S13

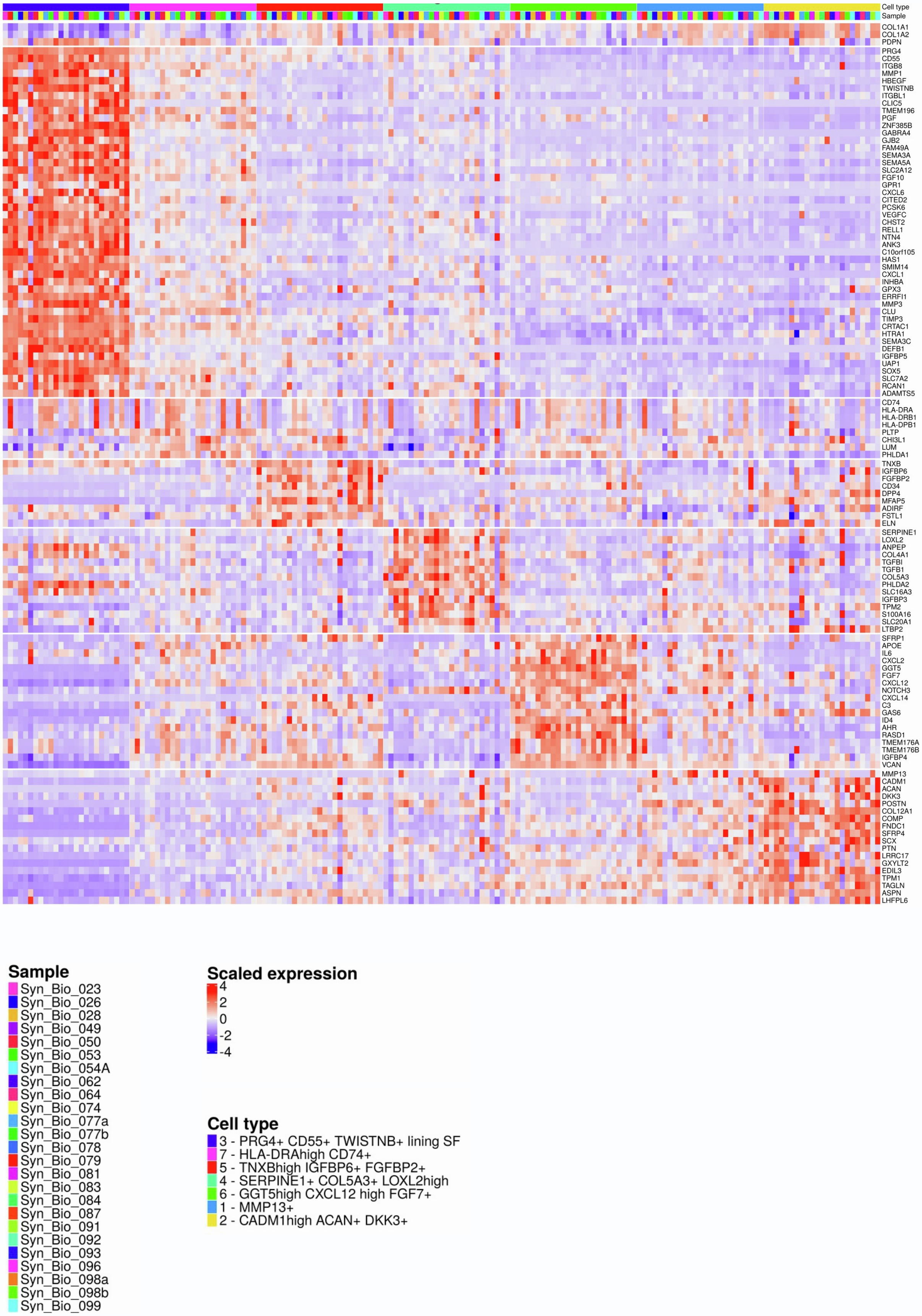
Cell type
Sample
COL1A1
COL1A2
PDPN
PRG4
CD55
ITGB8
MMP1
HBEGF
TWISTNB
ITGBL1
CLIC5
TMEM196
PGF
ZNF385B
GABRA4
GJB2
FAM49A
SEMA3A
SEMA5A
SLC2A12
FGF10
GPR1
CXCL6
CITED2
PCSK6
VEGFC
CHST2
RELL1
NTN4
ANK3
C10orf105
HAS1
SMIM14
CXCL1
INHBA
GPX3
ERRFI1
MMP3
CLU
TIMP3
CRTAC1
HTRA1
SEMA3C
DEFB1
IGFBP5
UAP1
SOX5
SLC7A2
RCAN1
ADAMTS5
CD74
HLA-DRA
HLA-DRB1
HLA-DPB1
PLTP
CHI3L1
LUM
PHLDA1
TNXB
IGFBP6
FGFBP2
CD34
DPP4
MFAP5
ADIRF
FSTL1
ELN
SERPINE1
LOXL2
ANPEP
COL4A1
TGFBI
TGFB1
COL5A3
PHLDA2
SLC16A3
IGFBP3
TPM2
S100A16
SLC20A1
LTBP2
SFRP1
APOE
IL6
CXCL2
GGT5
FGF7
CXCL12
NOTCH3
CXCL14
C3
GAS6
ID4
AHR
RASD1
TMEM176A
TMEM176B
IGFBP4
VCAN
MMP13
CADM1
ACAN
DKK3
POSTN
COL12A1
COMP
FNDC1
SFRP4
SCX
PTN
LRRC17
GXYLT2
EDIL3
TPM1
TAGLN
ASPN
LHFPL6
Sample
Syn_Bio_023
Syn_Bio_026
Syn_Bio_028
Syn_Bio_049
Syn_Bio_050
Syn_Bio_053
Syn_Bio_054A
Syn_Bio_062
Syn_Bio_064
Syn_Bio_074
Syn_Bio_077a
Syn_Bio_077b
Syn_Bio_078
Syn_Bio_079
Syn_Bio_081
Syn_Bio_083
Syn_Bio_084
Syn_Bio_087
Syn_Bio_091
Syn_Bio_092
Syn_Bio_093
Syn_Bio_096
Syn_Bio_098a
Syn_Bio_098b
Syn_Bio_099
Scaled expression
4
2
0
-2
-4
Cell type
3 - PRG4+ CD55+ TWISTNB+ lining SF
7 - HLA-DRAhigh CD74+
5 - TNXBhigh IGFBP6+ FGFBP2+
4 - SERPINE1+ COL5A3+ LOXL2high
6 - GGT5high CXCL12 high FGF7+
1 - MMP13+
2 - CADM1high ACAN+ DKK3+

Figure S14

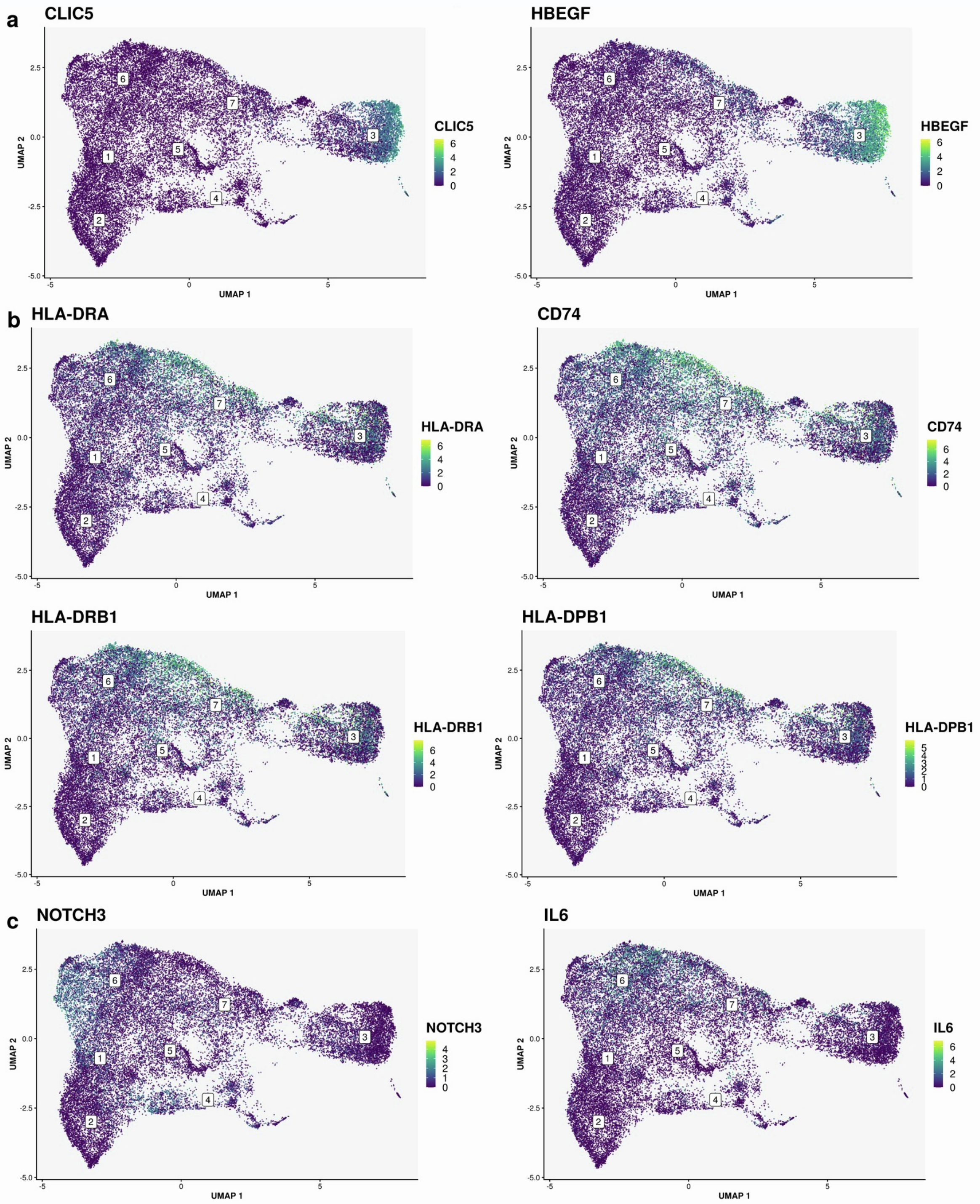

a
CLIC5
HBEGF
b
HLA-DRA
CD74
HLA-DRB1
HLA-DPB1
c
NOTCH3
IL6
UMAP 1
UMAP 2

Figure S15

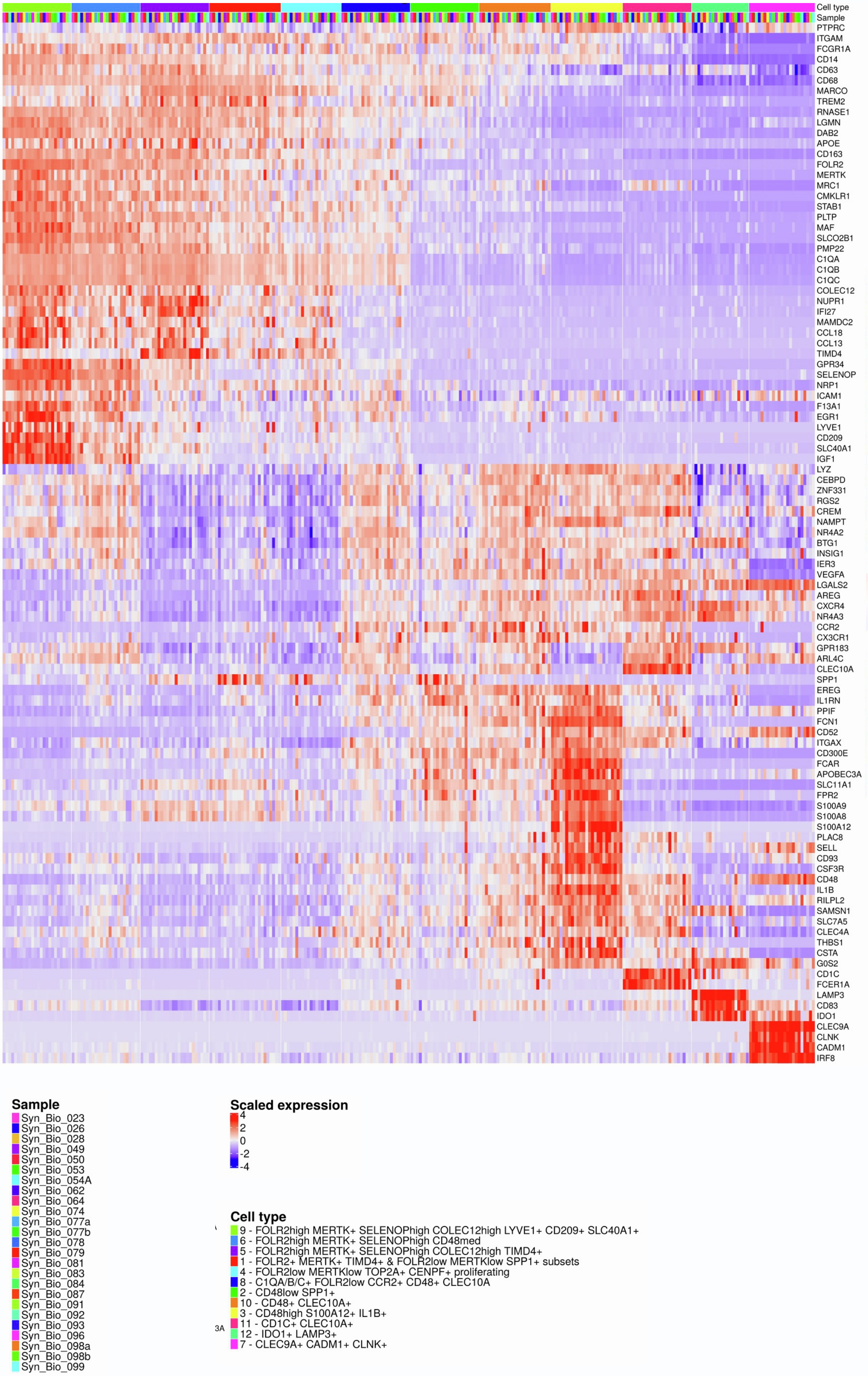

Cell type
Sample
PTPRC
ITGAM
FCGR1A
CD14
CD63
CD68
MARCO
TREM2
RNASE1
LGMN
DAB2
APOE
CD163
FOLR2
MERTK
MRC1
CMKLR1
STAB1
PLTP
MAF
SLCO2B1
PMP22
C1QA
C1QB
C1QC
COLEC12
NUPR1
IFI27
MAMDC2
CCL18
CCL13
TIMD4
GPR34
SELENOP
NRP1
ICAM1
F13A1
EGR1
LYVE1
CD209
SLC40A1
IGF1
LYZ
CEBPD
ZNF331
RGS2
CREM
NAMPT
NR4A2
BTG1
INSIG1
IER3
VEGFA
LGALS2
AREG
CXCR4
NR4A3
CCR2
CX3CR1
GPR183
ARL4C
CLEC10A
SPP1
EREG
IL1RN
PPIF
FCN1
CD52
ITGAX
CD300E
FCAR
APOBEC3A
SLC11A1
FPR2
S100A9
S100A8
S100A12
PLAC8
SELL
CD93
CSF3R
CD48
IL1B
RILPL2
SAMSN1
SLC7A5
CLEC4A
THBS1
CSTA
G0S2
CD1C
FCER1A
LAMP3
CD83
IDO1
CLEC9A
CLNK
CADM1
IRF8
Sample
Syn_Bio_023
Syn_Bio_026
Syn_Bio_028
Syn_Bio_049
Syn_Bio_050
Syn_Bio_053
Syn_Bio_054A
Syn_Bio_062
Syn_Bio_064
Syn_Bio_074
Syn_Bio_077a
Syn_Bio_077b
Syn_Bio_078
Syn_Bio_079
Syn_Bio_081
Syn_Bio_083
Syn_Bio_084
Syn_Bio_087
Syn_Bio_091
Syn_Bio_092
Syn_Bio_093
Syn_Bio_096
Syn_Bio_098a
Syn_Bio_098b
Syn_Bio_099
Scaled expression
4
2
0
-2
-4
Cell type
9 - FOLR2high MERTK+ SELENOPhigh COLEC12high LYVE1+ CD209+ SLC40A1+
6 - FOLR2high MERTK+ SELENOPhigh CD48med
5 - FOLR2high MERTK+ SELENOPhigh COLEC12high TIMD4+
1 - FOLR2+ MERTK+ TIMD4+ & FOLR2low MERTKlow SPP1+ subsets
4 - FOLR2low MERTKlow TOP2A+ CENPF+ proliferating
8 - C1QA/B/C+ FOLR2low CCR2+ CD48+ CLEC10A
2 - CD48low SPP1+
10 - CD48+ CLEC10A+
3 - CD48high S100A12+ IL1B+
11 - CD1C+ CLEC10A+
12 - IDO1+ LAMP3+
7 - CLEC9A+ CADM1+ CLNK+

Figure S16

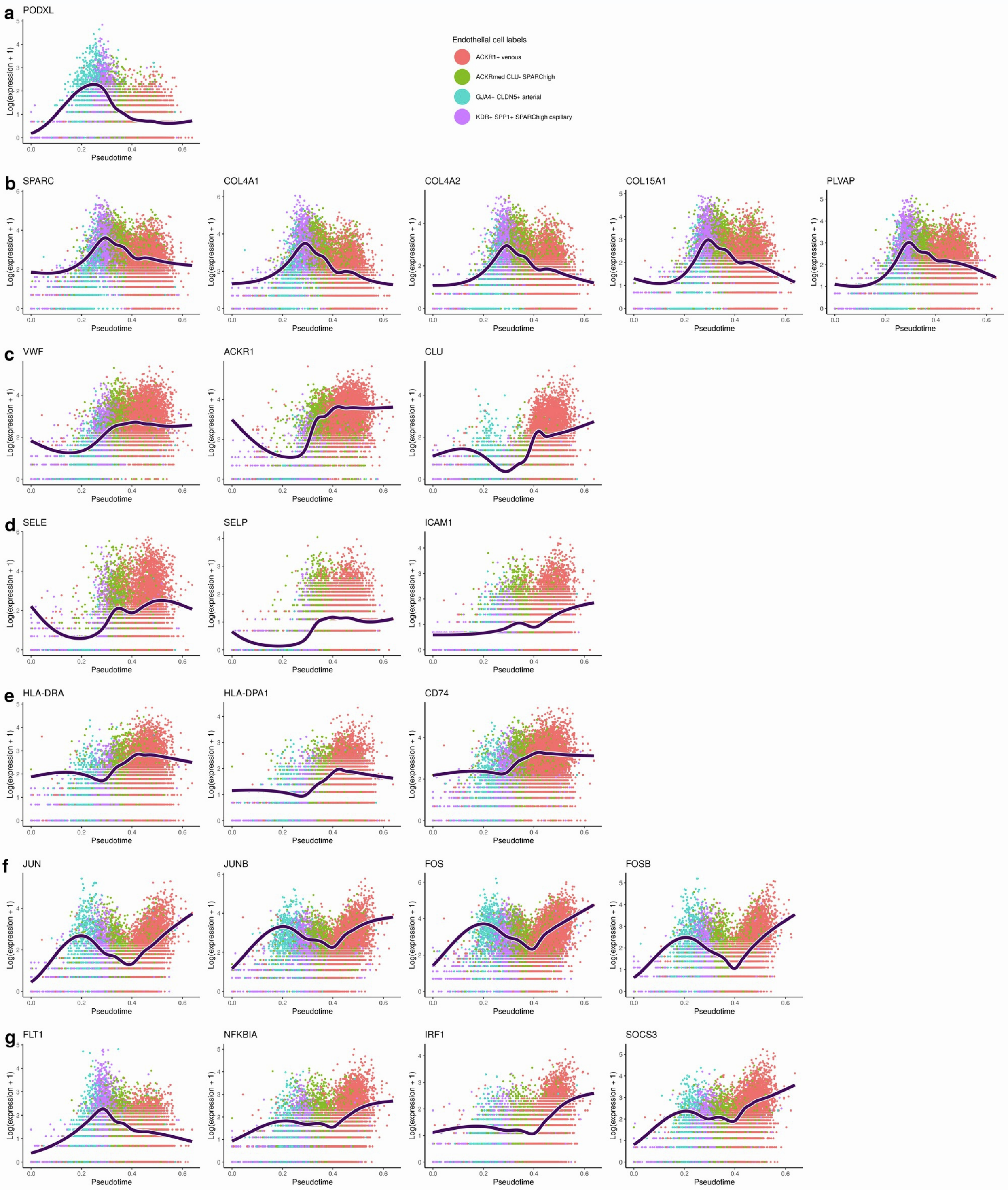

a
PODXL
Endothelial cell labels
ACKR1+ venous
ACKRmed CLU- SPARChigh
GJA4+ CLDN5+ arterial
KDR+ SPP1+ SPARChigh capillary
b
SPARC
COL4A1
COL4A2
COL15A1
PLVAP
c
VWF
ACKR1
CLU
d
SELE
SELP
ICAM1
e
HLA-DRA
HLA-DPA1
CD74
f
JUN
JUNB
FOS
FOSB
g
FLT1
NFKBIA
IRF1
SOCS3
Log(expression + 1)
Pseudotime

Figure S17

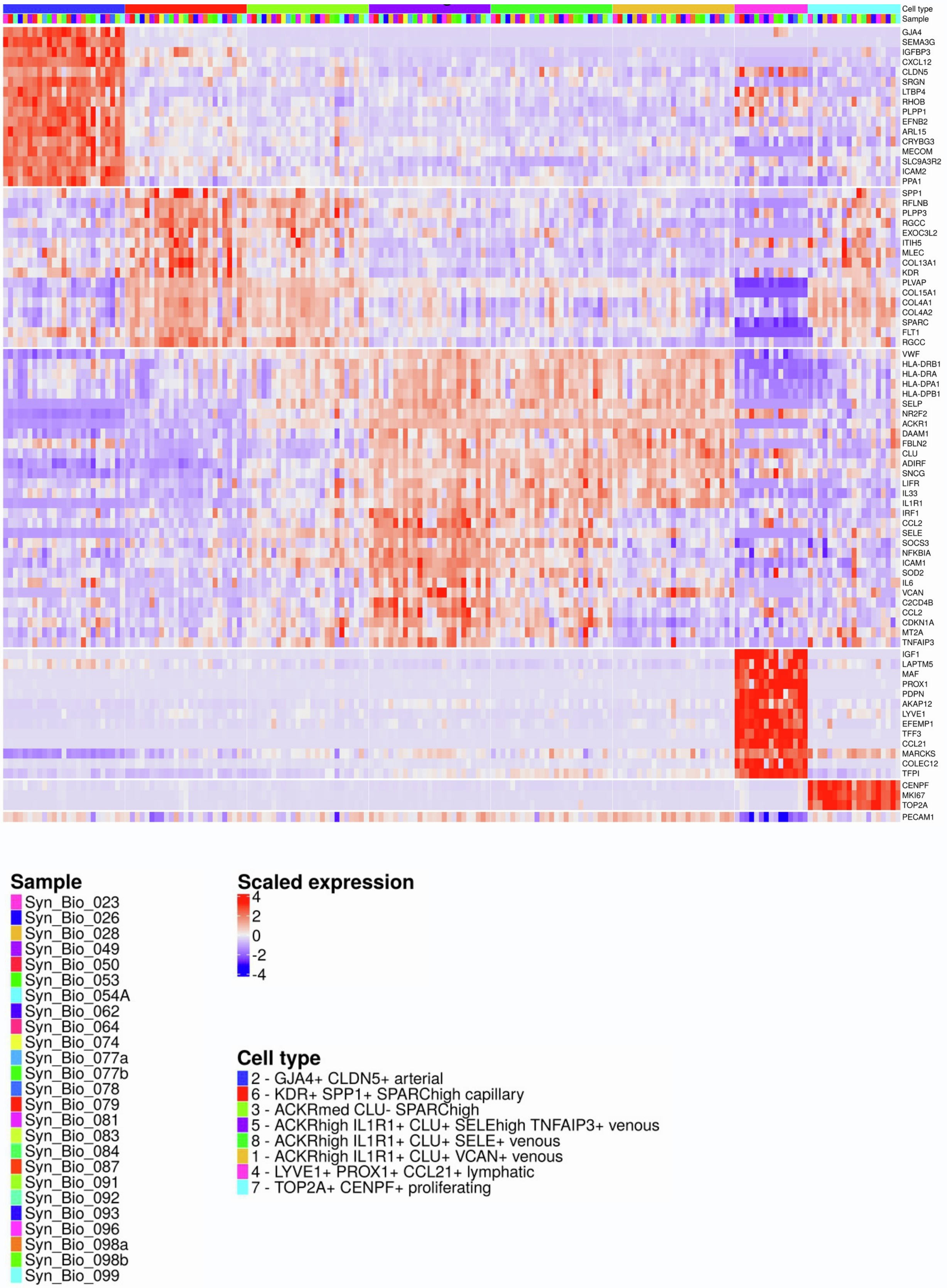

Cell type
Sample
GJA4
SEMA3G
IGFBP3
CXCL12
CLDN5
SRGN
LTBP4
RHOB
PLPP1
EFNB2
ARL15
CRYBG3
MECOM
SLC9A3R2
ICAM2
PPA1
SPP1
RFLNB
PLPP3
RGCC
EXOC3L2
ITIH5
MLEC
COL13A1
KDR
PLVAP
COL15A1
COL4A1
COL4A2
SPARC
FLT1
RGCC
VWF
HLA-DRB1
HLA-DRA
HLA-DPA1
HLA-DPB1
SELP
NR2F2
ACKR1
DAAM1
FBLN2
CLU
ADIRF
SNCG
LIFR
IL33
IL1R1
IRF1
CCL2
SELE
SOCS3
NFKBIA
ICAM1
SOD2
IL6
VCAN
C2CD4B
CCL2
CDKN1A
MT2A
TNFAIP3
IGF1
LAPTM5
MAF
PROX1
PDPN
AKAP12
LYVE1
EFEMP1
TFF3
CCL21
MARCKS
COLEC12
TFPI
CENPF
MKI67
TOP2A
PECAM1
Sample
Syn_Bio_023
Syn_Bio_026
Syn_Bio_028
Syn_Bio_049
Syn_Bio_050
Syn_Bio_053
Syn_Bio_054A
Syn_Bio_062
Syn_Bio_064
Syn_Bio_074
Syn_Bio_077a
Syn_Bio_077b
Syn_Bio_078
Syn_Bio_079
Syn_Bio_081
Syn_Bio_083
Syn_Bio_084
Syn_Bio_087
Syn_Bio_091
Syn_Bio_092
Syn_Bio_093
Syn_Bio_096
Syn_Bio_098a
Syn_Bio_098b
Syn_Bio_099
Scaled expression
4
2
0
-2
-4
Cell type
2 - GJA4+ CLDN5+ arterial
6 - KDR+ SPP1+ SPARChigh capillary
3 - ACKRmed CLU- SPARChigh
5 - ACKRhigh IL1R1+ CLU+ SELEhigh TNFAIP3+ venous
8 - ACKRhigh IL1R1+ CLU+ SELE+ venous
1 - ACKRhigh IL1R1+ CLU+ VCAN+ venous
4 - LYVE1+ PROX1+ CCL21+ lymphatic
7 - TOP2A+ CENPF+ proliferating

Figure S18

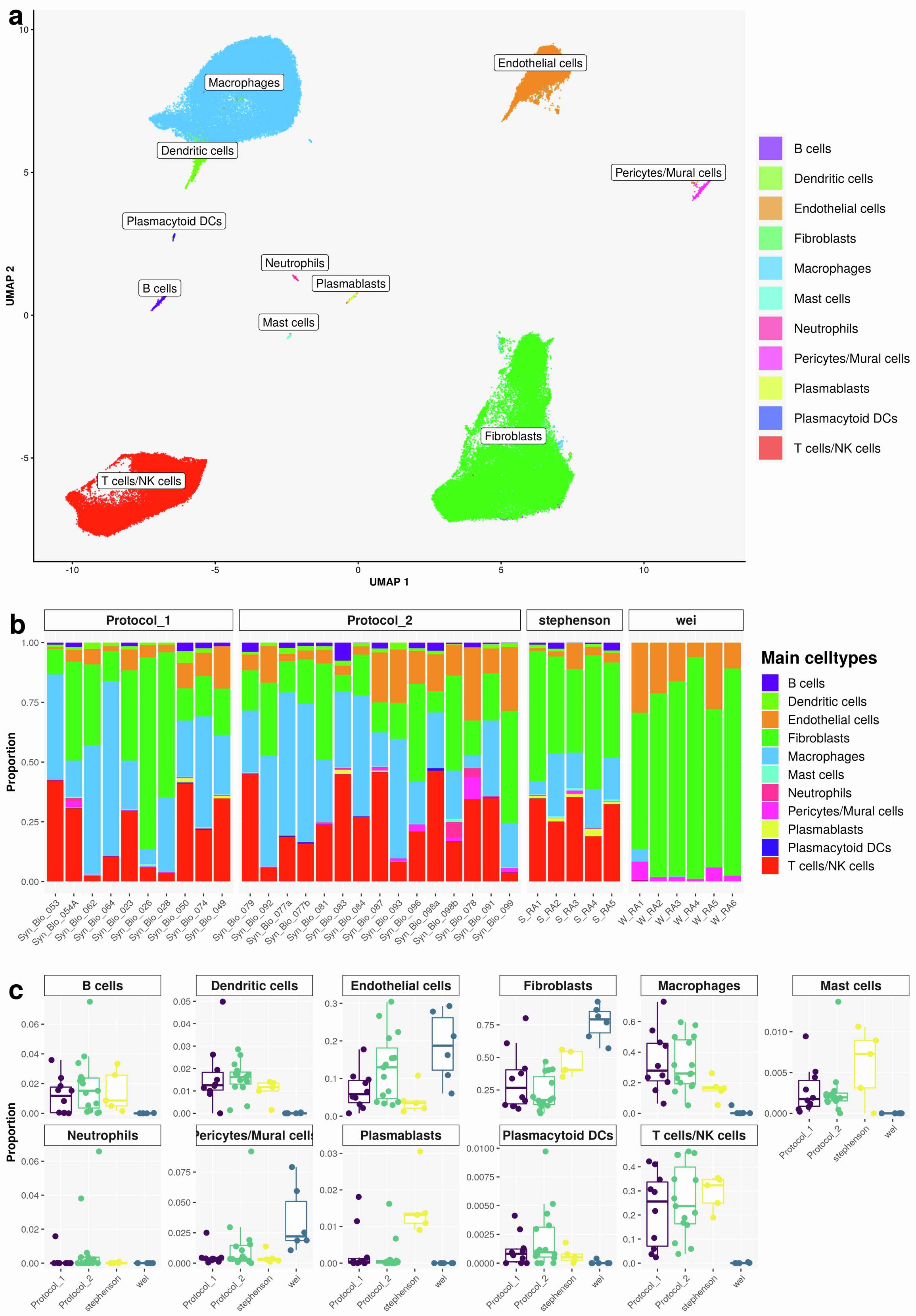
a
Macrophages
Endothelial cells
Dendritic cells
Pericytes/Mural cells
Plasmacytoid DCs
Neutrophils
Plasmablasts
B cells
Mast cells
Fibroblasts
T cells/NK cells
UMAP 1
UMAP 2
B cells
Dendritic cells
Endothelial cells
Fibroblasts
Macrophages
Mast cells
Neutrophils
Pericytes/Mural cells
Plasmablasts
Plasmacytoid DCs
T cells/NK cells
b
Protocol_1
Protocol_2
stephenson
wei
Proportion
Main celltypes
c
B cells
Dendritic cells
Endothelial cells
Fibroblasts
Macrophages
Mast cells
Neutrophils
Pericytes/Mural cells
Plasmablasts
Plasmacytoid DCs
T cells/NK cells
Protocol_1
Protocol_2
stephenson
wei

SUPPLEMENTAL FIGURES AND LEGENDS

Figure S1 Immunohistology analysis of biopsied synovial tissue affected by inflammatory arthritis. Related to Tables 1 and 2. Representative synovial biopsy images from a patient with psoriatic arthritis. **a)** Haematoxylin-eosin staining of formalin-fixed, paraffin-embedded synovial tissue demonstrating synoviocyte hyperplasia with mild chronic lymphocytic inflammation. Krenn synovitis score[S1] with value 6 (synovial lining hyperplasia: 3, fibroblast activity: 2, inflammatory infiltrate indicates middle-grade synovitis. Scale bar = 200 µm. **b)** Immunohistochemistry analysis on deparaffinized synovial tissue labeled with antibodies against endothelial cell (CD31/PECAM1), macrophage (CD68), plasma cell (CD138), and neutrophil (CD15) markers. Antigen visualization with HRP-based DAB staining. Diffuse-myeloid synovial tissue pathotype. Predominant CD68+ macrophages, localizing to synovial lining and sublining, vessels with CD31+ endothelial cells. Focal CD15+ neutrophils, and fibrin-associated CD15+ neutrophils. Scale bar = 200 µm. **c)** Comparison of the number of isolated cells and patient age per individual protocol and per both protocols. Paired age-cell number data were available for 21 samples.

Figure S2. **Quality control of integrative protocol scRNA-seq analysis of 18 human synovial biopsies of patients with inflammatory arthritis. Related to Fig. 2. a)** The number of genes vs. total counts per sample coloured by filter (left) and percentage mitochondrial counts (right). **b)** The number of cells per sample (left) and per protocol (right), coloured by the number of genes (top) and by the number of genes in at least 1% of cells (bottom). **c)** Distribution of counts per protocol (left) and per sample (right). **d)** Distribution of the number of genes per protocol (left) and per sample (right).

Figure S3. UMAPs of integrated scRNA-seq data from integrative protocol analysis showing main synovial cell types. **Related to Fig. 3.** Data derive from 18 synovial tissue biopsies of patients with inflammatory arthritis and are coloured by top marker genes of major synovial cell populations including **a)** T cells, B cells and plasmablasts; **b)** macrophages and plasmacytoid dendritic cells; **c)** myeloid dendritic cells, mast cells and neutrophils; **d)** endothelial cells, pericytes/mural cells and synovial fibroblasts.

Figure S4. UMAPs of scRNA-seq data from integrative protocol analysis showing synovial cell subsets. Related to Fig. 3. Data from 18 synovial tissue biopsies of patients with inflammatory arthritis. **a)** T cells/NK cells, color by the expression of key marker genes *CD3*, *CD4*, *CD8*, *NKG7* or by the geometric

mean of the expression of the selected gene pairs. **b)** Macrophages, color by the expression of key marker genes *MERTK, TREM2, CD206/MRC1, IL1B, CD4*8. **c)** Endothelial cells, color by the expression of key marker genes *LYVE1, CCL21, VWF*. Additionally, endothelial cells and macrophages, color by the geometric mean of the expression of the selected gene pairs. **d**) Synovial fibroblasts, color by the expression of key marker genes *PRG4, THY1, GGT5, CXCL12 and DKK3*.

Figure S5: The heatmap of major neutrophil genes, detected in the integrative protocol scRNA-seq analysis. Related to Fig. 3. Data derive from 18 synovial tissue biopsies of patients with inflammatory arthritis. Expressions are aggregated by sample and cell type.

Figure S6 Multicolor flow cytometry analysis of freshly isolated synovial tissue cells using protocol 2. Related to Fig. 3. Synovial cells were dissociated from a knee synovial biopsy of a patient with septic oligoarthritis. The cells were labeled with Zombie Green™ Fixable Viability Dye, APC anti-human CD45, BV421™ anti-human CD11b, PE anti-human CD64, BV785™ anti-human CD19, and Alexa Fluor® 700 anti-human CD3. **a**) Total synovial cells, excluding cell debris. **b**) Gating single cells and doublet exclusion. **c**) Gating live cells and excluding dead cells, with a high Zombie Green™ Fixable Viability Dye signal. **d**) Live synovial cells gated for CD45+ leukocytes (violet) and CD45- structural cells (green). **e**) Gating of CD45+ leukocytes into CD11b+ CD64+ macrophages. **f**) CD11b - CD64- cells were gated further into CD3+ CD19- T cells and CD3- CD19+ B cells. **g**) A total of 10121 events were analyzed, among which 74.2% were gated as synovial cells, consisting of 93.2% of single cells with 80.2% viability.

Figure S7 Spectral flow cytometry analysis of freshly isolated synovial tissue cells using protocol 2. Related to Fig. 3. Synovial cells were dissociated from a biopsied wrist synovial tissue of a patient with early RA. Fixed unsorted synovial cells were labeled with a cocktail of antibodies targeting surface leukocyte (CD45), myeloid (CD14), lymphocyte (CD3, CD4, CD8, CD19) and structural cell (CD31, PDPN) markers followed by analysis on spectral analyser Sony ID7000. **a)** Division of synovial cells based on the expression of CD45. CD45+ leukocytes included CD14+ macrophages, CD19+ B cells and CD3+ T lymphocytes, dividing further into CD4+ and CD8+ T cells. CD45neg synovial cells contained two structural cell clusters, including PECAM1+ endothelial cells and PDPN+ synovial fibroblasts. There was a considerable amount of cell debris, which might have been attributed to the sample shipment from Portugal to Germany. **b)** Unstained synovial cells were used for setting the flow cytometry gates.

Figure S8. Quality control of scRNA-seq analysis of the single cell reference map of fresh human synovium from 25 synovial biopsies of patients with inflammatory arthritis. Related to Figs. 4-8. a) A cell-level summary of the total number of counts, the number of detected genes and the percentage of mitochondrial counts. The number of genes vs. total counts per sample coloured by filter (left) and percentage mitochondrial counts (right). **b)** Sample summary statistics with the number of cells and number of detected genes after filtering of low-quality cells. The number of cells per sample is coloured by the number of genes (top) and by the number of genes in at least 1% of cells (bottom). **c)** Distribution of counts per sample. **d)** Distribution of the number of genes per synovial tissue sample.

Figure S9. Integrated scRNA-seq dataset from 25 synovial tissue samples from patients with inflammatory arthritis. Related to Figs. 4-8. a) UMAP of annotated main synovial cell populations coloured by main cell type. **b)** Bar plots of relative abundances of main cell types per sample coloured by main cell type. **c)** The variability of the proportion of cell types across patient synovial tissues. The box plot visualises 5 summary statistics: the median; two hinges, corresponding to the first and the third quartiles; two whiskers. The upper (lower) whisker extends from the hinge to the largest (smallest) value no further than 1.5 * interquartile range from the hinge. Individual dots represent data from different samples. See **STAR Methods** for details.

Figure S10. A heatmap of top marker genes of the main synovial cell types identified in the integrated scRNA-seq dataset. **Related to Figs. 4-8.** Data are derived from 25 synovial tissue samples from patients with inflammatory arthritis (see **STAR Methods** for details). Expressions are aggregated by sample and cell type.

Figure S11. T cell, natural killer (NK) cell and innate lymphoid cell sub clustering. Related to Fig. 4. The integrated scRNA-seq dataset consists of scRNA-seq profiles from 25 synovial tissue samples from patients with different types of inflammatory arthritis (see **STAR Methods** for details). **a)** UMAPs showing the small population of proliferating TOP2A+ CENPF+ T cells (cluster 2). **b)** A heatmap of the cluster-enriched genes and marker genes in synovial T cell, NK cell and innate lymphoid clusters in patients with inflammatory arthritides. Expressions are aggregated by sample and cell type. **c)** UMAPs demonstrating the expression of *FOXP3, PCDC1* and *CXCL13* genes in the TIGIT+ CTLA4+ T cells (cluster

4). **d)** UMAPs demonstrating the expression of granzyme (*GZMK, GZMB, GZMH*) and granulysin (*GNLY*) genes in CD8+ T cells (clusters 6-8) and NK cells (cluster 3).

Figure S12. Synovial fibroblast sub clustering. Related to Fig. 5. The integrated dataset from 25 synovial biopsies of patients with inflammatory arthritis (see **STAR Methods** for details) with **a)** violin plots showing the expression (log counts) of the lining marker PRG4 and the sublining marker THY1 across the seven synovial fibroblast clusters, and **b)** UMAPs showing the small population of proliferating TOP2A+ CENPF+ synovial fibroblasts, co-clustering with the cluster four SERPINE1+ COL5A3+ synovial fibroblasts (see also **Fig. 5a, d**).

Figure S13. A heatmap of the top cluster genes and known markers of synovial fibroblast clusters detected in the synovium of patients with inflammatory arthritides. Related to Fig. 5. The integrated scRNA-seq dataset consists of 25 synovial tissue samples from patients with different types of inflammatory arthritis (see **STAR Methods** for details). Expressions are aggregated by sample and cell type.

Figure S14. The expression of a selected set of genes in synovial fibroblast sub clustering. Related to Fig. 5. The integrated dataset from 25 synovial biopsies of patients with inflammatory arthritis (see **STAR Methods** for details) with **a)** UMAPs showing the highest enriched expression of the genes involved in MHCII class mediated antigen presentation in HLA-DRA^{high} synovial fibroblasts (cluster 7), and **b)** UMAPs showing the enriched expression of *IL6* and *NOTCH3* genes primarily in sublining GGT5+ synovial fibroblasts (clusters 6).

Figure S15. Synovial macrophage and myeloid dendritic (DC) cell sub clustering. Related to Fig. 6. A heatmap of the top cluster genes and known marker genes of synovial macrophage and DK subclusters, detected in the integrated scRNA-seq dataset from 25 synovial tissue samples of patients with different types of inflammatory arthritis (see **STAR Methods** for details). Expressions are aggregated by sample and cell type.

Figure S16 Trajectory analysis of synovial endothelial cells. Related to Fig. 7. Inferred pseudotime versus gene expression in synovial endothelial cells. Start of the trajectory are GJA4+ CLDN5+ arterial

endothelial cells, end of the trajectory are venous endothelial cells. The color represents an endothelial cell subset, while the black curve represents the fitted model. The identified genes encode **a-c)** main endothelial cell subset markers; **d,e**) genes encoding molecules with subset-specific endothelial cell functions and **f, g**) genes encoding key components of different signaling pathways, which appear differentially enriched across EC subsets.

Figure S17. Endothelial cell sub clustering. Related to Fig. 7. Heatmap of the top cluster genes and known markers of synovial vascular and lymphatic endothelial cell subclusters detected in the integrated scRNA-seq dataset from 25 synovial tissue samples of patients with different types of inflammatory arthritis (see **STAR Methods** for details). Expressions are aggregated by sample and cell type.

Figure S18. Integration of our synovial scRNA-seq from fresh synovium with publicly available synovial scRNA-seq datasets. Related to Fig. 9. We integrated our scRNA-seq data from 25 synovial tissue samples of patients with inflammatory arthritis with published data from Stephenson W and colleagues[S2] and Wei K et colleagues[S3] (see **STAR Methods** for details). **a)** UMAP of annotated main synovial cell populations coloured by main cell type across the three studies. **b)** Bar plots of relative abundances of main cell types per sample coloured by main cell types across the 3 studies. **c)** The variability of the proportion of cell types across patient synovia in the 3 studies. The box plot visualises 5 summary statistics: the median; two hinges, corresponding to the first and the third quartiles; two whiskers. The upper (lower) whisker extends from the hinge to the largest (smallest) value no further than 1.5 * interquartile range from the hinge. Individual dots represent data from different samples. Our data and data from Stephenson W et al. [S2] is derived from unsorted synovial cells, while Wei K et al. [S3] dataset includes sorted $CD45^{neg}$ $CD235^{neg}$ synovial fibroblasts, pericytes/mural cells and synovial endothelial cells.

SUPPLEMENTAL TABLES AND LEGENDS

**Pat.-ID.**	**Details on neutrophil histology, based on CD15 staining.**	**Proportion (%) of neutrophils (scRNA-seq).***	**Histology. Neutrophil detection.**	**ScRNA-seq. Neutrophil detection.**
**SB-023 P1**	Focal, in addition fibrin-associated neutrophils	0.000173	Y	y
**SB-026 P1**	Single fibrin-associated neutrophils	0	Y	N
**SB-028 P1**	Fibrin-associated neutrophils	0	Y	N
**SB-049 P1**	No CD15 staining	0	N	N
**SB-050 P1**	Focally interspersed neutrophils	0	Y	N
**SB-074 P1**	No CD15 staining	0	N	N
**SB-077A P2**	Up to 10 neutrophils per HPF (0.26mm2)	0	Y	N
**SB-077B P2**	1 fragment with neutrophils, up to 20 per HPF (0.26mm2)	0	Y	N
**SB-079 P2**	<2 per HPF (0.26mm2)	0	Y	N
**SB-081 P2**	Up to 15 neutrophils per HPF (0.26mm2)	0.004424	Y	Y
**SB-083 P2**	Fibrin-associated neutrophils	0.007027	Y	Y
**SB-084 P2**	Vessel-associated neutrophils	0.000757	Y	Y

**SB-087 P2**	Focal florid inflammation with more than 35 neutrophils per HPF (0.26mm2)	0.002684	Y	Y
**SB-092 P2**	Focal small infiltration	0	Y	N
**SB-093 P2**	Predominantly fibrin-associated neutrophils with transition into the synovial membrane, up to 5 per HPF (0.26mm2)	0.000206	Y	Y
**SB-096 P2**	No CD15 staining	0.001002	N	Y
**SB-098a P2**	Up to 38 neutrophils per HPF (0.26mm2)	0	Y	N
**SB-098b P2**	Fibrin-associated neutrophils	0.056911	Y	Y

Table S1 Detection of neutrophils in 18 synovial tissue biopsies included in integrative protocol analysis using histology and scRNA-seq analyses. Related to Figure 2. For the detection of neutrophils in formalin-fixed, paraffin-embedded synovial biopsy fragments, tissue sections were labeled with CD15 antibodies (see **STAR Methods** for details). In scRNA-seq data, the quantity of neutrophils was estimated by calculating the proportion of neutrophils per sample. Data pertains to 18 samples included in integrative protocol analysis. Y: yes, N: no.

Flow cytometry technology	Multicolor	Spectral
**Gender**	M	F
**Age**	46	66
**Diagnosis**	Septic oligoarthritis (*N. gonorrhoeae*)	Early RA
**Therapy at biopsy**	Treatment naive	Treatment naive
**Krenn synovitis score**[S1]	5	7
**Dissociation protocol**	2	2
**N (isolated cells)**	200’000	346’000
**Cell viability***	86%	90,4%
**Markers analyzed**	CD45, CD11B, CD64, CD3, CD19, Fixable viability dye	CD45, CD14, CD3, CD4, CD8, CD19, PDPN, CD31

Table S2. Demographic, clinical, therapy, histology and cell characteristics for 2 synovial tissue biopsy samples from patients with arthritis, included in the proof of principle flow cytometry analyses. Related to Figures 6, 7. F: female, M: male, *Automatic cell counting with the Luna-FL Dual Fluorescence Cell Counter.

SUPPLEMENTAL ITEM REFERENCES

S1 Krenn, V., Morawietz, L., Häupl, T., Neidel, J., Petersen, I., and König, A. (2002). Grading of chronic synovitis—a histopathological grading system for molecular and diagnostic pathology. Pathology-Research and Practice *198*, 317–325. 10.1078/0344-0338-5710261

S2 Stephenson, W., Donlin, L.T., Butler, A., Rozo, C., Bracken, B., Rashidfarrokhi, A., Goodman, S.M., Ivashkiv, L.B., Bykerk, V.P., Orange, D.E., et al. (2018). Single-cell RNA-seq of rheumatoid arthritis synovial tissue using low-cost microfluidic instrumentation. Nat Commun *9*, 791. 10.1038/s41467-017-02659-x.

S3 Wei, K., Korsunsky, I., Marshall, J.L., Gao, A., Watts, G.F.M., Major, T., Croft, A.P., Watts, J., Blazar, P.E., Lange, J.K., et al. (2020). Notch signalling drives synovial fibroblast identity and arthritis pathology. Nature *582*, 259–264. 10.1038/s41586-020-2222-z.
